# Major Transcriptome Changes Accompany the Growth of *Pseudomonas aeruginosa* in Blood from Patients with Severe Thermal Injuries

**DOI:** 10.1371/journal.pone.0149229

**Published:** 2016-03-02

**Authors:** Cassandra Kruczek, Kameswara Rao Kottapalli, Sharmila Dissanaike, Nyaradzo Dzvova, John A. Griswold, Jane A. Colmer-Hamood, Abdul N. Hamood

**Affiliations:** 1 Department of Surgery, School of Medicine, Texas Tech University Health Sciences Center, Lubbock, Texas, United States of America; 2 Center for Biotechnology and Genomics, Texas Tech University, Lubbock, Texas, United States of America; 3 Department of Immunology and Molecular Microbiology, School of Medicine, Texas Tech University Health Sciences Center, Lubbock, Texas, United States of America; 4 Department of Medical Education, School of Medicine, Texas Tech University Health Sciences Center, Lubbock, Texas, United States of America; East Carolina University School of Medicine, UNITED STATES

## Abstract

*Pseudomonas aeruginosa* is a Gram-negative opportunistic pathogen that causes serious infections in immunocompromised hosts including severely burned patients. After multiplying within the burn wound, *P*. *aeruginosa* translocate into the bloodstream causing bacterial sepsis frequently leading to organ dysfunction and septic shock. Although the pathogenesis of *P*. *aeruginosa* infection of thermally-injured wounds has been extensively analyzed, little is known regarding the ability of *P*. *aeruginosa* to adapt and survive within the blood of severely burned patients during systemic infection. To identify such adaptations, transcriptome analyses (RNA-seq) were conducted on *P*. *aeruginosa* strain PA14 that was grown in whole blood from a healthy volunteer or three severely burned patients. Compared with growth in blood from healthy volunteers, growth of PA14 in the blood from severely burned patients significantly altered the expression of 2596 genes, with expression of 1060 genes enhanced, while that of 1536 genes was reduced. Genes whose expression was significantly reduced included genes related to quorum sensing, quorum sensing-controlled virulence factors and transport of heme, phosphate, and phosphonate. Genes whose expression was significantly enhanced were related to the type III secretion system, the pyochelin iron-acquisition system, flagellum synthesis, and pyocyanin production. We confirmed changes in expression of many of these genes using qRT-PCR. Although severe burns altered the levels of different blood components in each patient, the growth of PA14 in their blood produced similar changes in the expression of each gene. These results suggest that, in response to changes in the blood of severely burned patients and as part of its survival strategy, *P*. *aeruginosa* enhances the expression of certain virulence genes and reduces the expression of others.

## Introduction

Severe burn is one of the most serious forms of trauma. Besides the destruction of the skin barriers by the thermal injury, severely burned patients are immunocompromised due to the repression of both local and systemic immune responses [1,2]. As a result, severe burns are often associated with infectious complications. The avascular connective tissues within the burn wound impair the migration of host immune cells and limit the delivery of systemically administered antimicrobial agents [3–5]. Local host immune responses are further impaired by the toxic substances released by the necrotic tissues [4]. Immediately following the thermal injury, the wound surface is sterile [4]. However it is quickly colonized by Gram-positive and Gram-negative bacteria that reach the wound from the patient’s skin, gastrointestinal tract, or respiratory tracts; healthcare-associated human contacts; or from the external environmental surfaces, water, fomites, and air [4,6,7]. Colonizing bacteria multiply, establish an infection, and translocate from the infected burn wound into the bloodstream causing bacteremia and sepsis [8,9]. In the intensive care unit, the most common complication that occurs in burn patient is bloodstream infection followed by sepsis, septic shock, and multiple organ failure [4,10]. In modern burn units, more than 50% of deaths are due to septic shock and organ dysfunction [11–13]. Among the different pathogens that cause sepsis in burn patients is the opportunistic pathogen *Pseudomonas aeruginosa* that also infects other immunocompromised hosts such as individuals with cystic fibrosis (CF) or with cancer [4,14–17]. *P*. *aeruginosa* produces numerous cell-associated and extracellular virulence factors including lipopolysaccharides, flagellum, pili, exotoxin A (ETA), elastases, alkaline protease, exoenzyme S, and pyocyanin as well as others [14,15,17,18].

In response to the environment at specific infection sites and to adapt to that specific environment, *P*. *aeruginosa* produces different virulence factors. For example, previous studies showed that over the course of CF airway infection and as an adaptation to the chronic infections, *P*. *aeruginosa* isolates undergo specific phenotypic changes that include the lack of swimming motility [19], increased phage resistance [20], over-production of alginate [21,22], lack of pyoverdin and pyochelin production [23], and lack of expression of the type III secretion system (T3SS) [24]. Using whole genome analysis, Smith *et al*. [25] showed that during chronic airway infection of CF patients, *P*. *aeruginosa* undergoes numerous genetic changes. Genes that code for virulence factors that are required to initiate acute infection were selected against as the infection became chronic [25]. The most frequently mutated (selected against) genes were those that code for multidrug efflux pumps, *lasR*, the virulence factor regulator v*fr*, and the T3SS regulator *exsA* [25]. The loss of LasR function provided *P*. *aeruginosa* with a growth advantage with respect to certain carbon and nitrogen sources [26]. Recently, Dingemans *et al*. [27] provided evidence indicating that the adaptation of *P*. *aeruginosa* to the CF lung included deletion within the genes coding for the pyoverdine receptor *fpvA* and other *tonB*-receptors as well as T3SS genes and additional virulence genes. In contrast to the deletion within the quorum sensing (QS) genes (*e*.*g*., *lasR*) during chronic CF lung infection, successful infection of thermally-injured wounds requires functional QS systems. We previously showed that, compared with thermally-injured mice infected with the wild type strain PAO1, the mortality rate among thermally-injured mice infected with QS mutants was significantly reduced [28]. Recently, using the same model, as well as the murine chronic wound model, and comparing each model to MOPS-succinate medium through RNA-seq analysis, Turner *et al*. [29] showed that the expression of 11 *P*. *aeruginosa* PAO1 pyochelin and 17 pyoverdine genes were significantly enhanced in the acute wound, but only four pyochelin and five pyoverdine genes were significantly enhanced in the chronic wound.

The transition of pathogenic bacteria from local infection sites to invasive infection is associated with changes in the environment to which the organism is exposed. To adapt to these changes, pathogenic bacteria likely alter their virulence by varying the production of different virulence factors. Graham *et al*. [30] previously addressed the transition of group A *Streptococcus* from the throat or infected skin to blood by analyzing the adaptation of the bacteria to the growth in blood. Growth of group A *Streptococcus* in human blood resulted in a rapid increase in the transcription of many genes essential for bacterial dissemination including those encoding superantigens and host evasion proteins [30]. While the pathogenesis of *P*. *aeruginosa* infection of thermally-injured wounds is extensively analyzed, little is known regarding the adaptive mechanisms of *P*. *aeruginosa* as it translocates from the infected wound to the bloodstream of severely burned patients. Severe thermal injuries cause numerous changes in blood components; following burn injury, the production and release of monocytes from the bone marrow are decreased, neutrophil chemotaxis and intracellular killing are impaired, and the alternative pathway of the complement cascade is depressed [4]. Both hypophosphatemia and hypomagnesemia occur in patients with severe thermal injuries [31,32]. In this study, and as part of our efforts to understand the adaptive mechanisms of *P*. *aeruginosa* to the bloodstream environment during bacteremia, we compared the transcriptome of the *P*. *aeruginosa* strain PA14 grown in blood from healthy volunteers with that of PA14 grown in blood from severely burned patients.

## Results and Discussion

Previous *in vitro* studies utilized laboratory media that were designed to maximally produce specific *P*. *aeruginosa* virulence factors. In this study, we utilized human blood as a growth medium. We grew *P*. *aeruginosa* in whole blood from healthy volunteers or severely burned patients and compared the expression of different genes between the two groups. To minimize variations among blood samples from the burned patients and to ensure the inclusion of injury-induced changes in blood in each sample, we used the following stringent criteria for patient selection. (1) Only patients with a burn injury affecting 20% or greater of the total body surface area (TBSA) were selected. (2) Blood samples were obtained within 72 hours of the initial injury. We considered this period as the window for any potential injury-induced changes in blood. (3) To eliminate the influence of antibiotics on *P*. *aeruginosa* gene expression, samples were obtained prior administration of systemic antibiotic treatment. For this study, we collected whole blood from three patients whose burn TBSA varied between 25 to 50% (Table 1). As a control, we utilized whole blood from healthy volunteers.

**Table 1:** Characteristics of the three burn patients.

	Age (y)	Sex	% TBSA^a^	Burn severity	Source	Time post-injury^b^
Patient 1	28	Male	30%	2^nd^ and 3^rd^ degree	Heat (car fire)	24 h
Patient 2	62	Male	50%	2^nd^ and 3^rd^ degree	Heat (flash explosion)	48 h
Patient 3	43	Male	25%	2^nd^ degree	Heat (gas explosion)	72 h

^a^Percentage of total body surface area involved in burn

^b^Time to collection of blood sample

### Adjusting the Parameters of *P*. *aeruginosa* Growth in Whole Blood

We utilized the closed loop flow through (CLFT) system described by Kealey *et al*. [33] for the examination platelet deposition on arterial stents for culturing *P*. *aeruginosa* in whole blood. In our CLFT system, the blood was circulated by a peristaltic pump at a speed that resembles the pumping of blood through vessels in the human body; the blood was circulated in 16-mm platinum-cured silicone tubing (Cole-Parmer Instruments, Vernon Hills, IL) to allow oxygen diffusion, and the tubing was incubated at human body temperature (37°C) by placing it in a water bath (S1 Fig) [34].

We utilized the *P*. *aeruginosa* strain UCBPP-PA14 (PA14) in all of the experiments. This strain, which was originally isolated from an infected burn wound [35], has been used extensively in both *in vivo* and *in vitro* studies [36–39] and is fully virulent in the murine model of thermal injury, causing bacteremia and death within 24–48 h post infection [35]. Whole blood samples, 8.3 mL/tube, were collected in BD Vacutainer^™^ tubes containing 1.7 mL 0.35% sodium polyanetholsulfonate (SPS) as an anticoagulant (BD Medical, Franklin Lakes, NJ). The final concentration of SPS is 0.06%. SPS has been added to blood culture media such as supplemented peptone broth and tryptic soy broth at a final concentration of 0.05 to 0.06% for decades [40,41]. Preliminary analysis revealed that SPS at 0.06%, whether added to the nutrient rich growth medium LB broth or when whole blood was used as the culture medium, did not interfere with the growth of *P*. *aeruginosa*. From an initial inoculum of 10^2^ colony forming units (CFU)/mL, PA14 grew to 10^8^ CFU/mL by 8 h post-inoculation (a 6-log increase). While the growth was less than that in the rich medium LB broth, these results confirmed that we could obtain sufficient growth of PA14 in whole blood for the gene expression experiments.

Lysis of red blood cells (RBC) was another factor that complicated our experiments. We detected the least RBC lysis when blood samples were kept at room temperature and used within 2 h of collection [34]. To minimize RBC lysis, all whole blood-related experiments were performed immediately after collection of blood from patients or volunteers. We also investigated the possibilities that the growth of PA14 in whole blood would cause either extensive RBC lysis or a considerable change in the pH of the blood culture. At 8 and10 h post inoculation, the percentage of RBC lysis was 7 and 14%, respectively, but rose to 71% at 12 h [34]. Normal pH of blood ranges from 7.35 to 7.45. Patients with metabolic alkalosis have 45% mortality at pH 7.55 and 80% at 7.65. The pH could also affect the growth of PA14, so we determined the effect of PA14 growth on the pH of the blood culture over time. The pH remained unchanged at 7.4 for 8 h post-inoculation, but by 10 h post inoculation, the pH increased to 7.7, well into significant alkalosis [34].

Based on these findings, we grew PA14 in 7.5 mL of SPS-anticoagulated whole blood in a 2-ft loop of 16-mm platinum-cured silicone tubing submerged in a 37°C water bath. The blood was circulated at 176 mL/min. An initial inoculum of 10^2^ CFU/mL of PA14 was used. The blood cultures were harvested at 8 h post inoculation and the bacteria were collected for RNA extraction.

### Growth in Blood from Severely Burned Patients Significantly Alters the Expression of Numerous PA14 Genes

**Fig 1:**
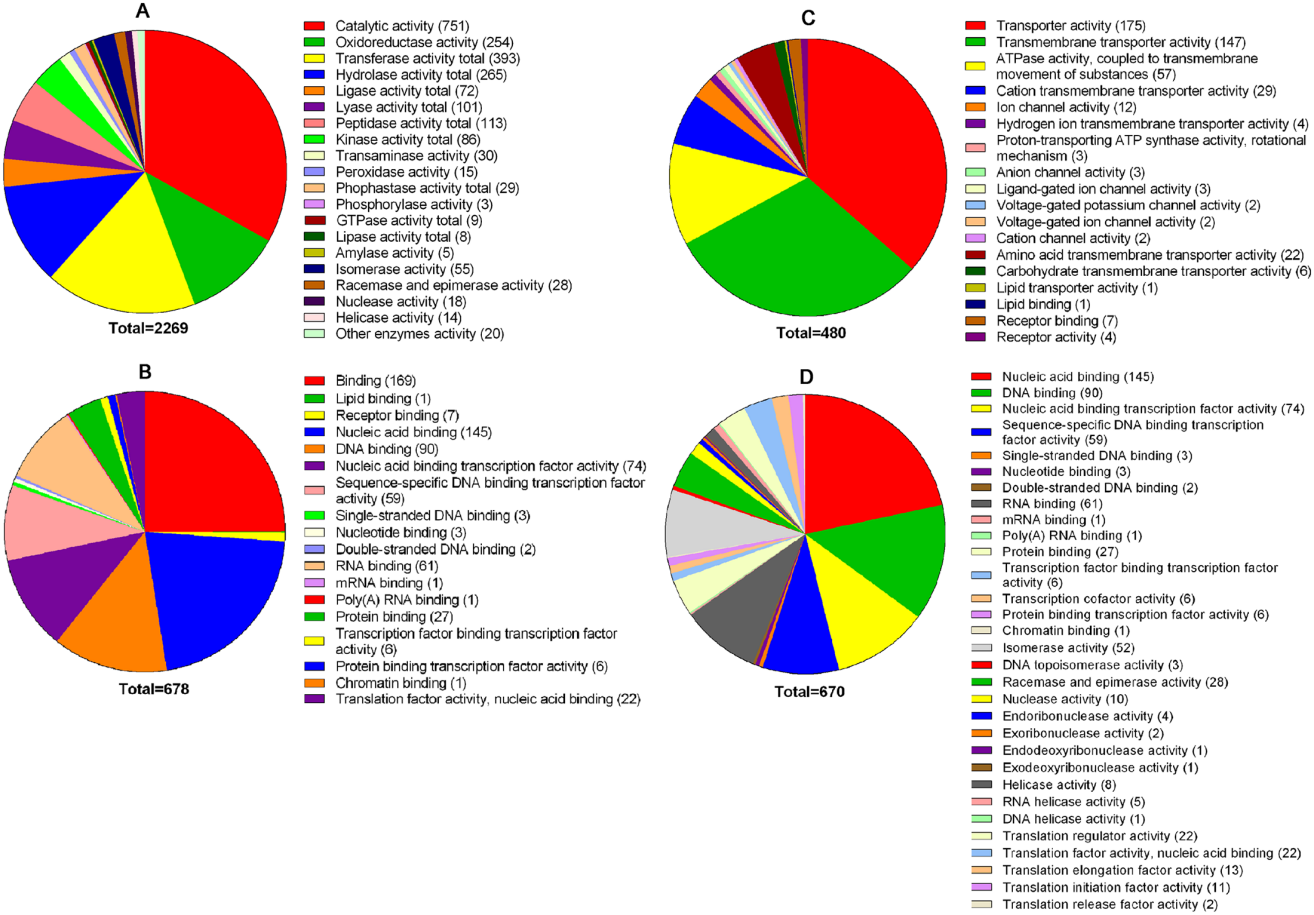
Gene Ontology (GO) molecular function terms assigned to the differentially expressed genes. Genes whose expression was differentially regulated when the bacteria were grown in whole blood from the patients compared to the healthy volunteer were analyzed using PANTHER GO-Slim Molecular Function analysis [42,43]. A total of 4949 GO terms were assigned to the 2348 mapped genes, with 1425 of these being unclassified. The remaining 3524 terms represented enzymatic activity—2269 **(A)**, substrate binding—678 **(B)**, transporter activity—480 **(C)**, and functions related to transcription, translation, and DNA replication—670 **(D)**. Some functions were assigned to more than one category.

We examined the expression of PA14 genes through RNA-sequencing utilizing the Illumina MiSeq platform (San Diego, CA). Paired end sequencing using the V2 chemistry resulted in 25.8 million 150 bp reads which were quality filtered using the Qseq software (DNA STAR, Madison, WI). Reads assigned per kilobase of target per million mapped reads (RPKM) normalization was done prior to quantification of gene expression by QSeq (DNA STAR). Genes were identified as differentially expressed with ≥ two-fold change if they had the false discovery rate correction *p*-value of ≤ 0.01. The growth of PA14 in blood from all three patients significantly increased or decreased the expression of 2596 genes compared with their expression when PA14 was grown in blood from healthy volunteers (data for each of the 6 samples have been deposited in the BioProject at NCBI (http://www.ncbi.nlm.nih.gov/bioproject/ under PRJNA287707 [accessed 03Dec2015]). Of the total affected genes, 1060 were upregulated and 1536 were down regulated. The levels of increases or decreases were comparable with each of the three burn patient samples. However, while variations among healthy volunteers were considerable, we also obtained consistent results when we compared gene expression in PA14 that was grown in blood from all three patients with that obtained from each of the other two volunteers separately. We performed Gene List Analysis for gene ontology (GO) terms associated with the upregulated PA14 genes using the PANTHER (Protein ANalysis THrough Evolutionary Relationships) Classification System [42]. The *Pseudomonas aeruginosa* PAO1 reference genome was used in the statistical overrepresentation test with default settings. To maintain a workable number of terms, we used the annotation data sets PANTHER GO-Slim Biological Process, PANTHER GO-Slim Cellular Component, and PANTHER GO-Slim Molecular Function (PANTHER version 10.0 Released 2015-05-15) [43]. We used the “Orthologs” annotation feature of the *Pseudomonas* Genome Database to match PA14 genes (UCBPP-PA14 reference) to PAO1 genes (PAO1 reference) [44,45]. Upon replacement of the PA14 gene names/numbers with the PAO1 ortholog numbers, we were able to map 2348 of the 2596 PA14 genes with GO terms. We identified 3524 GO terms associated with molecular functions (Fig 1), 5468 GO terms for biological processes (S2 Fig), and 601 GO terms with cellular components (S3 Fig). Among the 3524 assigned molecular function terms, the majority (2269) were related to enzymatic activity (Fig 1A), with 678 terms related to substrate binding (Fig 1B), 480 terms related to transporter activity (Fig 1C), and 670 terms related to transcription, translation, and DNA replication functions (Fig 1D).

### Growth in Blood from Severely Burned Patients Represses the Expression of Several PA14 QS and QS-Related Genes

RNA-seq analysis showed that the growth of PA14 in blood from all patients either repressed or had no significant effect on the expression of many QS genes; for example, the expression of *lasR*, *rhlR*, and three genes of the *pqsA-E* operon (*pqsA*, *pqsB*, and *pqsC*) was reduced by two- to four-fold (Table 2). In contrast, expression of *pqsH* which codes for the FAD-dependent mono-oxygenase [46] that is not part of the *pqsA-E* operon, increased by five-fold (Table 2). Furthermore, the expression of several Las and Rhl QS-regulated genes was repressed much more prominently than that of the QS genes themselves. The expression of *lasA*, *rhlA*, and *rhlB* was reduced by at least 71-, 11-, and ten-fold, respectively; although *plcB*, which is regulated through both the *las* and *rhl* systems was only downregulated two- to three-fold (Table 2). Interestingly, the genes within the PQS-controlled *phzA1-G1* and *phzA2-G2* operons were upregulated two- to six-fold.

**Table 2:** QS and QS-controlled genes differentially expressed in PA14 grown in whole blood from severely burned patients.

Gene	Product name	Pt 1	Pt 2	Pt 3
	Functional classification(s) // Gene ontology (GO) terms			
	Pathways // Functional prediction(s)^a^			
*lasR*	**Transcriptional Regulator LasR**	-2^b^	-2	-2
	Transcriptional regulators // Regulation of transcription, DNA-templated; Sequence-specific DNA-binding transcription factor activity			
	// Transcription factor LuxR-like, autoinducer-binding domain			
*lasB*	**Elastase LasB**	-4	-4	-4
	Secreted factors; translation, post-translational modification, degradation; amino acid biosynthesis and metabolism // Proteolysis; metalloendopeptidase activity			
	// Thermolysin metalloprotease (M4) family signature			
*lasA*	**LasA protease**	-83	-71	-76
	Secreted factors; translation, post-translational modification, degradation // proteolysis; endopeptidase activity; metalloendopeptidase activity			
	// Peptidase M23A, B-lytic metalloendopeptidase			
*rhlR*	**Transcriptional regulator RhlR**	-4	-4	-4
	Transcriptional regulators // Regulation of transcription, DNA-templated; sequence-specific DNA-binding transcription factor activity			
	// Transcription factor LuxR-like, autoinducer-binding domain			
*rhlA*	**Rhamnosyltransferase chain A**	-12	-11	-12
	Secreted factors //			
	// Alpha/beta hydrolase family			
*rhlB*	**Rhamnosyltransferase chain B**	-11	-11	-11
	Secreted factors // Metabolic process; carbohydrate metabolic process; lipid glycosylation; transferase activity (hexosyl groups)			
	Peptidoglycan biosynthesis // UDP-glucoronosyl and UDP-glucosyl transferase			
*plcB*	**Phospholipase C, PlcB**	-3	-3	-2
	Hypothetical, unclassified, unknown // Chemotaxis; phospholipase C activity; hydrolase activity (ester bonds)			
	// Phospholipase C/P1 nuclease domain			
*pqsH*	**FAD-dependent monooxygenase**	5	5	4
	Biosynthesis of cofactors, prosthetic groups, and carriers // Metabolic process; secondary metabolite biosynthetic process			
	// Aromatic-ring hydroxylase (flavoprotein monooxygenase) signature; FAD binding domain			
*pqsC*^c^	**PqsC**	-2	-2	-2
	Hypothetical, unclassified, unknown; biosynthesis of cofactors, prosthetic groups, and carriers // Metabolic process; secondary metabolite biosynthetic process; lipid biosynthetic process; fatty acid biosynthetic process; catalytic activity; 3-oxoacyl-[acyl-carrier-protein] synthase activity			
	Fatty acid biosynthesis // 3-Oxoacyl-[acyl-carrier-protein (ACP)] synthase III			
*pqsB*	**PqsB**	-2	-2	-2
	Hypothetical, unclassified, unknown; biosynthesis of cofactors, prosthetic groups, and carriers // Metabolic process; secondary metabolite biosynthetic process; catalytic activity			
	// Thiolase-like			
*pqsA*	**PqsA**	-2	-2	-2
	Hypothetical, unclassified, unknown; biosynthesis of cofactors, prosthetic groups, and carriers // Metabolic process; secondary metabolite biosynthetic process; catalytic activity			
	// AMP-dependent synthetase/ligase			
*phzH*	**Potential phenazine-modifying enzyme**	*2*	*2*	*2*
	Putative enzymes // Metabolic process; asparagine biosynthetic process; asparagine synthase activity (glutamine-hydrolyzing)			
	Phenazine biosynthesis // Glutamine amidotransferase type 2 domain			
*phzS*	**Hypothetical protein**	*2*	*2*	*2*
	Putative enzymes // Metabolic process; oxidoreductase activity			
	Phenazine biosynthesis // aromatic-ring hydroxylase (flavoprotein monooxygenase signature; FAD binding domain			
*phzG1*^c^	**Pyrodoxamine 5'-phosphate oxidase**	*2*	*2*	*2*
	Secreted factors // Phenazine biosynthetic process; oxidation-reduction process; pyridoxine biosynthetic process; FMN binding; pyridoxamine-phosphate oxidase activity; oxidation-reduction activity, acting on CH-NH2 group			
	Phenazine biosynthesis; vitamin B6 metabolism // Pyridoxamine 5’-phosphate oxidae			
*phzF1*	**PhzF**	3	3	4
	Secreted factors // Biosynthetic process; catalytic activity			
	Phenazine biosynthesis // Phenazine biosynthesis PhzF protein			
*phzE1*	**Phenazine biosynthesis protein PhzE**	4	4	4
	Secreted factors // Biosynthetic process			
	Phenazine biosynthesis // Chorismate binding enzyme; anthranilate synthase component II signature; glutamine amidotransferase			
*phzD1*	**Phenazine biosynthesis protein PhzD**	6	6	6
	Secreted factors // Metabolic process; isochorismatase activity; catalytic activity			
	Phenazine biosynthesis // Isochorismatase signature			
*phzC1*	**Phenazine biosynthesis protein PhzC**	4	4	4
	Secreted factors // Aromatic amino acid family biosynthetic process; 3-deoxy-7-phosphoheptulonate synthase activity			
	Phenylalanine, tyrosine and tryptophan biosynthesis // DAHP synthetase, class II			
*phzG2*^c^	**Pyridoxamine 5'-phosphate oxidase**	3	3	3
	Secreted factors // Oxidation-reduction process; pyridoxine biosynthetic process; oxidoreductase activity, acting on the CH-NH2 group; FMN binding; pyridoxamine-phosphate oxidase activity			
	Phenazine biosynthetic process; vitamin B6 metabolism / Pyridoxamine 5'-phosphate oxidase			
*phzF2*	**Phenazine biosynthesis protein**protein	3	3	3
	Secreted factors // Biosynthetic process; catalytic activity			
	Phenazine biosynthesis // Phenazine biosynthesis PhzF			
*rsaL*	**Regulatory protein RsaL**	-9	-8	-7
	Transcriptional regulators; adaptation, protection // Regulation of transcription, DNA-templated; positive-regulation single-species biofilm formation; negative-regulation of cytolysis in other organism; negative-regulation of elastin catabolism; negative-regulation of cell motility; negative-regulation of secondary metabolism and biosynthesis; quorum sensing; DNA binding			
	// Lambda repressor-like, DNA-binding domain			
*mvaT*	**Transcriptional regulator MvaT, P16 subunit**	-4	-3	-3
	Transcriptional regulators // No GO terms listed			
	//			
*rsmA*	**Carbon storage regulator**	-2	-2	-2
	Transcriptional regulators; translation, post-translational modification, degradation; adaptation, protection // Regulation of carbohydrate metabolic process; mRNA catabolic process; RNA binding			
	Two-component system // Carbon storage regulator [VsrA]			
*PA14_30580*	**LuxR family transcriptional regulator**	-3	-3	-4
	Transcriptional regulators // Regulation of transcription, DNA-templated; sequence-specific DNA binding transcription factor activity			
	// Signal transduction response regulator, C-terminal effector; LuxR bacterial regulatory protein HTH signature			

^a^Product names, functional classification(s), gene ontology terms, pathways, and functional predictions for PA14 genes were obtained from the MGH-ParaBioSys:NHLBI Program for Genomic Applications, Massachusetts General Hospital and Harvard Medical School, Boston, MA (http://pga.mgh.harvard.edu; accessed 10Nov2015) [45] made available by the *Pseudomonas Genome Database* (http://www.pseudomonas.com/; accessed 10Nov2015) [44].

^b^Gene expression within PA14 grown in whole blood from the three severely burned patients (Pt) was compared with expression when PA14 was grown in whole blood from a healthy volunteer.

^c^Genes found in operons: *pqsA/pqsB/pqsC; phzG1/phzF1/phzE1/phzD1/phzC1; phzG2/phzF2*.

**Fig 2:**
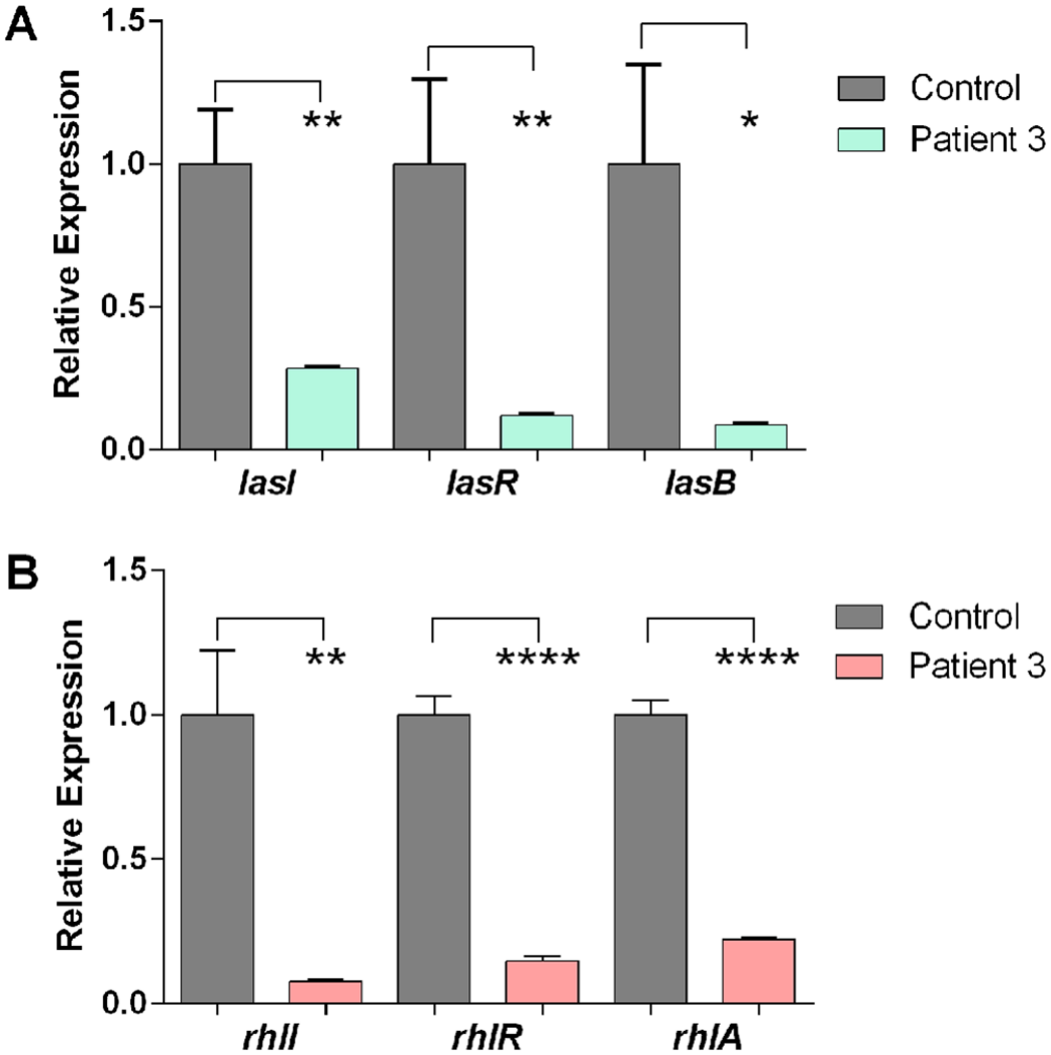
Growth of PA14 in whole blood from a severely burned patient significantly repressed expression of QS and QS-controlled genes. RNA extracted from PA14 grown in whole blood from Patient 3 or the healthy volunteer was examined in qRT-PCR as described in Methods. **A.** Level of expression of *lasI*, *lasR*, and *lasB*. **B.** Level of expression of *rhlI*, *rhlR*, and *rhlA*. Values in A and B represent the average of triplicate PCR experiments conducted on three independently obtained RNA preparations ±SEM; *P <0.05, **P<0.01, ****P <0.0001.

To confirm these results, we examined the expression of several of the QS and QS-controlled genes by qRT-PCR using RNA that was extracted from PA14 grown in blood from Patient 3 and the healthy volunteer. As in the RNA-seq analysis, the level of *lasR* and *lasB* (Fig 2A) and *rhlR* and *rhlA* (Fig 2B) expression was significantly reduced. However, unlike the results of the RNA-seq analysis, qRT-PCR revealed that the expression of both *lasI* and *rhlI* was also significantly reduced (four-fold and 14-fold, respectively) (Fig 2A and 2B). These results suggest that the growth of PA14 in blood from severely burned patients represses the expression of QS and QS-controlled genes.

The expression of the QS genes is complicated and involves several positive and negative regulators. Positive regulators include Vfr, GacA, and VqsR while negative regulators include MvaT, RsmA, QscR, RpoS, RsaL, and RpoN [47–52]. However, the expression of genes for the negative regulators *rsmA*, *rsaL*, and *mvaT* was reduced significantly in our RNA-seq analysis (Table 2), which rules out the possibility that the observed regulation of QS occurs through RsmA, RsaL, or MvaT. Conversely, expression of the positive regulatory gene *PA14_30580* (*vqsR* ortholog) was also significantly reduced (Table 2). Whether regulation of the QS and QS-controlled genes occurs through *PA14_30580* is yet to be determined.

These results suggest that the QS systems may not be essential for the survival of *P*. *aeruginosa* in blood during systemic infection (Table 2 and Fig 2). Using the murine model of thermal injury, we previously showed that compared with that of their parent strain, the *in vivo* virulence of *P*. *aeruginosa* mutants defective in the *lasI*, *rhlI*, or *lasI/rhlI* was significantly reduced [28]. Although the number of microorganisms within the injured/infected skin was basically the same, the number of microorganisms recovered from the internal organs of mice infected with the QS-mutants was significantly reduced, [28]. Based on these findings, we suggested that the QS system is important in the translocation of *P*. *aeruginosa* from the infected wound into the bloodstream [28]. Additional studies in the rat pressure ulcer model showed that the *P*. *aeruginosa* Las autoinducer 3OC12-HSL was produced within the *P*. *aeruginosa*-infected tissues at 3 and 7 d post infection [53]. In contrast to the above described studies, our current study is focused on the pathogenesis of *P*. *aeruginosa* during systemic infection. One possible explanation for the difference between this and the previous studies is that we utilized human blood in a system mimicking bloodstream infection rather than an animal model. Our microarray analysis using the murine model of thermal injury in which we compared gene expression in PA14 that was grown in whole blood obtained from either uninjured or thermally injured mice supported this possibility [34]. The growth of PA14 in blood from thermally injured mice altered the expression of more than 600 genes compared with growth in blood from uninjured mice [34]. However, we detected no change in the expression of the *las* and *rhl* genes [34]. This suggests that despite its closeness to *P*. *aeruginosa* infection of burned patients, the murine model of thermal injury does not parallel human infection with respect to alterations in the expression of different *P*. *aeruginosa* genes in blood.

This study is focused on the effect of burn-induced changes in blood on the expression of *P*. *aeruginosa* genes. However, it is possible that the growth of PA14 in blood from healthy volunteers would alter expression of many genes, including the QS genes, when compared with growth in the laboratory medium LB broth. We recently examined this possibility using RNA-seq experiments. Compared with LB broth, the growth of PA14 in whole blood from healthy volunteers did not change the expression of *lasR* or *rhlR* and reduced the expression of the *pqsA-E* operon by two- to four-fold while the Las- and Rhl-controlled genes *lasA* and *rhlB* were reduced three-fold and two-fold, respectively, and *plcB* expression was unchanged (Table 3). Therefore, considering the effect of growth in LB broth, whole blood from healthy volunteers, and whole blood from severely burned patients, reduction in the expression of the *las* and *rhl* genes may occur in two stages, an initial reduction produced by the growth in blood from the healthy volunteer and an additional reduction caused by the burn-induced changes in blood.

**Table 3:** Comparison of QS gene expression in healthy human blood to that in LB broth.

Gene	Protein product^a^	Healthy volunteer: LB broth^b^	Average of Burn Pts: Healthy volunteer^c^
*lasR*	LasR	1	-2
*lasA*	LasA	-3	-77
*lasB*	LasB	1	-4
*rhlR*	RhlR	1	-4
*rhlA*	RhlA	1	-12
*rhlB*	RhlB	-2	-11
*plcB*	PlcB	1	-3
*pqsE*	PqsE	-3	1
*pqsD*	PqsD	-3	1
*pqsC*	PqsC	-4	-2
*pqsB*	PqsB	-3	-2
*pqsA*	PqsA	1	-2
*phzH*	Potential phenazine-modifying enzyme	-2	4
*phzS*	Hypothetical protein	-23	4
*phzG1*	PhzG	-12	6
*phzF1*	PhzF	-12	4
*phzE1*	PhzE	-12	3
*phzD1*	PhzD	-9	3
*phzC1*	PhzC	-4	3
*phzG2*	Pyridoxamine 5'-phosphate oxidase	-25	3
*phzF2*	Phenazine biosynthesis protein	-12	4
*rsaL*	RsaL	-2	-8
*mvaT*	MvaT, P16 subunit	1	-3
*rsmA*	Carbon storage regulator	-4	-2
*PA14_30580*	LuxR family transcriptional regulator	4	-3

^a^Product names for PA14 genes were obtained from the MGH-ParaBioSys:NHLBI Program for Genomic Applications, Massachusetts General Hospital and Harvard Medical School, Boston, MA (http://pga.mgh.harvard.edu; accessed 10Nov2015) [45] made available by the *Pseudomonas Genome Database* (http://www.pseudomonas.com/; accessed 10Nov2015) [44].

^b^Expression of genes within PA14 that was grown in LB broth (LB) was compared with the expression when PA14 was grown in whole blood from a healthy volunteer (HV).

^c^Average of gene expression within PA14 grown in whole blood from the three severely burned patients was compared with expression when PA14 was grown in whole blood from a healthy volunteer calculated from results shown in Table 2.

At this time, we do not know the exact changes within blood that alter the expression of different PA14 QS genes. The most likely component to analyze next is serum. We recently showed that serum influences the expression of different *P*. *aeruginosa* genes [54]. During the growth of *P*. *aeruginosa* in LB broth containing 10% serum from healthy volunteers, the expression of QS genes followed a unique pattern in which the genes were repressed at early stages of growth (4 h post-inoculation, OD_600_ 1.0–1.2) but enhanced at late stages of growth (16 h post-inoculation, OD_600_ 3.0–4.0) [54]. Due to the extensive RBC lysis in PA14 blood cultures after 10 h, we did not harvest PA14 cells at late stages of growth. Therefore, we do not know if growing PA14 in blood, whether from healthy volunteers or burn patients, to late stages of growth would alter the expression of the QS genes. It is possible that severe burn targets the same component of normal serum that reduces the expression of QS genes at early stages of growth. As a result and compared with the growth of PA14 in LB containing 10% normal serum, the growth of PA14 in LB broth containing 10% serum from severely burned patients would further reduce the expression QS genes. To examine this possibility, we plan to compare the expression of QS-genes in PA14 grown in LB broth, LB broth contesting 10% serum from healthy volunteers, and 10% serum from severely burned patients at early and late stages of growth.

We analyzed the blood of the three burn patients for levels of calcium, phosphorus, albumin, and hemoglobin (Table 4). The level of albumin in the blood from all three patients is lower than normal (Table 4). Thus, while the blood component responsible for the changes in expression of the QS and QS-controlled genes is yet to be determined, albumin is a potential candidate. In this regard, albumin would enhance the expression of different QS genes.

**Table 4:** Severe burn injury alters the level of blood constituents.

Blood constituent^a^	Normal reference range	Patient 1	Patient 2	Patient 3
Calcium	8.8–10.5 (mg/dL)	8.0	7.5	8.3
Hemoglobin	13.3–17.1 (g/dL)	18.8	13.3	13.5
Phosphorus	2.4–4.1 (mg/dL)	2.7	1.6	2.5
Albumin	3.5–5.2 (g/dL)	3.0	2.7	2.2
Glucose	65–115 (mg/dL)	149	160	107
Potassium	3.5–5.1 (mmol/L)	4.5	3.4	4.0
Sodium	136–145 (mmol/L)	136	146	130

^a^Blood samples for these analyses were collected concurrently with blood culture samples and processed by the Clinical Laboratory at University Medical Center, Lubbock, TX.

The use of blood as a growth medium prevented us from utilizing the colorimetric and turbidimetric assays to assess the production of QS-controlled factors. Thus, we were unable to conduct the elastin Congo red, staphylolytic, or pyocyanin assays to determine the level of LasB activity, LasA activity, and pyocyanin, respectively. Also, the presence of LasB-cross reactive materials in blood prevented us from analyzing the level of LasB protein produced by PA14 grown in blood using immunoblotting experiments. Blood also interfered with the autoinducer assays that we use to measure the level of 3O-C12HSL, C4-HSL, and PQS produced by *P*. *aeruginosa*.

### Growth in Whole Blood Affects the Expression of Genes Related to Iron Acquisition

**Fig 3:**
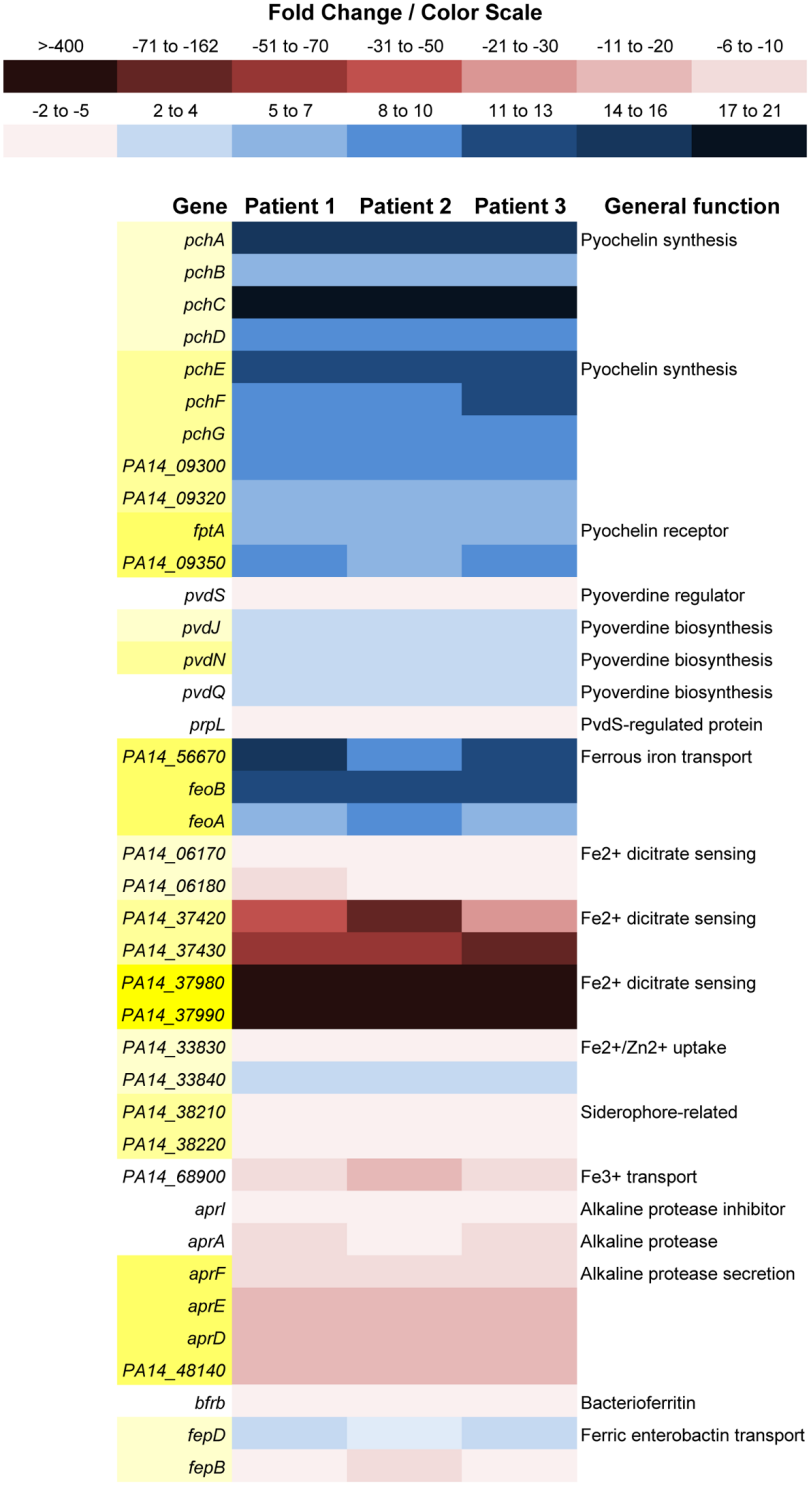
Color scale map of upregulated or downregulated iron acquisition genes. Expression of genes in PA14 grown in whole blood from the three severely burned patients was compared with expression of these genes when PA14 was grown in whole blood from a healthy volunteer (control). Upregulated genes are indicated in shades of blue while downregulated genes are indicated in shades of red. Genes within operons, whether all genes of the operon are differentially expressed or not, are indicated in shades of yellow on the left. Single genes are not shaded. Operon groupings and gene product functions for PA14 genes were obtained from the MGH-ParaBioSys:NHLBI Program for Genomic Applications, Massachusetts General Hospital and Harvard Medical School, Boston, MA (http://pga.mgh.harvard.edu; accessed 10Nov2015) [45] made available by the *Pseudomonas Genome Database* (http://www.pseudomonas.com/; accessed 10Nov2015) [44].

In *P*. *aeruginosa*, expression of genes for the two siderophore-mediated ferric iron-acquisition systems, pyochelin and pyoverdine, is repressed in the presence of iron [55]. In human blood, iron is present within the transport molecule transferrin in the ferric form; thus the bloodstream is essentially a reduced-iron environment. As expected, all the genes for pyochelin synthesis and transport were induced in PA14 grown in the blood of severely burned patients, except for *pchR*, a gene for transcriptional regulation. The pyoverdine siderophore is more efficient in acquiring Fe^3+^ than pyochelin [55]. In contrast, the expression of most (14 of 17) of the genes involved in pyoverdine synthesis was not altered significantly, with the exception of *pvdJ*, *pvdN*, and *pvdQ*, whose expression was enhanced by two- to four-fold (Fig 3). More importantly, expression of *pvdS*, the positive regulator of the pyoverdine genes, was reduced by approximately four-fold (Fig 3) [55]. Unlike the pyoverdine genes, and similar to *pvdS*, the PvdS-regulated gene *prpL*, which encodes a protease unrelated to iron acquisition [56], was downregulated by two-fold (Fig 3). While genes for ferrous iron transport (*feo* operon) were upregulated five- to 16-fold, two operons containing genes encoding ferrous dicitrate sensing proteins were downregulated by as much as 162-fold (Fig 3). Additionally, expression of the *aprA* gene encoding the AprA alkaline protease, which proteolytically cleaves transferrin [57], as well as genes involved in the secretion of AprA, was reduced by five- to 17-fold (Fig 3). Thus, it appears that the growth of PA14 in blood from severely burned patients differentially regulates the expression of iron acquisition genes, increasing the expression of some and reducing expression of others (Fig 3).

The reduction in *pvdS* expression is puzzling. Since pyoverdine is very efficient in transporting iron under iron-starvation conditions, we expected the *pvdS* and the pyoverdine genes to be significantly induced. We previously showed that serum enhances the expression of numerous iron-regulated genes including *pvdS* in the *P*. *aeruginosa* strain PAO1 [58]. Further analyses revealed that serum albumin enhances the expression of these genes [58]. To explore this phenomenon further, we first confirmed the results of the RNA-seq experiments using qRT-PCR. Compared with blood from healthy volunteers, the growth of PA14 in blood from severely burned patients reduced *pvdS* expression by approximately 2.5-fold (data not shown). Similar to PAO1, *pvdS* expression in PA14 is stringently regulated by iron. The gene was expressed when PA14 was grown in the iron-deficient medium TSB-DC and was significantly repressed upon the addition of iron (S4 Fig). Surprisingly, compared with its growth in TSB-DC, the growth of PA14 in blood from a healthy volunteer caused a significant reduction in *pvdS* expression (S4 Fig) suggesting the presence of certain factor(s) within blood or serum that represses pvdS expression. Similar to the observed effect on the QS genes, burn-induced changes within blood may exaggerate further the reduction in *pvdS* expression. To determine if the regulation of pyoverdine genes by *pvdS* in PA14 is different from that in PAO1, we constructed a *pvdS* isogenic mutant of PA14 using the previously described gene replacement technique [59]. Compared with its PA14 parent strain, PA14::*pvdS* mutant was defective in pyoverdine production suggesting that in PA14, the expression of the pyoverdine genes depends on the presence of a functional *pvdS* (data not shown).

One possible explanation for these findings is that PA14 is capable of producing pyochelin at the expense of pyoverdine. Pyoverdine affinity for iron is high while that of pyochelin is low [60]. Dumas *et al*. [61] recently suggested the presence of a molecular mechanism through which *P*. *aeruginosa* phenotypically switches from producing pyoverdine under extreme iron limitation to producing pyochelin under moderate iron limitation. In the presence of low amounts of supplemented iron, pyoverdine synthesis is strongly repressed through the Fur-related negative feedback system [61]. However, pyochelin continues to be synthesized at relatively high levels over an extended period [61]. Therefore, if a severe burn causes a limited increase in the level of iron in blood, such increase may repress the expression of the pyoverdine genes but increase the expression of pyochelin genes (Fig 3). Dumas *et al*. [61] also suggested that the switch is influenced by the environmental pH and the level of available nutrients. Shifting the pH from 7 to 8 or 9 decreased pyoverdine synthesis but increased pyochelin synthesis [61]. Additionally, since *P*. *aeruginosa* requires 3.6 more amino acids and nucleotides to transcribe and translate the pyoverdine genes than it needs to transcribe and translate the pyochelin genes, it would be more economical for *P*. *aeruginosa* to produce pyochelin than pyoverdine [61]. Utilizing *P*. *aeruginosa* strains PAO1 and PA14, we are currently conducting a detailed comparative analysis between *P*. *aeruginosa* cultures in blood from healthy volunteers and those in blood from severely burned patients with respect to the level of iron, the pH, and the amount of nucleotides and amino acids.

### Growth in Whole Blood Affects the Expression of T3SS Genes

**Fig 4:**
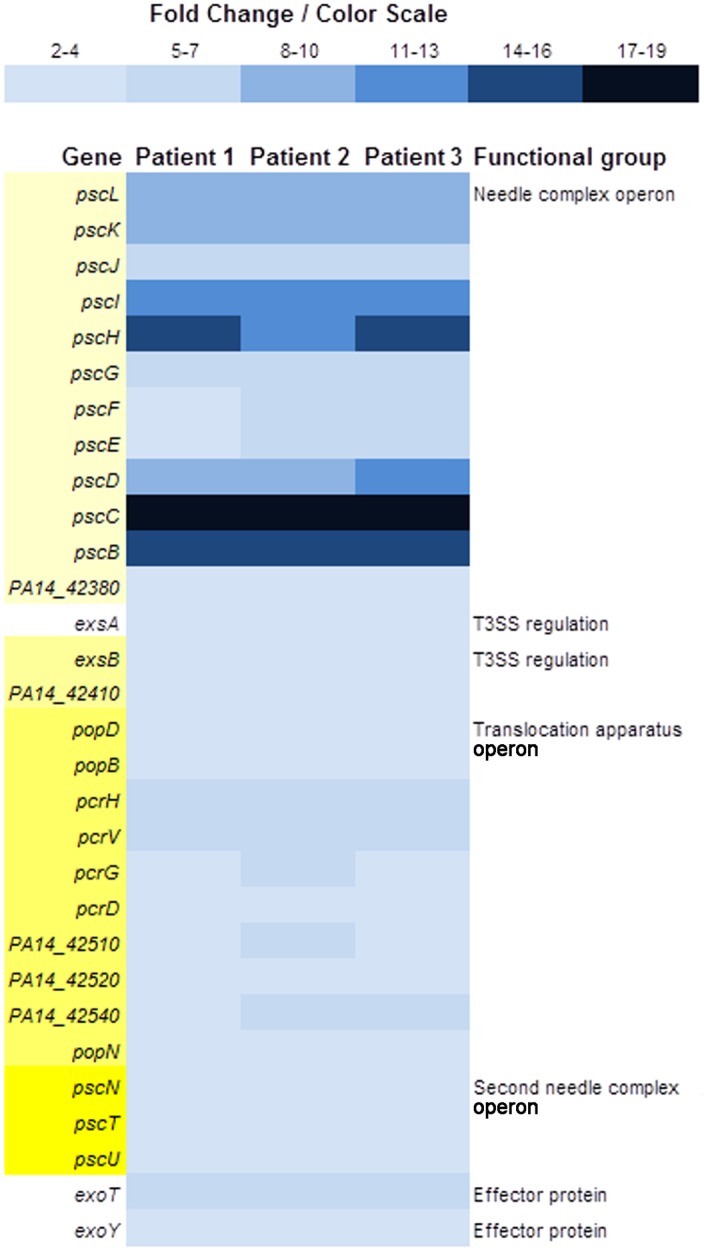
Color scale map of the T3SS genes that are either upregulated. Expression of genes in PA14 grown in whole blood from the three severely burned patients was compared with expression of these genes when PA14 was grown in whole blood from the a healthy volunteer. Upregulated genes are indicated in shades of blue. Genes within operons, whether all genes of the operon are differentially expressed or not, are indicated in shades of yellow on the left. Single genes are not shaded. Operon groupings and functions for PA14 genes were obtained from the MGH-ParaBioSys:NHLBI Program for Genomic Applications, Massachusetts General Hospital and Harvard Medical School, Boston, MA (http://pga.mgh.harvard.edu; accessed 10Nov2015) [45] made available by the *Pseudomonas Genome Database* (http://www.pseudomonas.com/; accessed 10Nov2015) [44].

**Fig 5:**
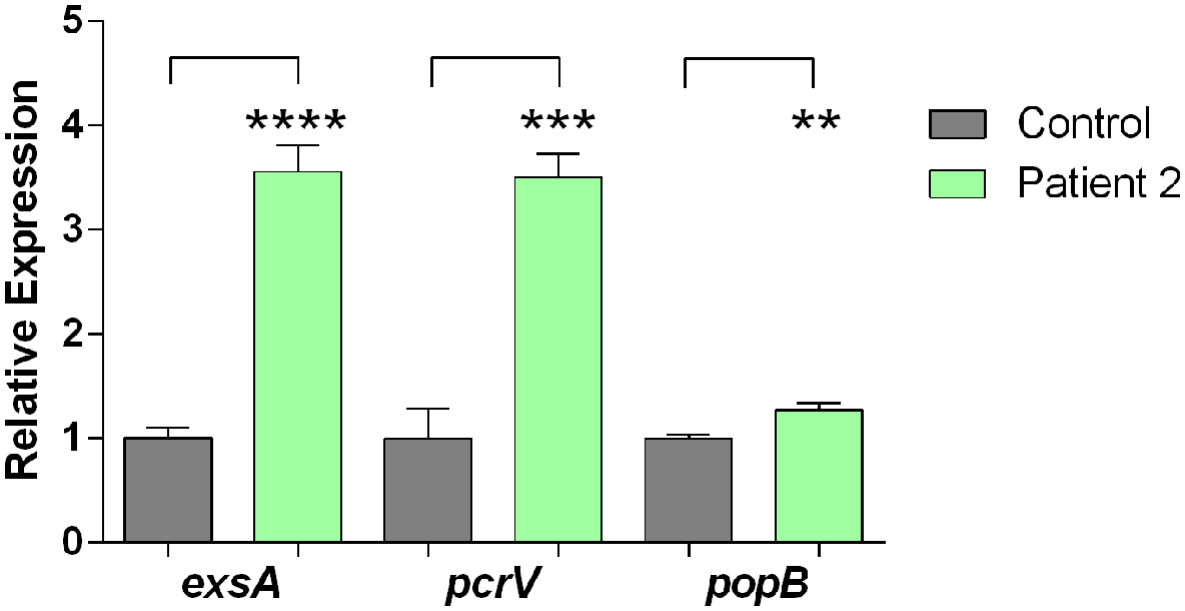
Growth of PA14 in whole blood from a severely burned patient significantly enhanced expression of the T3SS genes *exsA*, *pcrV*, and *popB*. RNA extracted from PA14 grown in whole blood from Patient 2 or the healthy volunteer was examined in qRT-PCR as described in Methods. Values represent the average of triplicate PCR experiments conducted on three independently obtained RNA preparations ±SEM; **P<0.01, *** *P* <0.001, ****P <0.0001.

Among the 37 genes comprising the five major T3SS operons and scattered genes that code for the needle complex, translocation apparatus, chaperones, effector proteins, and regulators in PAO1 [62], the expression of 30 of these genes, which are found in four operons in PA14 plus scattered genes, was significantly enhanced (Fig 4) (http://pga.mgh.harvard.edu and http://www.pseudomonas.com/; accessed 10Nov2015) [44,45]. We also validated these findings using qRT-PCR. Compared with blood from a healthy volunteer, the growth of PA14 in blood from Patient 2 significantly enhanced the expression of the main regulatory gene, *exsA*, and the genes encoding two of the three T3SS translocation apparatus, *pcrV*, and *popB* (Fig 5).

The expression of T3SS genes in *P*. *aeruginosa* is stringently regulated by the level of Ca^2+^ in the growth medium [63]. At a concentration of 2 mM (8 mg/dL), calcium represses the expression of T3SS genes [64]. The physiological range of Ca^2+^ in human blood is 8.8–10.5 mg/dL (Table 4), which is sufficient to repress the expression of T3SS genes *in vitro*. Thermal injury often reduces the level of calcium in blood, which could induce the expression of the T3SS genes. Analysis of the calcium levels in the blood of the three patients confirmed this reduction in calcium. However, only in Patient 2 was the blood calcium level sufficiently low at 7.5 mg/dL (Table 4) to allow expression of the T3SS genes. The levels of calcium in the blood from Patient 1 and Patient 3, while below physiologic normal at 8.0 and 8.3 mg/dL (Table 4), respectively, should have been sufficient to repress the expression of T3SS genes. Yet expression of the T3SS genes was enhanced at similar levels in all three patients (Fig 4). Thus, the observed effect is likely to be due to calcium-independent, injury-induced alterations in blood.

Other potential blood/serum signals that may affect the T3SS are the low-affinity, high-capacity Ca-binding proteins, such as albumin [65]. Initial studies showed that these proteins enhance the secretion of the T3SS effectors [65]. Kim *et al*. [65] previously showed that the secretion of T3SS effector molecules requires certain serum factors. In the absence of these factors, basal levels of T3SS effector molecules accumulate intracellularly [65]. Either the same or additional serum factors may enhance the expression of T3SS genes. Our recent RNA-seq analyses support this possibility and suggest that albumin enhances the expression of different T3SS genes. Compared with LB broth, the growth of PA14 in LB broth containing 5% albumin, which is comparable to the level of albumin in the blood of healthy individuals, increased the expression of several T3SS genes, such as *exoT*, *exoY*, *escB*, *popB*, *popB*, *pscB*, *pscD*, and *pscN* by two-fold (Table 5).

**Table 5:** Comparison of T3SS gene expression in LB broth with 5% albumin to LB Broth.

Gene	Protein^a^	LB broth/5% albumin:LB broth^b^	Average of Burn Pts: Healthy volunteer^c^
*exoT*	ExoT, effector protein	2	5
*exoY*	ExoY, effector protein	2	2
*exsB*	ExsB, T3SS regulation	2	3
*pcrD*	PcrD, translocation apparatus operon	1	4
*pcrG*	PcrG, translocation apparatus operon	3	4
*pcrH*	PcrH, translocation apparatus operon	2	6
*pcrV*	PcrV, translocation apparatus operon	2	6
*popB*	PopB, translocation apparatus operon	2	4
*popD*	PopD, translocation apparatus operon	2	4
*popN*	PopN, translocation apparatus operon	2	3
*pscB*	PscB, needle complex operon	2	15
*pscC*	PscC, needle complex operon	2	17
*pscD*	PscD, needle complex operon	2	10
*pscE*	PscE, needle complex operon	2	5
*pscF*	PscF, needle complex operon	2	5
*pscG*	PscG, needle complex operon	1	6
*pscH*	PscH, needle complex operon	1	14
*pscJ*	PscJ, needle complex operon	2	7
*pscN*	PscN, second needle complex operon	2	3

^a^Product names for PA14 genes were obtained from the MGH-ParaBioSys:NHLBI Program for Genomic Applications, Massachusetts General Hospital and Harvard Medical School, Boston, MA (http://pga.mgh.harvard.edu; accessed 10Nov2015) [45] made available by the *Pseudomonas Genome Database* (http://www.pseudomonas.com/; accessed 10Nov2015) [44].

^b^Expression of genes within PA14 that was grown in in LB broth with 5% albumin was compared with expression when PA14 was grown in LB broth.

^c^Average of gene expression within PA14 grown in whole blood from the three severely burned patients was compared with expression when PA14 was grown in whole blood from a healthy volunteer calculated from results used to derive Fig 4.

Similar to its effect on the expression of QS genes, blood may affect the T3SS genes in two stages; stage one produced during the growth of *P*. *aeruginosa* in normal blood and stage two produced by the severe burn-induced changes in blood. We recently showed that compared with the growth in LB broth, the growth of *P*. *aeruginosa* in blood from healthy volunteers significantly enhanced the expression of genes within the T3SS *pscA-L* operon from two- to four-fold. This expression is further enhanced by the growth of PA14 in blood from severely burned patients (Figs 4 and 5). However, while albumin may contribute to the first stage of the effect of blood on T3SS (the growth of PA14 in blood from healthy volunteers), it is less likely to contribute to the effect during second stage (the growth in blood from severely burned patient). The level of albumin in the blood of all three patients was lower than the normal reference range (Table 4). Therefore, burn-induced changes in blood may enhance the expression of T3SS genes through a mechanism that is both Ca^2+^- and albumin-independent. One possible approach to examine this possibility is to deplete albumin from serum obtained from either healthy volunteers or severely burned patients. The effect of PA14 growth in LB broth supplemented with this depleted serum on the expression of different T3SS genes would then be compared. We previously utilized such approach to examine the influence of serum albumin on the expression of iron-regulated genes in the *P*. *aeruginosa* strain PAO1 [58].

### Growth in Whole Blood Affects the Expression of Numerous Transport Genes

The growth of PA14 in blood from severely burned patients influenced the expression of genes that code for several different types of transport systems including heme transport (S1 Table), metal ion transport systems besides those for iron (S2 Table), numerous ABC-transporters for other substrates (S3 and S4 Tables), TonB as well as TonB-dependent transporters (S5 Table), phosphate transport (Table 6), and phosphonate transport (S6 Table).

**Table 6:** Differentially expressed genes of the phosphate regulon (Pho).

Gene	Product name	Pt 1	Pt 2	Pt 3
	Functional classification(s) // gene ontology terms			
	Pathways // Functional prediction(s)^a^			
*phoU*	**Phosphate uptake regulatory protein PhoU**	-19^b^	-21	-24
	Transcriptional regulators; transport of small molecules; membrane proteins // Negative-regulation of positive chemotaxis			
	// Phosphate transport system regulatory protein PhoU			
*pstB*^c^	**Phosphate transporter ATP-binding protein**	-17	-23	-21
	Transport of small molecules // Phosphate ion transmembrane; membrane; inorganic phosphate transmembrane transport activity; phosphate ion trans-membrane ATPase activity; ATP binding			
	ABC transporters // Phosphate transport system permease protein 1; ABC transporter, phosphate import, PstB; AAA+ ATPase domain			
*pstA*	**Phosphate ABC transporter permease**	-34	-34	-36
	Transport of small molecules // Phosphate ion transmembrane transport; integral component of membrane; inorganic phosphate transmembrane transport activity			
	ABC transporters // Phosphate transport system permease protein PstA			
*pstC*	**Membrane protein component of ABC phosphate transporter**	-17	-15	-18
	Transport of small molecules // Transport; membrane; protein binding			
	ABC transporters // ABC transporter integral membrane type-1 domain profile; binding-protein-dependent transport system inner membrane component			
*oprO*	**Pyrophosphate-specific outer membrane porin OprO precursor**	-45	-48	-57
	Transport of small molecules; no GO terms listed			
	// Phosphate-selective porin O and P			
*oprP*	**Pyrophosphate-specific outer membrane porin OprP precursor**	-14	-5	-4
	Transport of small molecules // no GO terms listed			
	// Phosphate-selective porin O and P			
*phoB*	**Two-component response regulator PhoB**	-9	-10	-13
	Transcriptional regulators; two-component regulatory systems // Phosphate ion transport; bacterial-type flagellum-dependent swarming motility; positive-regulation of cell response to phosphate starvation; phosphorelay signal transduction system; regulation of transcription, DNA-templated; phosphorelay response regulator activity, DNA binding			
	Two-component system // Signal transduction response regulator, phosphate regulon transcriptional regulatory protein PhoB; signal transduction response regulator, receiver domain; winged helix-turn-helix DNA-binding domain			
*phoR*	**Two-component sensor PhoR**	-5	-5	-6
	Two-component regulatory systems // Regulation of transcription, DNA-templated; phosphorylation; phosphorelay signal transduction system; integral component of membrane; protein histidine kinase activity; phosphorelay sensor kinase activity; transferase activity (phosphorus-containing groups); signal transducer activity;			
	Two-component systems // Signal transduction histidine kinase EnvZ-like, dimerisation/phosphoacceptor domain; Signal transduction histidine kinase, phosphate regulon sensor PhoR			
*phoQ*^c^	**Two-component sensor PhoQ**	-3	-5	-5
	Two-component regulatory systems // Signal transduction; phosphorylation; integral component of membrane; signal transducer activity; transferase activity (phosphorus-containing groups); phosphorelay sensor kinase activity			
	Two-component systems // Signal transduction histidine kinase EnvZ-like, dimerisation/phosphoacceptor domain; Signal transduction histidine kinase, homodimeric domain			
*phoP*	**Two-component response regulator PhoP**	-5	-7	-7
	Transcriptional regulators; two-component regulatory systems // Regulation of transcription, DNA-templated; positive regulation of phospholipid biosynthetic process; phosphorelay signal transduction system; DNA binding			
	Two-component system // Signal transduction response regulator, receiver domain; winged helix-turn-helix DNA-binding domain			
*oprH*	**PhoP/Q and low Mg2+ inducible outer membrane protein**	-12	-13	-15
	Adaptation, protection; transport of small molecules; membrane proteins // integral component of membrane, cell outer membrane; lipopolysaccharide binding			
	// Outer membrane protein beta-barrel domain			
*PA14_55770*	**Phosphate transporter**	2	2	2
	Transport of small molecules; membrane proteins // Phosphate ion transport; membrane; inorganic phosphate transmembrane transport activity			
	// Phosphate transporter family			

^a^Product names, functional classification(s), gene ontology terms, pathways, and functional predictions for PA14 genes were obtained from the MGH-ParaBioSys:NHLBI Program for Genomic Applications, Massachusetts General Hospital and Harvard Medical School, Boston, MA (http://pga.mgh.harvard.edu; accessed 10Nov2015) [45] made available by the *Pseudomonas Genome Database* (http://www.pseudomonas.com/; accessed 10Nov2015) [44].

^b^Gene expression within PA14 grown in whole blood from the three severely burned patients (Pt) was compared with expression when PA14 was grown in whole blood from a healthy volunteer.

^c^Genes found in operons: *pstB/pstA/pstC* (and related protein *phoU*); *phoQ/phoP/oprH*.

With respect to the heme transport and utilization systems, the expression of at least 19 genes was affected (S1 Table). The heme acquisition gene *hasAP* and the heme uptake gene *hasR* [66,67], plus two related transport genes (*hasD* and *PA14_20050*) were downregulated in PA14 grown in the blood of all three patients, whereas genes encoding the heme uptake receptor PhuR and the HmuV subunit of the hemin importer were upregulated (S1 Table).

Besides having an effect on the iron transport systems such as pyochelin, Fe2+ dicitrate sensing, and others (Fig 3), transport systems for other metal ions were also up- or downregulated in PA14 grown in the blood from all three burn patients (S2 Table). Some genes for copper transport were upregulated, while others were downregulated (S2 table). Additionally, the *czcCczcB* operon that encodes proteins for efflux of cobalt, zinc, and cadmium was downregulated by two- to nine-fold while genes of an operon encoding proteins for manganese and zinc transport were upregulated four- to 11-fold (S2 Table).

Since many of the proteins involved in transport are part of well-defined ABC-transport systems, we analyzed the RNA-seq data for the level of expression of genes encoding additional ABC-transporters. The growth of PA14 in blood from all patients significantly enhanced the expression of 29 ABC-transport genes from two- to 21-fold (S3 Table). These ABC transporter systems are predicted to be involved with cell metabolism (lipoprotein release, protein metabolism, and carbohydrate transport), iron transport, and multidrug resistance (S3 Table). However, the expression of 78 other ABC-transport genes was significantly reduced in all patients (S4 Table). Another group of proteins that play a major role in transport are dependent on TonB. The gene for the multifunctional TonB protein and 23 genes coding for individual TonB-dependent receptors or operons associated with TonB-dependent receptors were downregulated significantly (S5 Table). Some genes were downregulated by 200- to 700-fold (S5 Table).

With the exception of *PA14_55770* which codes for a putative phosphate transporter, the expression of genes in the phosphate regulon (Pho) was repressed in PA14 grown in blood from severely burned patients (Table 6). The expression of the phosphate transporter permease gene *ptsA* was reduced by more than 30-fold while that of the phosphate transporter ATP-binding protein *ptsB* and the membrane component of the transporter *ptsC* was reduced by 15- to 23-fold (Table 6) [68]. Similarly, the expression of the *phoB/phoR* two-component regulatory system was reduced by at least five-fold (Table 6) [68]. This repression was not induced by a change in the level of phosphate ion within the blood of each patient (Table 4).

PhoR is an inner membrane histidine kinase while PhoB is response regulator DNA binding protein [69]. Phosphate levels higher than 4 μM (about 38 μg/dL) repress the operon [70]. Phosphate levels within the blood of healthy individuals (2400–4100 μg/dL) and severely burned patients (1600–2700 μg/dL) are significantly higher than those required to repress the Pho regulon (Table 4). As such, one would expect the different genes of the Pho regulon to be completely repressed under both conditions. However, *phoU*, the *pstBAC* operon, and the *phoQphoPoprH* operon were enhanced from two- to 18-fold when PA14 was grown in the blood of healthy volunteers and their expression is repressed when the bacteria is grown in blood from severely burned patients (Table 6) suggesting that factors within blood other than phosphate enhance the expression of the Pho regulon.

Environmental conditions, other than phosphate, are known to regulate the Pho regulon through the cross regulatory mechanisms [69]. If the Pho regulon is positively regulated by a blood/serum factor, severe burn would then either interfere with the function of this factor or reduce its level, thereby significantly reducing the expression of different genes of the Pho regulon. The *phoR/phoB* system controls the synthesis of the stress response molecule polyphosphate (poly P) which is involved in different bacterial functions including stress, growth, development, iron-chelation, and capsule synthesis [69,71–73]. Rashid *et al*. [73] previously showed that *P*. *aeruginosa* mutants defective in poly P kinase gene (*ppk*) were deficient in flagellum-related swimming motility and pilin-related twitching motility. Additionally, the mutant produced reduced levels of the QS-controlled virulence factors, LasB and RhlA [74]. One possible scenario to explain the effect of severe burn on the expression of the *phoR/phoB* genes as well as the QS genes is that in response to changes within the blood of severely burned patients, *phoB/phoR* represses the expression of the QS-genes through *ppk*. However, our results do not support this possibility as we detected no significant burn-induced changes in *ppk* expression. Besides, the growth in blood from severely burned patients increased the expression of numerous flagellum synthesis genes but decreased expression of pilin and fimbrial synthesis genes (S7 Table).

With respect to the phosphonate transport and metabolism systems, the expression of 15 genes in the two *phn* operons was repressed in PA14 grown in blood from all patients (S6 Table). The level of this repression varied considerably; for example, while some genes such as *phnP*, *phnW*, and *phnX* were repressed only two- to six-fold, other genes such as *phnC*, *phnD*, and *phnE* were repressed 196- to 996-fold (S6 Table).

## Conclusion

This study represents a unique approach to examine the effect of burn-induced changes on the virulence of *P*. *aeruginosa*. By growing *P*. *aeruginosa* in whole blood, we bypassed the problems associated with growing the bacteria *in vitro* in a laboratory medium. Additionally, by growing PA14 in blood from severely burned patients and healthy volunteers, we specifically addressed the effect of burn-induced changes in blood on *P*. *aeruginosa* virulence. As we demonstrated in this study, the growth of PA14 in blood from severely burned patients altered the expression of almost 2600 genes (PRNJA287707). Many of these genes are considered virulence or virulence-related genes including genes for QS and phenazine biosynthesis (Table 2), type 3 secretion (Fig 4), motility (S7 Table), heme transport and utilization (S1 Table), and alginate synthesis, porins and outer membrane proteins, and multidrug efflux proteins (SRA PRNJA287707). Similarly, genes encoding the iron (Fig 3), phosphate (Table 6) and phosphonate (S6 Table) transport systems that are critical for the survival of *P*. *aeruginosa* were differentially regulated.

The current analyses clearly show that the pathogenesis of *P*. *aeruginosa* during systemic infection of severely burned patients is an extremely complicated process and involves differential expression of numerous virulence and virulence-associated genes. Specific sets of virulence and virulence-related genes were upregulated while others were downregulated. The next critical question to address is the nature of the severe burn-induced changes in blood that influence the expression of these genes. In this study, we eliminated some obvious causes such as burn-induced variations in certain ions. Although the nature of the factor(s) is yet to be determined, it is possible that the variations in the expression of different genes may occur in response to one specific change or multiple changes in blood composition. We are currently attempting to decipher these changes. It is also critical to determine if these changes in *P*. *aeruginosa* are strain specific. In this study, we utilized *P*. *aeruginosa* strain PA14 which was originally isolated from an infected wound. We plan to determine the effect on the other commonly used laboratory strain PAO1, which was also isolated from an infected wound. We also plan to determine if the growth of recently obtained *P*. *aeruginosa* wound isolates under the same conditions would bring about similar variations in the expression of virulence, virulence-associated, and transport genes.

## Materials and Methods

### Bacterial Strain, Media, and Growth Conditions

PA14, a prototrophic strain of *P*. *aeruginosa* originally isolated from a wound infection [35], was used in all experiments. The strain was routinely grown and maintained at 37°C in Luria Bertani (LB) broth prior to subculture into whole blood or other media.

### Ethics Statement

This study was approved by the Texas Tech University Health Sciences Center Institutional Review Board. Written consent was obtained upon the patient’s admission to the Timothy A. Harnar Burn Unit at University Medical Center, Lubbock, TX, by a staff member of the Clinical Research Institute (CRI) at TTUHSC in compliance with ethical practices. If the patient was unable to provide written consent, written consent was obtained from designated next of kin by a CRI staff member according to the protocol approved by the IRB. Written consent was obtained from healthy volunteers by an individual with appropriate training mandated by the IRB under this IRB-approved protocol. No children were involved in this study. Venous blood was collected from 4 healthy volunteers and three severely burned patients. A total of 25 mL of whole blood was collected from each person. The blood was collected in three BD Vacutainer tubes (8.3 mL/tube) containing sodium polyanetholesulfonate (SPS) as an anticoagulant.

### The Closed Loop Flow through Model (CLFT)

This was based on the model previously described by Kealey *et al*. [33] to examine platelet deposition on arterial stents. We used three 2-ft loops of 16-mm platinum-cured silicone tubing with an inside diameter of 3.2 mm (Masterflex) to hold 7.5 mL of blood in each loop for each experiment (three independent experiments). Blood within the tubing was circulated by a peristaltic pump (Masterflex L/S) at a flow rate of 176 mL/min. The circulating blood was maintained at 37°C by immersing the tube in 37°C water bath (S1 Fig).

### Whole Blood Cultures

#### Preparation of the bacterial inoculum

PA14 was grown overnight at 37°C. A 1-mL aliquot of the overnight culture was pelleted, washed twice, and resuspended in 1 mL of fresh LB broth. The 7.5 mL of blood in each loop of tubing was inoculated with the PA14 culture to produce an initial inoculum of 1–4 X 10^2^ colony forming units (CFU). We conducted several preliminary experiments using LB broth to adjust the dilution of PA14 that resulted in this initial inoculum. Once inoculated, the PA14 blood culture was maintained in the CLFT system at 37°C and the contents of each loop were harvested at 8 h post inoculation.

#### Removal of lymphocytes and red blood cells from the PA14 blood culture

We differentially centrifuged the lymphocytes using a commercially available protocol. Briefly, the blood culture was diluted 1:1 in phosphate buffered saline and layered onto lymphocyte separation medium (Lonza, Basel, Switzerland) in a ratio of 3:2. The tubes containing the diluted culture and the separation medium were centrifuged at 400 x *g* for 15 min. The top two layers containing white blood cells were discarded and the bottom layer containing the bacterial pellet and RBC was retained. This layer was transferred to erythrocyte lysis buffer (QIAGEN, Valencia, CA), gently mixed, and incubated on ice for 20 min. The mixture was then centrifuged at 7,700 x *g* and the supernatant containing lysed RBC was discarded. The process was repeated several times until the bacterial pellet was free from all RBC. The pellet was then resuspended in 3 mL of LB broth and 6 mL of RNA Protect (QIAGEN) and stored at -80°C.

As a control, and to eliminate the possibility that these manipulations may affect gene expression within the PA14 pellet, we grew PA14 in LB broth using the CLFT system as described above for blood cultures and subjected part of the pellet to the same above described manipulations to remove blood cells. We then compared the level of expression of several genes within the two parts of the pellet.

### RNA Isolation

Bacterial pellets were first lysed by the addition of lysozyme and proteinase K for 15 min at room temperature. RNA was extracted using the RNeasy Mini Kit (QIAGEN) according to the manufacturer’s recommendations and the RNA solution was digested with the RNase-free DNase Set (QIAGEN). The DNase was completely removed from the RNA using the RNA cleanup protocol (QIAGEN) with the exception that on-column DNase digestion was applied to eliminate any remaining traces of genomic DNA. The quality of the RNA was further checked in PCR reactions using genomic DNA as a template (positive control) and purified RNA as a template (negative control). The RNA was then aliquoted for use in construction of the RNA-seq libraries and qRT-PCR. The purified RNA was quantified by NanoDropH spectrophotometer (Nanodrop Technologies, Wilmington, DE) and the integrity of the RNA was assessed using RNA Nano Chip on an Agilent 2100 Bioanalyzer (Agilent, Palo Alto, CA).

### Construction of the RNA-Seq Libraries

RNA samples with 1.8–2.2 ratio of absorbance 260/280 nm were kept for further analysis. The integrity of RNA was further measured on a TapeStation 2200 (Agilent), following the manufacturer’s instructions. Only samples with RNA Integration Number (RIN^e^) greater than 8.0 were used for cDNA library preparation. rRNA was removed from total RNA with Ribo-Zero rRNA removal kit for bacteria (Epicentre Biotechnologies, Madison, WI). Enriched mRNA samples were run on the TapeStation 2200 to confirm reduction of 16S and 23S rRNA. Six (three burn patients and three healthy volunteers) RNA-seq libraries were constructed from ~1000 ng of rRNA-depleted RNA using the TruSeq RNA library preparation kit following the manufacturer’s protocol (Illumina, San Diego, CA). RNA was fragmented and primed for cDNA synthesis at 94°C in an attempt to obtain a median insert size of 180 bp fragment. The fragmented RNA templates were primed with random hexamers and first strand was synthesized by four cycles of: 25°C for 10 min, 42°C for 50 min, and 70°C for 15 min. Following second strand synthesis (16°C for 1 h), end repair was performed to generate blunt ends followed by adenlyation of the 3′ blunt ended ds cDNAs to allow for subsequent ligation of multiple indexing adaptors and hybridization onto the flow cell. cDNA fragments were amplified and enriched using 15 cycles of PCR. The libraries were quantified using a Qubit^®^ 2.0 fluorometer (Life Technologies, Carlsbad, CA), and the quality was analyzed with the TapeStation 2200 using the DIK tape for validating the purity and estimating the insert size.

### Sequencing Using Illumina MiSeq Platform

Validated and indexed cDNA libraries were denatured with NaOH and normalized to 10 nM concentrations. Each of the 10 nM cDNA libraries were diluted to 4 nM with hybridization buffer and multiplexed. A final concentration of 5.4 pM was loaded onto the MiSeq reagent cartridge (MiSeq Reagent Kit v2 300cycles, Illumina). The multiplexed cDNA library was sequenced using a MiSeq Sequencer (Illumina) using V2 chemistry. Paired end sequencing was performed to obtain 150 bp reads using a 300 cycle reagent cartridge.

### Analysis of Differential Gene Expression among the PA14 Transcriptomes

Using UCBPP-PA14 as a reference genome [44,45], the differentially expressed genes in the individual samples were identified and analyzed using the QSeq module of Lasergene genomic suite 11.2 (DNA STAR). Reads assigned per kilobase of target per million mapped reads (RPKM) normalization was used before quantification of gene expression by QSeq. Student’s two-tailed unpaired *t*-test with Benjamini Hochberg FDR correction was used to compare the means of gene expression values for three individual replicates for a given gene. The genes were identified as differentially expressed with ≥ two-fold changes if they had the false discovery rate method for multiple testing correction *p*-value of ≤ 0.01 [75].

Data for each of the six samples were deposited in the BioProject at NCBI under PRJNA287707. Individual accession numbers for the three burn patients are SRS973482, SRS974920, and SRS974918; accession numbers for the three healthy volunteers are SRS973461, SRS973478, and SRS973513 (http://www.ncbi.nlm.nih.gov/bioproject/ or http://www.ncbi.nlm.nih.gov/biosample?LinkName=bioproject_biosample_all&from_uid=287707 for individual data sets [accessed 03Dec2015]).

### Reverse Transcription Quantitative PCR (qRT-PCR)

The integrity of RNA purified as described above was assessed using RNA Nano Chip on an Agilent 2100 Bioanalyzer (Agilent). cDNA was synthesized from the extracted RNA using the QuantiTect Reverse Transcription Kit (QIAGEN). Briefly, a 200-ng aliquot of cDNA was mixed with SYBR Green PCR Master Mix (Life Technologies) and 250 nM of specific primer. Amplification and detection of the product was carried out using the StepOne Plus real-time PCR system (Life Technologies). Three independent biological replicates for RNA extraction were used for each experiment. In addition, each PCR reaction was set up in triplicate. The quantity of cDNA in different samples was normalized using 30S ribosomal RNA (*rpsL*) as an internal standard. Gene expression analysis was performed using StepOne Plus software version 2.2.2 (Life Technologies). Positive control samples containing genomic DNA as a template and negative control samples containing RNA as a template were included in each experiment.

## Supporting Information

S1 Fig Diagram of the closed loop flow through system used to grow *P*. *aeruginosa* strain PA14 in whole blood. The peristaltic pump was set at a flow rate of 176 mL/min, the water bath maintained at 37°C, and 2-ft sections of 16-mm platinum-cured silicone tubing (one loop is shown, but up to 4 loops can be used at one time) were used to hold the blood. The non-reducing connecter minimizes trauma to the red blood cells.
(TIF)

S2 Fig Gene Ontology (GO) of the differentially expressed PA14 genes within the biological processes category. Genes whose expression was differentially regulated when the bacteria were grown in whole blood from the patients compared to the healthy volunteer were analyzed using PANTHER GO-Slim Biological Proccess analysis [42,43]. A total of 6875 GO terms were assigned to the 2348 mapped genes, with 1407 of these being unclassified. The remaining 5468 terms represented metabolic and catabolic processes– 3336 **(A)**, transport and localization– 808 **(B)**, biological regulation, biosynthetic processes, responses to stimuli, and cellular component organization or biogenesis– 694 **(C)**, and cellular processes– 630 **(D)**.
(TIF)

S3 Fig Gene Ontology (GO) terms assigned of the differentially expressed PA14 genes within the cellular components category. Genes whose expression was differentially regulated when the bacteria were grown in whole blood from the patients compared to the healthy volunteer were analyzed using PANTHER GO-Slim Cellular Component analysis [42,43]. A total of 2740 GO terms were assigned to the 2348 mapped genes, with 2139 of these being unclassified. The remaining 601 terms reflect all parts of the cell.
(TIF)

S4 Fig *pvdS* expression is downregulated in whole blood from healthy volunteers. Overnight culture of PA14 was inoculated into the iron-deficient medium TSB-DC, TSB-DC with added iron (as FeCl_3_) to suppress expression of iron-regulated genes (TSB-DC/Fe), and whole blood. Cultures were grown for 8 h at 37°C and cells were harvested to obtain RNA. Purified RNA was used to produce cDNA for qRT-PCR. Values represent the average of triplicate PCR experiments conducted on three independently obtained RNA preparations ±SEM; *** *P* <0.001.
(TIF)

S1 Table Differentially expressed heme transport and utilization genes. Gene expression within PA14 grown in whole blood from the three severely burned patients was compared with expression when PA14 was grown in whole blood from a healthy volunteer. Product names, functional classification(s), gene ontology terms, pathways, and functional predictions for PA14 genes were obtained from the MGH-ParaBioSys:NHLBI Program for Genomic Applications, Massachusetts General Hospital and Harvard Medical School, Boston, MA (http://pga.mgh.harvard.edu; accessed 10Nov2015) [45] made available by the *Pseudomonas Genome Database* (http://www.pseudomonas.com/; accessed 10Nov2015) [44].
(DOCX)

S2 Table Metal transport genes whose expression is differentially regulated. Expression of genes within PA14 that was grown in whole blood from the three severely burned patients was compared with the expression when PA14 was grown in whole blood from a healthy volunteer. Product names, functional classification(s), gene ontology terms, pathways, and functional predictions for PA14 genes were obtained from the MGH-ParaBioSys:NHLBI Program for Genomic Applications, Massachusetts General Hospital and Harvard Medical School, Boston, MA (http://pga.mgh.harvard.edu; accessed 10Nov2015) [45] made available by the *Pseudomonas Genome Database* (http://www.pseudomonas.com/; accessed 10Nov2015) [44].
(DOCX)

S3 Table ABC-transporter genes whose expression is enhanced. Gene expression within PA14 grown in whole blood from the three severely burned patients was compared with expression when PA14 was grown in whole blood from a healthy volunteer. Product names, functional classification(s), gene ontology terms, pathways, and functional predictions for PA14 genes were obtained from the MGH-ParaBioSys:NHLBI Program for Genomic Applications, Massachusetts General Hospital and Harvard Medical School, Boston, MA (http://pga.mgh.harvard.edu; accessed 10Nov2015) [45] made available by the *Pseudomonas Genome Database* (http://www.pseudomonas.com/; accessed 10Nov2015) [44].
(DOCX)

S4 Table ABC-transport genes whose expression is significantly reduced. Expression of genes within PA14 that was grown in whole blood from the three severely burned patients was compared with the expression when PA14 was grown in whole blood from a healthy volunteer. Product names, functional classification(s), gene ontology terms, pathways, and functional predictions for PA14 genes were obtained from the MGH-ParaBioSys:NHLBI Program for Genomic Applications, Massachusetts General Hospital and Harvard Medical School, Boston, MA (http://pga.mgh.harvard.edu; accessed 10Nov2015) [45] made available by the *Pseudomonas Genome Database* (http://www.pseudomonas.com/; accessed 10Nov2015) [44].
(DOCX)

S5 Table TonB-related genes that are differentially regulated. Expression of genes within PA14 that was grown in whole blood from the three severely burned patients was compared with the expression when PA14 was grown in whole blood from a healthy volunteer. Product names, functional classification(s), gene ontology terms, pathways, and functional predictions for PA14 genes were obtained from the MGH-ParaBioSys:NHLBI Program for Genomic Applications, Massachusetts General Hospital and Harvard Medical School, Boston, MA (http://pga.mgh.harvard.edu; accessed 10Nov2015) [45] made available by the *Pseudomonas Genome Database* (http://www.pseudomonas.com/; accessed 10Nov2015) [44].
(DOCX)

S6 Table Phosphonate transport genes whose expression is differentially regulated. Expression of genes within PA14 that was grown in whole blood from the three severely burned patients was compared with the expression when PA14 was grown in whole blood from a healthy volunteer. Product names, functional classification(s), gene ontology terms, pathways, and functional predictions for PA14 genes were obtained from the MGH-ParaBioSys:NHLBI Program for Genomic Applications, Massachusetts General Hospital and Harvard Medical School, Boston, MA (http://pga.mgh.harvard.edu; accessed 10Nov2015) [45] made available by the *Pseudomonas Genome Database* (http://www.pseudomonas.com/; accessed 10Nov2015) [44].
(DOCX)

S7 Table Motility related genes that are differentially regulated. Expression of genes within PA14 that was grown in whole blood from the three severely burned patients was compared with the expression when PA14 was grown in whole blood from a healthy volunteer. Product names, functional classification(s), gene ontology terms, pathways, and functional predictions for PA14 genes were obtained from the MGH-ParaBioSys:NHLBI Program for Genomic Applications, Massachusetts General Hospital and Harvard Medical School, Boston, MA (http://pga.mgh.harvard.edu; accessed 10Nov2015) [45] made available by the *Pseudomonas Genome Database* (http://www.pseudomonas.com/; accessed 10Nov2015) [44].
(DOCX)

## References

[1] Griswold JA (1993) White blood cell response to burn injury. Semin Nephrol 13: 409–415. 8351455

[2] Lederer JA, Rodrick ML, Mannick JA (1999) The effects of injury on the adaptive immune response. Shock 11: 153–159. 10188766 10.1097/00024382-199903000-00001

[3] Barret JP, Herndon DN (2003) Effects of burn wound excision on bacterial colonization and invasion. Plast Reconstr Surg 111: 744–750; discussion 751–742. 12560695 10.1097/01.PRS.0000041445.76730.23

[4] Church D, Elsayed S, Reid O, Winston B, Lindsay R (2006) Burn wound infections. Clin Microbiol Rev 19: 403–434. 16614255 10.1128/CMR.19.2.403-434.2006 PMC1471990

[5] Erol S, Altoparlak U, Akcay MN, Celebi F, Parlak M (2004) Changes of microbial flora and wound colonization in burned patients. Burns 30: 357–361. 15145194 10.1016/j.burns.2003.12.013

[6] Manson WL, Pernot PC, Fidler V, Sauer EW, Klasen HJ (1992) Colonization of burns and the duration of hospital stay of severely burned patients. J Hosp Infect 22: 55–63. 1358947 10.1016/0195-6701(92)90130-e

[7] Ramzy PI, Herndon DN, Wolf SE, Irtun O, Barret JP, Ramirez RJ, et al (1998) Comparison of wound culture and bronchial lavage in the severely burned child: implications for antimicrobial therapy. Arch Surg 133: 1275–1280. 9865643 10.1001/archsurg.133.12.1275

[8] Atiyeh BS, Gunn SW, Dibo SA (2008) Metabolic implications of severe burn injuries and their management: a systematic review of the literature. World J Surg 32: 1857–1869. 10.1007/s00268-008-9587-8 18454355

[9] Rodrick ML (1999) The effects of traumatic or burn injury on the humoral immune response. Sepsis 34: 235–238.

[10] Rafla K, Tredget EE (2011) Infection control in the burn unit. Burns 37: 5–15. 10.1016/j.burns.2009.06.198 20561750

[11] Fitzwater J, Purdue GF, Hunt JL, O'Keefe GE (2003) The risk factors and time course of sepsis and organ dysfunction after burn trauma. J Trauma 54: 959–966. 12777910 10.1097/01.TA.0000029382.26295.AB

[12] Mason AD Jr., McManus AT, Pruitt BA Jr. (1986) Association of burn mortality and bacteremia. A 25-year review. Arch Surg 121: 1027–1031. 3741098 10.1001/archsurg.1986.01400090057009

[13] Robson MC (1988) Burn sepsis. Crit Care Clin 4: 281–298. 3048588

[14] Branski LK, Al-Mousawi A, Rivero H, Jeschke MG, Sanford AP, Herndon DN. (2009) Emerging infections in burns. Surg Infect (Larchmt) 10: 389–397. 19810827 10.1089/sur.2009.024 PMC2956561

[15] Lyczak JB, Cannon CL, Pier GB (2000) Establishment of *Pseudomonas aeruginosa* infection: lessons from a versatile opportunist. Microbes Infect 2: 1051–1060. 10967285 10.1016/s1286-4579(00)01259-4

[16] Pier GB, Ramphal R (2010) *Pseudomonas aeruginosa* In: Mandell GL, Bennett JE, Dolin R, editors. Mandell, Douglas, and Bennett's Principles and Practice of Infectious Diseases. 7th ed. Philadelphia: Churchill Livingstone pp. 2835–2860.

[17] Sadikot RT, Blackwell TS, Christman JW, Prince AS (2005) Pathogen-host interactions in *Pseudomonas aeruginosa* pneumonia. Am J Respir Crit Care Med 171: 1209–1223. 15695491 10.1164/rccm.200408-1044SO PMC2718459

[18] van Delden C (2004) Virulence factors in Pseudomonas aeruginosa In: Ramos J-L, editor. *Pseudomonas*: Virulence and gene regulation. New York, NY: Kluwer Academic/Plenum Publishers pp. 3–45.

[19] Mahenthiralingam E, Campbell ME, Speert DP (1994) Nonmotility and phagocytic resistance of *Pseudomonas aeruginosa* isolates from chronically colonized patients with cystic fibrosis. Infect Immun 62: 596–605. 8300217 10.1128/iai.62.2.596-605.1994 PMC186146

[20] Romling U, Fiedler B, Bosshammer J, Grothues D, Greipel J, vonder Hardt H, et al (1994) Epidemiology of chronic *Pseudomonas aeruginosa* infections in cystic fibrosis. J Infect Dis 170: 1616–1621. 7996008 10.1093/infdis/170.6.1616

[21] Govan JR, Deretic V (1996) Microbial pathogenesis in cystic fibrosis: mucoid *Pseudomonas aeruginosa* and *Burkholderia cepacia*. Microbiol Rev 60: 539–574. 8840786 10.1128/mr.60.3.539-574.1996 PMC239456

[22] Spencer DH, Kas A, Smith EE, Raymond CK, Sims EH, Hastings M, et al (2003) Whole-genome sequence variation among multiple isolates of *Pseudomonas aeruginosa*. J Bacteriol 185: 1316–1325. 12562802 10.1128/JB.185.4.1316-1325.2003 PMC142842

[23] De Vos D, De Chial M, Cochez C, Jansen S, Tummler B, Meyer JM, et al (2001) Study of pyoverdine type and production by *Pseudomonas aeruginosa* isolated from cystic fibrosis patients: prevalence of type II pyoverdine isolates and accumulation of pyoverdine-negative mutations. Arch Microbiol 175: 384–388. 11409549 10.1007/s002030100278

[24] Jain M, Ramirez D, Seshadri R, Cullina JF, Powers CA, Schulert GS, et al (2004) Type III secretion phenotypes of *Pseudomonas aeruginosa* strains change during infection of individuals with cystic fibrosis. J Clin Microbiol 42: 5229–5237. 15528719 10.1128/JCM.42.11.5229-5237.2004 PMC525189

[25] Smith EE, Buckley DG, Wu Z, Saenphimmachak C, Hoffman LR, D’Argenio DA, et al (2006) Genetic adaptation by *Pseudomonas aeruginosa* to the airways of cystic fibrosis patients. Proc Natl Acad Sci U S A 103: 8487–8492. 16687478 10.1073/pnas.0602138103 PMC1482519

[26] D'Argenio DA, Wu M, Hoffman LR, Kulasekara HD, Deziel E, Smith EE, et al (2007) Growth phenotypes of *Pseudomonas aeruginosa lasR* mutants adapted to the airways of cystic fibrosis patients. Mol Microbiol 64: 512–533. 17493132 10.1111/j.1365-2958.2007.05678.x PMC2742308

[27] Dingemans J, Ye L, Hildebrand F, Tontodonati F, Craggs M, Bilocq F, et al (2014) The deletion of TonB-dependent receptor genes is part of the genome reduction process that occurs during adaptation of *Pseudomonas aeruginosa* to the cystic fibrosis lung. Pathog Dis 71: 26–38. 10.1111/2049-632X.12170 24659602

[28] Rumbaugh KP, Griswold JA, Iglewski BH, Hamood AN (1999) Contribution of quorum sensing to the virulence of *Pseudomonas aeruginosa* in burn wound infections. Infect Immun 67: 5854–5862. 10531240 10.1128/iai.67.11.5854-5862.1999 PMC96966

[29] Turner KH, Everett J, Trivedi U, Rumbaugh KP, Whiteley M (2014) Requirements for *Pseudomonas aeruginosa* acute burn and chronic surgical wound infection. PLoS Genet 10: e1004518 10.1371/journal.pgen.1004518 25057820 PMC4109851

[30] Graham MR, Virtaneva K, Porcella SF, Barry WT, Gowen BB, Johnson CR, et al (2005) Group A *Streptococcus* transcriptome dynamics during growth in human blood reveals bacterial adaptive and survival strategies. Am J Pathol 166: 455–465. 15681829 10.1016/S0002-9440(10)62268-7 PMC1602339

[31] Berger MM, Rothen C, Cavadini C, Chiolero RL (1997) Exudative mineral losses after serious burns: a clue to the alterations of magnesium and phosphate metabolism. Am J Clin Nutr 65: 1473–1481. 9129479 10.1093/ajcn/65.5.1473

[32] Loven L, Larsson L, Nordstrom H, Lennquist S (1986) Serum phosphate and 2,3-diphosphoglycerate in severely burned patients after phosphate supplementation. J Trauma 26: 348–352. 3083112 10.1097/00005373-198604000-00008

[33] Kealey CP, Whelan SA, Chun YJ, Soojung CH, Tulloch AW, Mohanchandra KP, et al (2010) *In vitro* hemocompatibility of thin film nitinol in stenotic flow conditions. Biomaterials 31: 8864–8871. 10.1016/j.biomaterials.2010.08.014 20810163 PMC2949484

[34] Auten-Ochs J-C (2014) Blood ions and proteins influence *Pseudomonas aeruginosa* virulence by significantly altering the expression of virulence and virulence-related genes. Lubbock, Texas: Texas University Health Sciences Center 224 p.

[35] Rahme LG, Stevens EJ, Wolfort SF, Shao J, Tompkins RG, Ausubel FM. (1995) Common virulence factors for bacterial pathogenicity in plants and animals. Science 268: 1899–1902. 7604262 10.1126/science.7604262

[36] Cole SJ, Records AR, Orr MW, Linden SB, Lee VT (2014) Catheter-associated urinary tract infection by *Pseudomonas aeruginosa* is mediated by exopolysaccharide-independent biofilms. Infect Immun 82: 2048–2058. 10.1128/IAI.01652-14 24595142 PMC3993445

[37] Garcia-Contreras R, Perez-Eretza B, Lira-Silva E, Jasso-Chavez R, Coria-Jimenez R, Rangel-Vega A, et al (2014) Gallium induces the production of virulence factors in *Pseudomonas aeruginosa*. Pathog Dis 70: 95–98. 10.1111/2049-632X.12105 24151196

[38] Lau GW, Goumnerov BC, Walendziewicz CL, Hewitson J, Xiao W, Mahajan-Miklos S, et al (2003) The *Drosophila melanogaster* Toll pathway participates in resistance to infection by the Gram-negative human pathogen *Pseudomonas aeruginosa*. Infect Immun 71: 4059–4066. 12819096 10.1128/IAI.71.7.4059-4066.2003 PMC162001

[39] Zhang L, Fritsch M, Hammond L, Landreville R, Slatculescu C, Colavita A, et al (2013) Identification of genes involved in *Pseudomonas aeruginosa* biofilm-specific resistance to antibiotics. PLoS One 8: e61625 10.1371/journal.pone.0061625 23637868 PMC3634840

[40] Rosner R (1974) Evaluation of four blood culture systems using parallel culture methods. Appl Microbiol 28: 245–247. 4851923 10.1128/am.28.2.245-247.1974 PMC186694

[41] Tenney JH, Reller LB, Wang WL, Cox RL, Mirrett S (1982) Comparative evaluation of supplemented peptone broth with sodium polyanetholesulfonate and trypticase soy broth with sodium amylosulfate for detection of septicemia. J Clin Microbiol 16: 107–110. 6286716 10.1128/jcm.16.1.107-110.1982 PMC272304

[42] Mi H, Muruganujan A, Thomas PD (2013) PANTHER in 2013: modeling the evolution of gene function, and other gene attributes, in the context of phylogenetic trees. Nucleic Acids Res 41: D377–386. 10.1093/nar/gks1118 23193289 PMC3531194

[43] Mi H, Muruganujan A, Casagrande JT, Thomas PD (2013) Large-scale gene function analysis with the PANTHER classification system. Nat Protoc 8: 1551–1566. 10.1038/nprot.2013.092 23868073 PMC6519453

[44] Winsor GL, Lam DK, Fleming L, Lo R, Whiteside MD, Yu NY, et al (2011) *Pseudomonas* Genome Database: improved comparative analysis and population genomics capability for *Pseudomonas* genomes. Nucleic Acids Res 39: D596–600. 10.1093/nar/gkq869 20929876 PMC3013766

[45] Lee DG, Urbach JM, Liberati NT, Feinbaum RL, Miyata S, Diggins LT, et al (2006) Genomic analysis reveals that *Pseudomonas aeruginosa* virulence is combinatorial. Genome Biol 7: R90 17038190 10.1186/gb-2006-7-10-r90 PMC1794565

[46] Gallagher LA, McKnight SL, Kuznetsova MS, Pesci EC, Manoil C (2002) Functions required for extracellular quinolone signaling by *Pseudomonas aeruginosa*. J Bacteriol 184: 6472–6480. 12426334 10.1128/JB.184.23.6472-6480.2002 PMC135424

[47] Albus AM, Pesci EC, Runyen-Janecky LJ, West SE, Iglewski BH (1997) Vfr controls quorum sensing in *Pseudomonas aeruginosa*. J Bacteriol 179: 3928–3935. 9190808 10.1128/jb.179.12.3928-3935.1997 PMC179201

[48] Diggle SP, Winzer K, Lazdunski A, Williams P, Camara M (2002) Advancing the quorum in *Pseudomonas aeruginosa*: MvaT and the regulation of N-acylhomoserine lactone production and virulence gene expression. J Bacteriol 184: 2576–2586. 11976285 10.1128/JB.184.10.2576-2586.2002 PMC135012

[49] Heurlier K, Denervaud V, Pessi G, Reimmann C, Haas D (2003) Negative control of quorum sensing by RpoN (sigma 54) in *Pseudomonas aeruginosa* PAO1. J Bacteriol 185: 2227–2235. 12644493 10.1128/JB.185.7.2227-2235.2003 PMC151487

[50] Juhas M, Wiehlmann L, Huber B, Jordan D, Lauber J, Salunkhe P, et al (2004) Global regulation of quorum sensing and virulence by VqsR in *Pseudomonas aeruginosa*. Microbiology 150: 831–841. 15073293 10.1099/mic.0.26906-0

[51] Pessi G, Williams F, Hindle Z, Heurlier K, Holden MT, Camara M, et al (2001) The global posttranscriptional regulator RsmA modulates production of virulence determinants and N-acylhomoserine lactones in *Pseudomonas aeruginosa*. J Bacteriol 183: 6676–6683. 11673439 10.1128/JB.183.22.6676-6683.2001 PMC95500

[52] Reimmann C, Beyeler M, Latifi A, Winteler H, Foglino M, Lazdunski A, et al (1997) The global activator GacA of *Pseudomonas aeruginosa* PAO positively controls the production of the autoinducer N-butyryl-homoserine lactone and the formation of the virulence factors pyocyanin, cyanide, and lipase. Mol Microbiol 24: 309–319. 9159518 10.1046/j.1365-2958.1997.3291701.x

[53] Nakagami G, Sanada H, Sugama J, Morohoshi T, Ikeda T, Ohta Y. (2008) Detection of *Pseudomonas aeruginosa* quorum sensing signals in an infected ischemic wound: an experimental study in rats. Wound Repair Regen 16: 30–36. 10.1111/j.1524-475X.2007.00329.x 18211577

[54] Kruczek C, Qaisar U, Colmer-Hamood JA, Hamood AN (2014) Serum influences the expression of *Pseudomonas aeruginosa* quorum-sensing genes and QS-controlled virulence genes during early and late stages of growth. Microbiologyopen 3: 64–79. 24436158 10.1002/mbo3.147 PMC3937730

[55] Cornelis P (2010) Iron uptake and metabolism in pseudomonads. Appl Microbiol Biotechnol 86: 1637–1645. 10.1007/s00253-010-2550-2 20352420

[56] Wilderman PJ, Vasil AI, Johnson Z, Wilson MJ, Cunliffe HE, Lamont IL, et al (2001) Characterization of an endoprotease (PrpL) encoded by a PvdS-regulated gene in *Pseudomonas aeruginosa*. Infect Immun 69: 5385–5394. 11500408 10.1128/IAI.69.9.5385-5394.2001 PMC98648

[57] Kim SJ, Park RY, Kang SM, Choi MH, Kim CM, Shin SH. (2006) *Pseudomonas aeruginosa* alkaline protease can facilitate siderophore-mediated iron-uptake via the proteolytic cleavage of transferrins. Biol Pharm Bull 29: 2295–2300. 17077532 10.1248/bpb.29.2295

[58] Kruczek C, Wachtel M, Alabady MS, Payton PR, Colmer-Hamood JA, Hamood AN. (2012) Serum albumin alters the expression of iron-controlled genes in *Pseudomonas aeruginosa*. Microbiology 158: 353–367. 10.1099/mic.0.053371-0 22053004 PMC3352286

[59] Colmer JA, Hamood AN (1998) Characterization of *ptxS*, a *Pseudomonas aeruginosa* gene which interferes with the effect of the exotoxin A positive regulatory gene, *ptxR*. Mol Gen Genet 258: 250–259. 9645431 10.1007/s004380050729

[60] Youard ZA, Wenner N, Reimmann C (2011) Iron acquisition with the natural siderophore enantiomers pyochelin and enantio-pyochelin in *Pseudomonas* species. Biometals 24: 513–522. 10.1007/s10534-010-9399-9 21188474

[61] Dumas Z, Ross-Gillespie A, Kummerli R (2013) Switching between apparently redundant iron-uptake mechanisms benefits bacteria in changeable environments. Proc Biol Sci 280: 20131055 10.1098/rspb.2013.1055 23760867 PMC3712426

[62] Hauser AR (2009) The type III secretion system of *Pseudomonas aeruginosa*: infection by injection. Nat Rev Microbiol 7: 654–665. 10.1038/nrmicro2199 19680249 PMC2766515

[63] Frank DW (1997) The exoenzyme S regulon of *Pseudomonas aeruginosa*. Mol Microbiol 26: 621–629. 9427393 10.1046/j.1365-2958.1997.6251991.x

[64] Horsman SR, Moore RA, Lewenza S (2012) Calcium chelation by alginate activates the type III secretion system in mucoid *Pseudomonas aeruginosa* biofilms. PLoS One 7: e46826 10.1371/journal.pone.0046826 23056471 PMC3466208

[65] Kim J, Ahn K, Min S, Jia J, Ha U, Wu D, et al (2005) Factors triggering type III secretion in *Pseudomonas aeruginosa*. Microbiology 151: 3575–3587. 16272380 10.1099/mic.0.28277-0

[66] Ochsner UA, Johnson Z, Vasil ML (2000) Genetics and regulation of two distinct haem-uptake systems, *phu* and *has*, in *Pseudomonas aeruginosa*. Microbiology 146 (Pt 1): 185–198. 10658665 10.1099/00221287-146-1-185

[67] Wandersman C, Delepelaire P (2004) Bacterial iron sources: from siderophores to hemophores. Annu Rev Microbiol 58: 611–647. 15487950 10.1146/annurev.micro.58.030603.123811

[68] Gibson RL, Burns JL, Ramsey BW (2003) Pathophysiology and management of pulmonary infections in cystic fibrosis. Am J Respir Crit Care Med 168: 918–951. 14555458 10.1164/rccm.200304-505SO

[69] Lamarche MG, Wanner BL, Crepin S, Harel J (2008) The phosphate regulon and bacterial virulence: a regulatory network connecting phosphate homeostasis and pathogenesis. FEMS Microbiol Rev 32: 461–473. 10.1111/j.1574-6976.2008.00101.x 18248418

[70] Loghmani S, Maracy MR, Kheirmand R (2010) Serum phosphate level in burn patients. Burns 36: 1112–1115. 10.1016/j.burns.2009.12.009 20409642

[71] Ghorbel S, Kormanec J, Artus A, Virolle MJ (2006) Transcriptional studies and regulatory interactions between the *phoR-phoP* operon and the *phoU*, *mtpA*, and *ppk* genes of *Streptomyces lividans* TK24. J Bacteriol 188: 677–686. 16385057 10.1128/JB.188.2.677-686.2006 PMC1347273

[72] Kornberg A, Rao NN, Ault-Riche D (1999) Inorganic polyphosphate: a molecule of many functions. Annu Rev Biochem 68: 89–125. 10872445 10.1146/annurev.biochem.68.1.89

[73] Rashid MH, Kornberg A (2000) Inorganic polyphosphate is needed for swimming, swarming, and twitching motilities of *Pseudomonas aeruginosa*. Proc Natl Acad Sci U S A 97: 4885–4890. 10758151 10.1073/pnas.060030097 PMC18327

[74] Rashid MH, Rumbaugh K, Passador L, Davies DG, Hamood AN, Iglewski BH, et al (2000) Polyphosphate kinase is essential for biofilm development, quorum sensing, and virulence of *Pseudomonas aeruginosa*. Proc Natl Acad Sci U S A 97: 9636–9641. 10931957 10.1073/pnas.170283397 PMC16917

[75] Benjamini Y, Hochberg Y (1995) Controlling the false discovery rate: a practical and powerful approach to multiple testing. J Roy Stat Soc B 57: 289–300.

