# Supplementary figures and images for: Major Transcriptome Changes Accompany the Growth of Pseudomonas aeruginosa in Blood from Patients with Severe Thermal Injuries
Source: PLoS One. 2016 Mar 2;11(3):e0149229. doi: 10.1371/journal.pone.0149229 (PMC4774932; doi:10.1371/journal.pone.0149229)

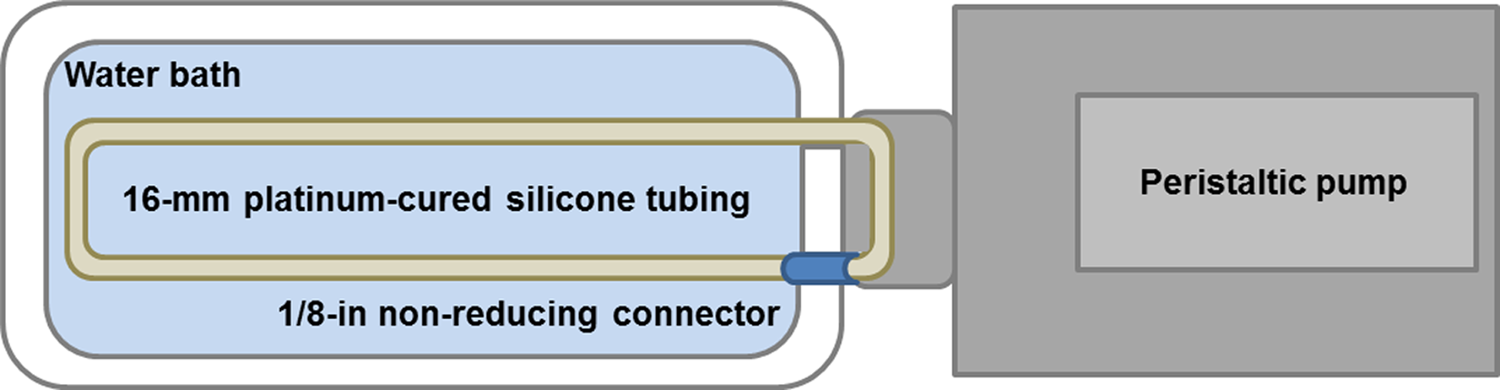

Supplement: S1 Fig — The peristaltic pump was set at a flow rate of 176 mL/min, the water bath maintained at 37°C, and 2-ft sections of 16-mm platinum-cured silicone tubing (one loop is shown, but up to 4 loops can be used at one time) were used to hold the blood. The non-reducing connecter minimizes trauma to the red blood cells. (TIF) [file pone.0149229.s001.tif]

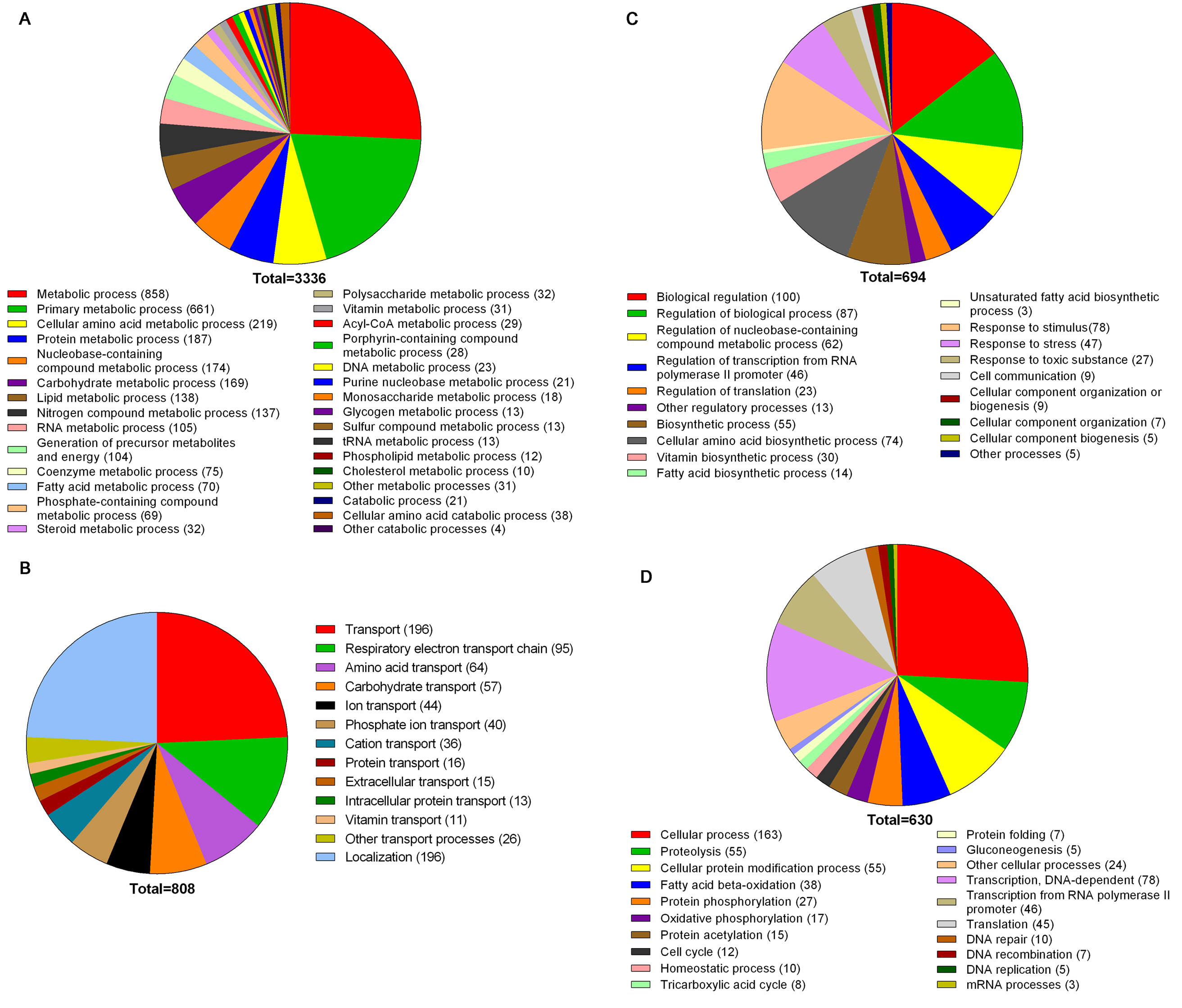

Supplement: S2 Fig — Genes whose expression was differentially regulated when the bacteria were grown in whole blood from the patients compared to the healthy volunteer were analyzed using PANTHER GO-Slim Biological Proccess analysis [42,43]. A total of 6875 GO terms were assigned to the 2348 mapped genes, with 1407 of these being unclassified. The remaining 5468 terms represented metabolic and catabolic processes– 3336 (A), transport and localization– 808 (B), biological regulation, biosynthetic processes, responses to stimuli, and cellular component organization or biogenesis– 694 (C), and cellular processes– 630 (D). (TIF) [file pone.0149229.s002.tif]
