# Supplementary material for: Major Transcriptome Changes Accompany the Growth of Pseudomonas aeruginosa in Blood from Patients with Severe Thermal Injuries
Source: PLoS One. 2016 Mar 2;11(3):e0149229. doi: 10.1371/journal.pone.0149229 (PMC4774932; doi:10.1371/journal.pone.0149229)
Supplement: S1 Table — Gene expression within PA14 grown in whole blood from the three severely burned patients was compared with expression when PA14 was grown in whole blood from a healthy volunteer. Product names, functional classification(s), gene ontology terms, pathways, and functional predictions for PA14 genes were obtained from the MGH-ParaBioSys:NHLBI Program for Genomic Applications, Massachusetts General Hospital and Harvard Medical School, Boston, MA (http://pga.mgh.harvard.edu; accessed 10Nov2015) [45] made available by the Pseudomonas Genome Database (http://www.pseudomonas.com/; accessed 10Nov2015) [44]. (DOCX) [file pone.0149229.s005.docx]

**S1 Table. Differentially expressed heme transport and utilization genes.**

| **Gene/ORF** | **Product^a^** | **Functional classification(s) // Gene ontology terms^a^** | **Pathways // Functional predictions^a^** | **Pt 1** | **Pt 2** | **Pt 3** |
| --- | --- | --- | --- | --- | --- | --- |
| *hasR* | Heme uptake outer membrane receptor HasR | Transport of small molecules // Transport; membrane; outer membrane; transporter activity; receptor activity; heme binding | // TonB-dependent hemoglobin/transferrin/lactoferrin receptor family protein | -5^b^ | -5 | -6 |
| *hasAP* | Heme acquisition protein HasAP | Transport of small molecules // No GO terms listed | // Heme-binding protein A (HasA) | -26 | -17 | -16 |
| *hasD^c^* | Transport protein HasD | Protein secretion/export apparatus // Transport; transmembrane transport; protein secretion by T1SS; integral comp of membrane; type I protein secretion system complex; protein transporter activity; ATPase activity, coupled to transmembrane movement of substances; ATP binding | ABC transporters // Type I secretion system ATPase; ABC transporter integral membrane type-1 fused domain profile | -3 | -3 | -2 |
| *PA14_20050* | Outer membrane protein | Protein secretion/export apparatus // Transport; protein transport; outer membrane; transporter activity | // Type I secretion outer membrane protein, TolC family; outer membrane efflux protein | -3 | -3 | -3 |
| *PA14_45340^c^* | Heme exporter protein | Transport of small molecules // Heme transport; integral component of membrane | ABC transporters // Heme exporter protein D (CcmD) | 4 | 4 | 5 |
| *ccmC* | Heme exporter protein CcmC | Transport of small molecules // Heme transport; cytochrome complex assembly; membrane; heme binding; heme transporter activity | ABC transporters // Cytochrome c-type biogenesis protein CcmC | 6 | 5 | 5 |
| *ccmE* | Cytochrome c-type biogenesis protein CcmE | Energy metabolism // Cytochrome complex assembly; protein-heme linkage; plasma membrane | // Cytochrome c-type biogenesis protein CcmE; nucleic acid-binding, OB fold |  |  |  |
| *ccmF* | Cytochrome c-type biogenesis protein CcmF | Energy metabolism // Type IV pilus-dependent motility; heme transport; bacterial-type flagellum-dependent swarming motility; bacterial-type flagellum-dependent swimming motility; cytochrome complex assembly; pyoverdine biosynthetic process; membrane; heme transporter activity; catalase activity; heme binding | // Cytochrome c-type biogenesis protein signature; cytochrome C assembly protein |  |  |  |
| *ccmG* | Cytochrome c-type biogenesis protein CcmG | Translation, post-translational modification, degradation; chaperones and heat shock proteins; energy metabolism // Cytochrome complex assembly; cell redox homeostasis; outer membrane-bounded periplasmic space; disulfide oxidoreductase activity | // Periplasmic protein thiol:disulfide oxidoreductases, DsbE family; thioredoxin domain profile |  |  |  |
| *ccmH* | Cytochrome c-type biogenesis protein CcmH | Energy metabolism // No GO terms listed | // Cytochrome C biogenesis protein |  |  |  |
| *cycH* | Cytochrome c-type biogenesis protein | Energy metabolism // Protein binding | // Cytochrome C biogenesis protein; tetratricopeptide repeat-containing domain; tetratricopep-tide-like helical domain |  |  |  |
| *PA14_45260* | Hypothetical protein | Hypothetical, unclassified, unknown // No GO terms listed | // |  |  |  |
| *ccmB* | Heme exporter protein CcmB | Transport of small molecules; membrane proteins // Heme transport; cytochrome complex assembly; membrane; heme transporter activity | ABC transporters // Cytochrome c-type biogenesis protein CcmC | 3 | 3 | 3 |
| *PA14_47380* | Heme utilization protein | Transport of small molecules // Transport; membrane; outer membrane transporter activity; receptor activity | // TonB-dependent hemoglobin/transferrin/lactoferrin receptor | 2 | 2 | 2 |
| *PA14_62350* | Heme/hemoglobin uptake outer membrane receptor PhuR | Transport of small molecules // Transport; membrane; outer membrane; transporter activity; receptor activity | // TonB-dependent haemoglobin/transferrin/lactoferrin receptor | *2* | *3* | *2* |
| *PA14_15930^c^* | Hemolysin | Transport of small molecules; membrane proteins // Oxidation-reduction process; catalytic activity; oxidation-reduction activity, acting on CH-OH group; flavin adenine dinucleotide binding; adenyl nucleotide binding | // Domain in cystathionine beta-synthase and other proteins; CO dehydrogenase flavoprotein-like, FAD-binding subdomain 2; transporter-associated domain | 3 | 3 | 3 |
| *PA14_15940* | Hypothetical protein | Membrane proteins // Cytochrome complex assembly; heme binding | // Cytochrome c assembly protein | 2 | 2 | 2 |
| *hmuV* | Hemin importer ATP-binding subunit | Transport of small molecules // Heme transport; membrane; heme-transporting ATPase activity; ATP binding | Sulfur metabolism; ABC transporters // Hemin import ATP-binding protein HmuV family profile; ATP-binding cassette, ABC transporter-type domain profile | 2 | 2 | 2 |
| *fpr* | Ferredoxin-NADP+ reductase | Energy metabolism; biosynthesis of cofactors, prosthetic groups, and carriers // Oxidation-reduction process; oxidoreductase activity | // Ferredoxin reductase-type FAD-ginding domain; flavoprotein pyridine nucleotide cytochrome reductase | 3 | 3 | 3 |
| *PA14_00490* | Hemolysin activation/secretion protein | Protein secretion/export apparatus // Protein transport | // Haemolysin secretion/activation protein ShlB/FhaC/HecB; haemolysin activator HlyB | 2 | 2 | 2 |
| *PA14_58890* | Hemolysin activation/secretion protein | Hypotethetical, unclassified, unknown; protein secretion/export apparatus // No GO terms listed | // Polypeptide-transport-associated, ShlB-type | -3 | -2 | -1 |
| *PA14_63900* | Hemolysin III | Membrane proteins // Cytolysis; Integral component of membrane | // Channel protein hemolysin III family | -5 | -10 | -7 |
| *plcH^c^* | Hemolytic phospholipase C | Secreted factors // Lipid catabolic process; hydrolase activity (ester bonds); phospholipase C activity; phosphatidlycholine phospholipase C activity | Metabolic pathways; inositol phosphate metabolism; glcerophospholipid metabolism // Twin-arginine translocation pathway, signal sequence; bacterial phospholipase C, phosphocholine-specific; phosphoesterase | -4 | -5 | -7 |
| *plcR* | Phospholipase accessory protein PlcR | Secreted factors // No GO terms listed | // Phospholipase C accessory prpotein PlcR | -4 | -9 | -15 |
| *PA14_53380* | Glycosyl transferase family protein | Putative enzymes // Biosynthetic process | // Glycosyl transferase, family 1; glycosyltransferase subfamily 4 | -48 | -30 | -26 |
| *nemO* | Heme oxygenase | Biosynthesis of cofactors, prosthetic groups, and carriers // Oxidation-reduction process; heme oxidation; heme oxygenase (decyclizing) activity | Porphyrin and chlorophyll metabolism // Haem oxygenase-like, multi-helical | -3 | -5 | -4 |
| *nirH^c^* | Hypothetical protein | Hypothetical, unclassified, unknown; energy metabolism, biosynthesis of cofactors, prosthetic groups, and carriers // No GO terms listed | // Winged helix-turn-helix DNA-binding domain | -3 | 1 | -4 |
| *nirL* | Heme d1 biosythesis protein NirL | Hypothetical, unclassified, unknown; energy metabolism, biosynthesis of cofactors, prosthetic groups, and carriers // No GO terms listed | // | -3 | 1 | -3 |
| *PA14_06170* | Transcriptional regulator | Transcriptional regulator, energy metabolism, biosynthesis of cofactors, prosthetic groups, and carriers // Regulation of transcription DNA-templated; sequence-specific DNA binding | // Winged helix-turn-helix DNA-binding domain; AsnC-type HTH domain | -3 | 1 | -4 |
| *nirF* | Heme d1 biosythesis protein NirF | Energy metabolism // No GO terms listed | // Cytochrome cd1-nitrite reductase-like, haem d1 domain; prokaryotic membrane lipoprotein lipid attachment site profile | -4 | 2 | -6 |
| *nirC* | c-type cytochrome | Energy metabolism; biosynthesis of cofactors, prosthetic groups, and carriers // Heme binding; electron carrier activity | // Cytochrome c-like domain | -19 | 1 | -23 |
| *nirS* | Nitrate reductase | Energy metabolism // Heme binding; electron carrier activity; protein binding | Nitrogen metabolism; microbial metabolism in diverse environments // Cytochrome cdl-nitrite reductase-like, haem d1 domain; cytochrome c-like domain | -3 | -1 | -3 |
| *nirQ* | Regulatory protein NirQ | Energy metabolism, central intermediary metabolism // ATPase activity, ATP binding | // CbbQ/NirQ/NorQ, C-terminal; ATPase, dynein-related, AAA domain | -3 | -1 | -3 |

^a^Product names, functional classification(s), gene ontology terms, pathways, and functional predictions for PA14 genes were obtained from the MGH-ParaBioSys:NHLBI Program for Genomic Applications, Massachusetts General Hospital and Harvard Medical School, Boston, MA (<http://pga.mgh.harvard.edu>; accessed 10Nov2015) [1] made available by the *Pseudomonas Genome Database* (<http://www.pseudomonas.com/>; accessed 10Nov2015) [2].

^b^Gene expression within PA14 grown in whole blood from the three severely burned patients (Pt) was compared with expression when PA14 was grown in whole blood from a healthy volunteer.

^c^Genes found in operons are color-coded, with related genes in close proximity highlighted a lighter color.

**References**

1. Lee DG, Urbach JM, Liberati NT, Feinbaum RL, Miyata S, Diggins LT, et al. (2006) Genomic analysis reveals that *Pseudomonas aeruginosa* virulence is combinatorial. Genome Biol 7: R90.

2. Winsor GL, Lam DK, Fleming L, Lo R, Whiteside MD, Yu NY, et al. (2011) *Pseudomonas* Genome Database: improved comparative analysis and population genomics capability for *Pseudomonas* genomes. Nucleic Acids Res 39: D596-600.
