# Supplementary material for: Major Transcriptome Changes Accompany the Growth of Pseudomonas aeruginosa in Blood from Patients with Severe Thermal Injuries
Source: PLoS One. 2016 Mar 2;11(3):e0149229. doi: 10.1371/journal.pone.0149229 (PMC4774932; doi:10.1371/journal.pone.0149229)
Supplement: S2 Table — Expression of genes within PA14 that was grown in whole blood from the three severely burned patients was compared with the expression when PA14 was grown in whole blood from a healthy volunteer. Product names, functional classification(s), gene ontology terms, pathways, and functional predictions for PA14 genes were obtained from the MGH-ParaBioSys:NHLBI Program for Genomic Applications, Massachusetts General Hospital and Harvard Medical School, Boston, MA (http://pga.mgh.harvard.edu; accessed 10Nov2015) [45] made available by the Pseudomonas Genome Database (http://www.pseudomonas.com/; accessed 10Nov2015) [44]. (DOCX) [file pone.0149229.s006.docx]

**S2 Table. Metal transport genes whose expression is differentially regulated.**

| **Gene/ORF** | **Product^a^** | **Functional classification(s) // Gene ontology terms^a^** | **Pathways // Functional predictions^a^** | **Ion** | **Pt 1^b^** | **Pt 2** | **Pt 3** |
| --- | --- | --- | --- | --- | --- | --- | --- |
| *PA14_56670^c^* | Hypothetical protein | Hypothetical, unclassified, unknown // No GO terms listed | // Transcriptional regulator HTH-type, FeoC | Fe | 14 | 9 | 13 |
| *feoB* | Ferrous iron transport protein B | Transport of small molecules; membrane proteins // Ferrous iron transport; integral component of membrane; ferrous iron transmembrane transporter activity; nucleoside binding; GTP binding | // Ferrous iron transport protein B | Fe | 13 | 12 | 11 |
| *feoA* | Ferrous iron transport protein A | Hypothetical, unclassified, unknown; transport of small molecules // Transition metal ion binding | // Ferrous iron transporter FeoA domain | Fe | 7 | 8 | 5 |
| *PA14_38220* | Hypothetical protein | Transport of small molecules // Oxidation-reduction process; oxidoreductase activity | // Ferredoxin reductase-type FAD binding domain profile; siderophore-interacting FAD-binding domain | Fe | -4 | -5 | -5 |
| *bfrB* | Bacterioferritin | Transport of small molecules; adaptation, protection // Cellular iron ion homeostasis; intracellular sequestering of iron ion; iron ion transport; ferric iron binding | Porphyrin and chlorophyll metabolism // Bacterioferritin signature; Ferritin-like domain | Fe | -4 | -3 | -4 |
| *fepD^c^* | Ferric enterobactin transport protein FepD | Transport of small molecules // Membrane; transporter activity | ABC transporters // FecCD transport family; ABC transporter, permease protein | Fe | 3 | 1 | 2 |
| *fepB* | Iron-enterobactin transporter periplasmic binding protein | Transport of small molecules // Binding | ABC transporters // Iron siderophore/ cobalamin periplasmic-binding domain profile; ABC transporter periplasmic binding domain | Fe | -3 | -6 | -5 |
| *hitA^c^* | Ferric iron-binding periplasmic protein HitA | Transport of small molecules // No GO terms listed | ABC transporters // Ferric binding protein | Fe | -2 | -2 | -2 |
| *hitB* | Iron ABC transporter, permease | Transport of small molecule; membrane proteins // Transport | ABC transporters // ABC transporter integral membrane type-1 domain profile | Fe | 2 | 2 | 2 |
| *PA14_13170* | Metal transporting P-type ATPase | Transport of small molecules // metal ion transport; cation transport; integral component of membrane; copper ion binding; metal ion binding; cation-transporting ATPase activity; nucleotide binding | // Heavy-metal-associated domain profile.; copper ion binding protein; copper-transporting ATPase signature | Cu | 3 | 2 | 2 |
| *PA14_16660* | Metal-transporting P-type ATPase | Transport of small molecules // Metal ion transport; cation transport; integral component of membrane; metal ion binding; cation-transporting ATPase activity; nucleotide binding | // Heavy-metal-associated domain profile; cadmium-transporting ATPase signature | Cd | -3 | -3 | -2 |
| *PA14_18070* | Periplasmic metal-binding protein | Transport of small molecules // Metal ion transport; metal ion binding | // Heavy-metal-associated domain (copper ion-binding) | Cu | -3 | -3 | -5 |
| *nosL^c^* | NosL | Energy metabolism // No GO terms listed | // Nitrous oxide reductase accessory protein | Cu | 5 | 6 | 7 |
| *nosY* | NosY | Energy metabolism // No GO terms listed | // ABC-2 family transporter protein involved in multi-copper enzyme maturation, permease component | Cu | 6 | 5 | 5 |
| *nosD* | Copper ABC transporter periplasmic substrate-binding protein | Transport of small molecules // No GO terms listed | // Nitrous oxide reductase family maturation protein NosD | Cu | -2 | -2 | -2 |
| *nosR* | Regulatory protein NosR | Transcriptional regulators; energy metabolism; membrane proteins // Positive regulation of transcription, DNA-templated; membrane; iron-sulfur cluster binding; FMN binding; DNA binding | // 4Fe-4S binding domain; FMN-binding; nitrous oxide reductase expression regulator | Fe-S | -2 | -2 | -3 |
| *oprC^c^* | Outer membrane copper receptor OprC | Transport of small molecules // Transport; membrane; transporter activity; receptor activity | // TonB-dependent copper receptor | Cu | 4 | 5 | 5 |
| *PA14_15080* | Hypothetical protein | Membrane proteins // no GO terms listed | // PepSY-associated TM helix | Cu | 4 | 4 | 4 |
| *znuB* | ABC zinc transporter permease ZnuB | Transport of small; membrane proteins // Response to zinc ion; transport; membrane; ATPase activity, coupled to transmembrane movement of substances; ATP binding | ABC transporters // ABC 3 transport family | Zn | 2 | 2 | 2 |
| *czcC* | CzcC family cobalt/zinc/cadmium efflux transporter outer membrane protein | Transport of small molecules // Transport; transporter activity | // Outer membrane efflux protein | Co/Zn/Cd | -7 | -7 | -9 |
| *czcB* | Cobalt/zinc/cadmium efflux RND transporter, membrane fusion protein, CzcB family | Transport of small molecules // Transmembrane transport; membrane | // Efflux transporter, RND family, MFP subunit | Co/Zn/Cd | -3 | -2 | -2 |
| *mntH2* | Manganese transport protein MntH | Transport of small molecules; membrane proteins // Transport; membrane; transporter activity | // Metal ion transporter, metal ion (Mn2+/Fe2+) transporter (Nramp) family | Mn | -2 | -2 | -2 |
| *PA14_61330* | Magnesium transporter, MgtC family | Transport of small molecules // Membrane | // MgtC family | Mg | -5 | -6 | -5 |
| *PA14_33530^c^* | Hypothetical protein | Hypothetical, unclassified, unknown; motility and attachment // Metal ion transport; metal ion binding | // Periplasmic solute binding protein family | Mn/Zn | 4 | 5 | 5 |
| *PA14_33540* | ABC transporter permease | Transport of small molecules; Membrane proteins // Transport; membrane; ATP binding; ATPase activity, coupled to transmembrane movement of substances | ABC transporters // ABC-type Mn2+/Zn2+ transport systems, permease components; ABC 3 transport family | Mn/Zn | 11 | 10 | 11 |
| *PA14_33550* | ABC transporter ATP-binding protein | Transport of small molecules // ATP binding; ATPase activity | ABC transporters // ATP-binding cassette, ABC transporter-type domain profile | Mn/Zn | 8 | 7 | 7 |
| *PA14_33560* | Adhesion protein | Motility and attachment // Cell adhesion; metal ion transport; metal ion binding | ABC transporters // Adhesin B signature; periplasmic solute binding protein family | Mn/Zn | 6 | 7 | 7 |
| *PA14_15435^c^* | Hypothetical protein | Hypothetical, unclassified, unknown; motility and attachment // No GO terms listed | // Putative diguanylate phosphodiesterase | Hg | -7 | -4 | -4 |
| *merA* | MerA | Adaptation, protection // Oxidation-reduction process; detoxification of mercury ion; cell redox hemeostasis; metal ion transport; mercury ion binding; oxidoreductase activity, acting on a sulfur group of donors, NAD(P) as acceptor; Flavin adenine dinucleotide binding; NADP binding; mercury (II) reductase activity | Mercury binding and transport // Heavy-metal-associated domain; mercuric reductase class II signature | Hg | -2 | -2 | -3 |
| *merP* | MerP | Adaptation, protection // Response to mercury ion; mercury ion transport; periplasmic space; mercury ion binding; mercury ion transmembrane transporter activity | // Mercuric transport protein periplasmic component; mercury scavenger protein signature; heavy-metal-associated domain | Hg | -2 | -2 | -2 |
| *merR* | Transcriptional regulator MerR | Transcriptional regulators // Regulation of transcription, DNA-templated; response to mercury ion; mercury ion binding; DNA binding | // Hg(II)-responsive transcriptional regulator; MerR-type HTH domain signature | Hg | -5 | -5 | -5 |
| *modA* | Molybdate-binding periplasmic protein precursor ModA | Transport of small molecules // Molybdate ion transport; outer membrane-bounded periplasmic space; molybdate transmembrane-transporting ATPase activity | ABC transporters // Molybdenum ABC transporter, periplasmic binding protein | Mb | -3 | -3 | -2 |
| *kdpE^c^* | Two-component response regulator KdpE | Two-component regulatory systems; transcriptional regulators // Regulation of transcription, DNA-templated; phosphorelay signal transduction system; DNA binding | Two-component system // Signal transduction response regulator, receiver domain; winged helix-turn-helix DNA-binding domain | K | -2 | -2 | -2 |
| *kdpC* | Potassium-transporting ATPase subunit C | Transport of small molecules // Potassium ion transport; integral component of membrane; potassium-transporting ATPase activity | Two-component system // Potassium-transporting ATPase C chain | K | -39 | -7 | -2 |
| *arsB^c^* | Arsenite-antimonite efflux pump ArsB | Transport of small molecules // Arsenite transport; integral component of membrane; arsenite transmembrane transporter activity | // Arsenical pump membrane protein signature | As | -3 | -3 | -3 |
| *arsR* | Arsenic resistance transcriptional regulator | Transcriptional regulators // Regulation of transcription, DNA-templated; sequence-specific DNA binding transcription factor activity | // ArsR-type HTH domain profile; winged helix-turn-helix DNA-binding domain | As | -5 | -4 | -32 |

^a^Product names, functional classification(s), gene ontology terms, pathways, and functional predictions for PA14 genes were obtained from the MGH-ParaBioSys:NHLBI Program for Genomic Applications, Massachusetts General Hospital and Harvard Medical School, Boston, MA (<http://pga.mgh.harvard.edu>; accessed 10Nov2015) [1] made available by the *Pseudomonas Genome Database* (<http://www.pseudomonas.com/>; accessed 10Nov2015) [2].

^b^Gene expression within PA14 grown in whole blood from the three severely burned patients (Pt) was compared with expression when PA14 was grown in whole blood from a healthy volunteer.

^c^Genes found in operons are color-coded, with related genes in close proximity highlighted a lighter color.

**References**

1. Lee DG, Urbach JM, Liberati NT, Feinbaum RL, Miyata S, Diggins LT, et al. (2006) Genomic analysis reveals that *Pseudomonas aeruginosa* virulence is combinatorial. Genome Biol 7: R90.

2. Winsor GL, Lam DK, Fleming L, Lo R, Whiteside MD, Yu NY, et al. (2011) *Pseudomonas* Genome Database: improved comparative analysis and population genomics capability for *Pseudomonas* genomes. Nucleic Acids Res 39: D596-600.
