# Supplementary material for: Major Transcriptome Changes Accompany the Growth of Pseudomonas aeruginosa in Blood from Patients with Severe Thermal Injuries
Source: PLoS One. 2016 Mar 2;11(3):e0149229. doi: 10.1371/journal.pone.0149229 (PMC4774932; doi:10.1371/journal.pone.0149229)
Supplement: S3 Table — Gene expression within PA14 grown in whole blood from the three severely burned patients was compared with expression when PA14 was grown in whole blood from a healthy volunteer. Product names, functional classification(s), gene ontology terms, pathways, and functional predictions for PA14 genes were obtained from the MGH-ParaBioSys:NHLBI Program for Genomic Applications, Massachusetts General Hospital and Harvard Medical School, Boston, MA (http://pga.mgh.harvard.edu; accessed 10Nov2015) [45] made available by the Pseudomonas Genome Database (http://www.pseudomonas.com/; accessed 10Nov2015) [44]. (DOCX) [file pone.0149229.s007.docx]

**S3 Table. ABC-transporter genes whose expression is enhanced.**

| **Gene/ORF** | **Product^a^** | **Functional classification(s) // Gene ontology terms^a^** | **Pathways // Functional predictions^a^** | **Pt 1** | **Pt 2** | **Pt 3** |
| --- | --- | --- | --- | --- | --- | --- |
| *PA14_00850^c^* | Hypothetical protein | Transport of small molecules; membrane proteins; protein secretion/export apparatus // Membrane | // Permease FtsX-like | 21 | 18 | 20 |
| *PA14_00860* | ABC transporter ATP-binding protein | Protein secretion/export apparatus; transport of small molecules // ATPase activity; ATP binding | // ATP-binding cassette; ABC transporter-type domain profile; ATPases associated with a variety of cellular activities | 5 | 9 | 11 |
| *ppkA* | Serine/threonine protein kinase PpkA | Adaptation, protection; protein secretion/export apparatus; translation, post-translational modification, degradation // Positive-regulation of protein secretion; protein phosphorylation; protein serine/threonine kinase activity; transferase activity; transferring phosphorus-containing groups; ATP binding | Bacterial secretion system // Serine/threonine-protein kinase, active site | 4 | 4 | 4 |
| *PA14_00890* | Phosphoprotein phosphatase | Putative enzymes; protein secretion/export apparatus // Negative-regulation of protein secretion; protein serine/ threonine phosphatase activity; catalytic activity | Bacterial secretion system // Serine/threonine phosphatases, family 2C, catalytic domain | 5 | 4 | 4 |
| *PA14_00900* | Hypothetical protein | Hypothetical, unclassified, unknown; protein secretion/export apparatus // No GO terms assigned | // Type VI secretion-associated protein, BMA_A0400 family | 3 | 3 | 3 |
| *PA14_00910* | Hypothetical protein | Hypothetical, unclassified, unknown // No GO terms assigned | Bacterial secretion system // Type VI secretion protein IcmF | 3 | 3 | 3 |
| *PA14_04250* | ABC transporter ATP-binding protein | Transport of small molecules // Polyamine transport; ATP-binding cassette (ABC) transporter complex; hydrolase activity, acting on acid anhydrides, catalyzing transmembrane movement of substances; polyamine-transporting ATPase activity; ATP binding | ABC transporters // Spermidine/putrescine ABC transporter, ATP-binding subunit; AAA+ ATPase domain | 6 | 6 | 4 |
| *PA14_09300^c^* | ABC transporter ATP-binding protein | Transport of small molecules // Transmembrane transport; integral component of membrane; ATP binding; ATPase activity coupled to transmembrane movement of substances | // ABC transporter type 1, transmembrane domain; AAA+ ATPase domain | 9 | 9 | 9 |
| *PA14_09320* | ABC transporter ATP-binding protein | Transport of small molecules // Transmembrane transport; integral component of membrane; ATP binding; ATPase activity coupled to transmembrane movement of substances   \|  \|  \| \| --- \| --- \| | // ABC transporter type 1, transmembrane domain; AAA+ ATPase domain | 7 | 6 | 7 |
| *PA14_13580* | ABC transporter ATP-binding protein | Transport of small molecules // Glycine betaine transport; membrane; ATP binding; ATPase activity; adenyl nucleotide binding | ABC transporters // Glycine betaine transport ATP-binding subunit; AAA+ ATPase domain | 2 | 2 | 2 |
| *PA14_14380* | ABC transporter permease | Transport of small molecules // Transport; membrane; transporter activity | // Branched-chain amino acid transport system/permease component | 3 | 3 | 3 |
| *PA14_26400* | ABC transporter ATP-binding protein | Transport of small molecules // ATPase activity; ATP binding | ABC transporters // ATP-binding cassette, ABC transporter-type domain profile; AAA+ ATPase domain | 2 | 2 | 1 |
| *PA14_39130* | ABC transporter ATP-binding protein | Transport of small molecules // ATPase activity; ATP binding | // ATP-binding cassette, ABC transporter-type domain profile; AAA+ ATPase domain | 5 | 5 | 5 |
| *PA14_53150* | ABC transporter ATP-binding protein/permease | Transport of small molecules; membrane proteins // Transmembrane transport; integral component of membrane; ATP binding; ATPase activity coupled to transmembrane movement of substances | ABC transporters // ATP-binding cassette, ABC transporter-type domain profile; AAA+ ATPase domain | 2 | 2 | 2 |
| *PA14_55000^c^* | ABC transporter periplasmic protein | Transport of small molecules // Binding | ABC transporters // Iron siderophore/cobalamin periplasmic-binding domain profile | 2 | 2 | 2 |
| *PA14_55030* | ABC transporter permease | Transport of small molecules // Membrane; transporter activity | ABC transporters // FecCD transporter family, permease | 3 | 3 | 3 |
| *dppA3* | Dipeptide ABC transporter substrate-binding protein DppA3 | Transport of small molecules // Dipeptide transport; transporter activity | ABC transporters; bacterial chemotaxis // Bacterial extracellular solute-binding protein, family 5   \|  \|  \| \| --- \| --- \| | 5 | 5 | 5 |
| *PA14_60770^c^* | Outer membrane protein | Membrane proteins; hypothetical, unclassified, unknown // Transport; transporter activity | // Outer membrane efflux protein | 4 | 4 | 3 |
| *PA14_60780* | ABC transporter permease | Transport of small molecules; membrane proteins // Membrane | // FtsX-like permease family; MacB-like periplasmic core domain | 3 | 3 | 2 |
| *hmuV^c^* | Hemin importer ATP-binding subunit | Transport of small molecules // Heme transport; membrane; heme-transporting ATPase activity; ATP binding | ABC transporters; sulfur metabolism // Hemin import ATP-binding protein HmuV family profile; AAA+ ATPase domain | 2 | 2 | 2 |
| *PA14_62290* | ABC transporter permease | Transport of small molecules // Membrane; transporter activity | ABC transporters // FecCD transport family | 3 | 3 | 3 |
| *PA14_68080* | ABC transporter permease | Transport of small molecules; membrane proteins // Transport; integral component of membrane; transporter activity | // Amino acid ABC transporter, permease protein, 3-TM domain, His/Glu/Gln/Arg/opine family | 7 | 5 | 6 |
| *PA14_69060^c^* | ABC transporter permease | Transport of small molecules // Transport; membrane; ATP binding; ATPase activity coupled to transmembrane movement of substances | // ABC transporter integral membrane type-2 domain profile | 5 | 4 | 4 |
| *PA14_69070* | ABC transporter ATP-binding protein/permease | Transport of small molecules // Transport; membrane; ATP binding; ATPase activity coupled to transmembrane movement of substances | // ATP-binding cassette, ABC transporter-type domain profile; AAA+ ATPase domain; ABC transporter integral membrane type-2 domain profile | 4 | 4 | 4 |
| *PA14_69090* | Hypothetical protein | Membrane proteins // Transmembrane transport; membrane | // RND efflux pump, membrane fusion protein; HlyD family secretion protein | 3 | 3 | 3 |
| *PA14_69340* | ABC transporter ATP-binding protein | Transport of small molecules // ATP binding; ATPase activity | // ATP-binding cassette, ABC transporter-type domain profile; AAA+ ATPase domain | 3 | 2 | 2 |
| *metN^c^* | DL-methionine transporter ATP-binding subunit | Transport of small molecules // ATP binding; ATPase activity | ABC transporters; sulfur metabolism // Methionine import ATP-binding protein MetN family profile; ATP-binding cassette, ABC transporter-type domain profile; AAA+ ATPase domain | 2 | 2 | 2 |
| *PA14_72630* | ABC transporter permease | Transport of small molecules; membrane proteins // Transport; membrane | ABC transporters // ABC transporter integral membrane type-1 domain profile; binding-protein-dependent transport system inner membrane component | 3 | 3 | 3 |
| *gltK* | ABC transporter ATP-binding protein | Transport of small molecules // Transport; ATP-binding cassette (ABC) transporter complex; ATP binding; transporter activity; ATPase activity; hydrolase activity, acting on acid anhydrides, catalyzing transmembrane movement of substances | ABC transporters // ATP-binding cassette, ABC transporter-type domain profile; AAA+ ATPase domain; transport-associated OB, type 1 | 3 | 2 | 3 |

^a^Product names, functional classification(s), gene ontology terms, pathways, and functional predictions for PA14 genes were obtained from the MGH-ParaBioSys:NHLBI Program for Genomic Applications, Massachusetts General Hospital and Harvard Medical School, Boston, MA (<http://pga.mgh.harvard.edu>; accessed 10Nov2015) [1] made available by the *Pseudomonas Genome Database* (<http://www.pseudomonas.com/>; accessed 10Nov2015) [2].

^b^Gene expression within PA14 grown in whole blood from the three severely burned patients (Pt) was compared with expression when PA14 was grown in whole blood from a healthy volunteer.

^c^Genes found in operons are color-coded, with related genes in close proximity highlighted a lighter color.

**References**

1. Lee DG, Urbach JM, Liberati NT, Feinbaum RL, Miyata S, Diggins LT, et al. (2006) Genomic analysis reveals that *Pseudomonas aeruginosa* virulence is combinatorial. Genome Biol 7: R90.

2. Winsor GL, Lam DK, Fleming L, Lo R, Whiteside MD, Yu NY, et al. (2011) *Pseudomonas* Genome Database: improved comparative analysis and population genomics capability for *Pseudomonas* genomes. Nucleic Acids Res 39: D596-600.
