# Supplementary material for: Major Transcriptome Changes Accompany the Growth of Pseudomonas aeruginosa in Blood from Patients with Severe Thermal Injuries
Source: PLoS One. 2016 Mar 2;11(3):e0149229. doi: 10.1371/journal.pone.0149229 (PMC4774932; doi:10.1371/journal.pone.0149229)
Supplement: S4 Table — Expression of genes within PA14 that was grown in whole blood from the three severely burned patients was compared with the expression when PA14 was grown in whole blood from a healthy volunteer. Product names, functional classification(s), gene ontology terms, pathways, and functional predictions for PA14 genes were obtained from the MGH-ParaBioSys:NHLBI Program for Genomic Applications, Massachusetts General Hospital and Harvard Medical School, Boston, MA (http://pga.mgh.harvard.edu; accessed 10Nov2015) [45] made available by the Pseudomonas Genome Database (http://www.pseudomonas.com/; accessed 10Nov2015) [44]. (DOCX) [file pone.0149229.s008.docx]

**S4 Table.** **ABC-transport genes whose expression is significantly reduced.**

| **Gene/ORF** | **Product^a^** | **Functional classification(s) // Gene ontology terms^a^** | **Pathways // Functional predictions^a^** | **Pt 1** | | **Pt 2** | **Pt 3** |
| --- | --- | --- | --- | --- | --- | --- | --- |
| *PA14_01670* | ABC transporter ATP-binding protein | Transport of small molecules // ATPase activity, ATP binding | // ATP-binding cassette, ABC transporter-type domain profile; AAA+ ATPase domain | -3^b^ | | -2 | -3 |
| *atsA^c^* | Arylsulfatase | Carbon compound catabolism; Central intermediary metabolism // Metabolic process; phosphoric diester hydrolase activity; arylsulfatase activity; catalytic activity; sulfuric ester hydrolase activity | // Sulfatase | -123 | | -85 | -173 |
| *PA14_02330* | [ABC transporter ATP-binding protein](http://www.pseudomonas.com/feature/show?id=1651207) | Transport of small molecules // ATPase activity, ATP binding | ABC transporters; sulfur metabolism // ATP-binding cassette, ABC transporter-type domain profile | -163 | | -111 | -125 |
| *PA14_02340* | ABC transporter permease | Transport of small molecules // transport; membrane | ABC transporters; sulfur metabolism // ABC transporter integral membrane type-1 domain profile. | -70 | | -158 | -31 |
| *PA14_02360* | ABC transporter substrate-binding protein | Transport of small molecules // | ABC transporters; sulfur metabolism // NMT1/THI5 like | -36 | | -21 | -29 |
| *PA14_02370* | Porin | Membrane proteins // transport; integral component of membrane; porin activity | // Outer membrane porin, OprD family | -21 | | -9 | -14 |
| *PA14_04230^c^* | ABC transporter permease | Transport of small molecules; nembrane proteins // Transport; membrane | ABC transporters // ABC transporter integral membrane type-1 domain profile; binding-protein-dependent transport system inner membrane component | -3 | | -3 | -3 |
| *PA14_04240* | ABC transporter permease | Transport of small molecules; membrane proteins // Transport; membrane | ABC transporters // ABC transporter integral membrane type-1 domain profile; binding-protein-dependent transport system inner membrane component | -2 | | -2 | -3 |
| *PA14_07860^c^* | ABC transporter ATP-binding protein | Transport of small molecules // Polyamine transport; ATP-binding cassette (ABC) transporter complex; polyamine-transporting ATPase activity; ATP binding; hydrolase activity, acting on acid anhydrides, catalyzing transmembrane movement of substances | ABC transporters // ATP-binding cassette, ABC transporter-type domain profile; spermidine/putrescine ABC transporter, ATP-binding subunit | -5 | | -4 | -2 |
| *PA14_07870* | ABC transporter substrate-binding protein | Transport of small molecules // | // Bacterial extracellular solute-binding protein | -5 | | -4 | -7 |
| *PA14_07890* | ABC transporter permease | Transport of small molecules; membrane proteins // Transport; membrane | ABC transporters // ABC transporter integral membrane type-1 domain profile; binding-protein-dependent transport system inner membrane component | -3 | | -2 | -3 |
| *PA14_11600^c^* | ABC transporter | Transport of small molecules // | // ABC-type uncharacterized transport system; intra-flagellar transport proteins involved in gliding motility | -3 | | -4 | -3 |
| *PA14_11620* | ABC transporter | Transport of small molecules // ATPase activity; ATP binding | // ATP-binding cassette, ABC transporter-type domain profile | -2 | | -3 | -3 |
| *PA14_12920^c^* | Taurine ABC transporter periplasmic protein | Transport of small molecules // Taurine transport; periplasmic space | ABC transporters; sulfur metabolism // Taurine ABC transporter, periplasmic binding protein TauA | -165 | | -149 | -201 |
| *PA14_12940* | Taurine ABC transporter ATP-binding protein | Transport of small molecules // Taurine transport; membrane; taurine-transporting ATPase activity; ATP binding | ABC transporters; sulfur metabolism // Taurine import ATP-binding protein TauB | -488 | | -330 | -275 |
| *PA14_12960* | Taurine ABC transporter permease | Transport of small molecules // Transport; membrane | ABC transporters; sulfur metabolism // ABC transporter integral membrane type-1 domain profile; Binding-protein-dependent transport system inner membrane component | -146 | | -72 | -82 |
| *tauD* | Taurine dioxygenase | Carbon compound catabolism // Oxidation-reduction process; oxidoreductase activity | Sulfur metabolism; taurine and hypotaurine metabolism // Taurine catabolism dioxygenase TauD, TfdA family | -248 | | -213 | -202 |
| *PA14_16880^c^* | ABC transporter permease | Transport of small molecules; membrane proteins // No GO terms listed | // ABC-2 family transporter protein | -2 | | -3 | -3 |
| *PA14_16890* | Auxiliary component of ABC transporter | Motility and attachment // No GO terms listed | // ABC-type uncharacterized transport system | -5 | | -4 | -4 |
| *PA14_16910* | Hypothetical protein | Hypothetical, unclassified, unknown // No GO terms listed | // Domain of unknown function DUF4340 | -2 | | -3 | -2 |
| *PA14_19500^c^* | Hypothetical protein | Transport of small molecules // Sulfur compound metabolic process; transport; membrane | ABC transporters; sulfur metabolism // Aliphatic sulfonates-binding protein | -580 | | -165 | -298 |
| *PA14_19510* | ABC transporter permease | Transport of small molecules // Transport; membrane | ABC transporters; sulfur metabolism // ABC transporter integral membrane type-1 domain profile; Binding-protein-dependent transport system inner membrane component | -179 | | -248 | -251 |
| *PA14_19520* | ABC transporter ATP-binding protein | Transport of small molecules // Transport; membrane; ATPase activity; ATP binding; transporter activity; hydrolase activity | ABC transporters; sulfur metabolism // Aliphatic sulfonates import ATP-binding protein SsuB | -137 | | -93 | -105 |
| *ssuD^c^* | Alkanesulfonate monooxygenase | Central intermediary metabolism // Oxidation-reduction process; oxidoreductase activity, acting on paired donors, with incorporation or reduction of molecular oxygen; alkanesulfonate monooxygenase activity | Sulfur metabolism // Alkanesulfonate monooxygenase, FMN-dependent | -471 | | -891 | -604 |
| *PA14_19570* | ABC transporter permease | Transport of small molecules // Transport; membrane | ABC transporters; sulfur metabolism // ABC transporter integral membrane type-1 domain profile; binding-protein-dependent transport system inner membrane component | -313 | | -145 | -171 |
| *ssuB* | Aliphatic sulfonates transport ATP-binding subunit | Transport of small molecules // Transport; membrane; ATPase activity; ATP binding; transporter activity; hydrolase activity | ABC transporters; sulfur metabolism // Aliphatic sulfonate import ATP-binding protein SsuB family | -41 | | -25 | -58 |
| *PA14_19590* | Molybdopterin-binding protein | Central intermediary metabolism // Transport; ATP-binding cassette (ABC) transporter complex; ATP binding; hydrolase activity, acting on acid anhydrides, catalyzing transmembrane movement of substances; molybdenum ion binding; transporter activity | // Molybdenum-pterin binding | -316 | | -340 | -308 |
| *PA14_21160^c^* | ABC transporter ATP-binding protein | Transport of small molecules // Organic phosphonate transport; membrane; organic phosphonate transmembrane-transporting ATPase activity | ABC transporters // Phosphonate import ATP-binding protein PhnC | -2 | | -2 | -2 |
| *PA14_21175* | Hypothetical protein | Transport of small molecules // Transmembrane transport; ATP-binding cassette (ABC) transporter complex | ABC transporters // Phosphate/phosphite/ phosphonate ABC transporter, periplasmic protein | -4 | | -3 | -3 |
| *PA14_21930^c^* | ABC transporter permease | Transport of small molecules; membrane proteins // Transport; membrane | // ABC transporter integral membrane type-1 domain profile; Binding-protein-dependent transport system inner membrane component | -3 | | -2 | -2 |
| *PA14_21940* | Hypothetical protein | Hypothetical, unclassified, unknown // metabolic process; catalytic activity | // Type I phosphodiesterase/ nucleotide pyrophosphatase/ phosphate transferase | -2 | | -3 | -3 |
| *PA14_21960* | Hypothetical protein | Transport of small molecules; hypothetical, unclassified, unknown // No GO terms listed | // Bacterial extracellular solute-binding protein | -5 | | -4 | -5 |
| *hisM^c^* | Histidine ABC transporter, inner membrane permease | Transport of small molecules // Transport; integral component of membrane; transporter activity | ABC transporters // Amino acid ABC transporter, permease protein, 3-TM domain, His/Glu/Gln/Arg/opine family | -6 | | -6 | -4 |
| *hisQ* | Histidine transport system permease | Transport of small molecules // Transport; integral component of membrane; transporter activity | ABC transporters // Amino acid ABC transporter, permease protein, 3-TM domain, His/Glu/Gln/Arg/opine family | -13 | | -5 | -3 |
| *hisJ* | Periplasmic histidine-binding protein HisJ | Transport of small molecules // Transport; transporter activity | ABC transporters // Bacterial extracellular solute-binding proteins, family 3 | -5 | | -2 | -5 |
| *PA14_26360* | ABC transporter permease | Membrane proteins // Membrane; transporter activity | ABC transporters // ABC transporter, permease protein; FecCD transport family | -2 | | -3 | -2 |
| *PA14_34260^c^* | ABC transporter permease | Transport of small molecules; membrane proteins // Transport; membrane | ABC transporters // ABC transporter type 1, transmembrane domain MetI-like | -27 | | -63 | -34 |
| *PA14_34270* | ABC transporter ATP-binding protein | Transport of small molecules // ATPase activity; ATP binding | ABC transporters // ABC transporter, methionine import, ATP-binding protein, MetN | -132 | | -150 | -215 |
| *PA14_34280* | Hypothetical protein | Transport of small molecules; hypothetical, unclassified, unknown // No GO terms listed | ABC transporters // Lipoprotein NlpA family | -192 | | -654 | -256 |
| *PA14_34290^c^* | DszA family monooxygenase | Central intermediary metabolism // Oxidation-reduction process; monooxygenase activity; oxidoreductase activity, acting on paired donors, with incorporation or reduction of molecular oxygen | // Nitrilotriacetate monooxygenase component A/pristinamycin IIA synthase subunit A | -93 | | -316 | -160 |
| *PA14_34300* | DszC family monooxygenase | Putative enzymes // Oxidation-reduction process; metabolic process; flavin adenine dinucleotide binding; oxidoreductase activity, acting on the CH-CH group of donors; acyl-CoA dehydrogenase activity | // Sulfate-starvation-induced SfnB; acyl-CoA dehydrogenase/oxidase | -31 | | -58 | -58 |
| *PA14_34320* | DszC family monooxygenase | Putative enzymes // Oxidation-reduction process; metabolic process; flavin adenine dinucleotide binding; oxidoreductase activity, acting on the CH-CH group of donors; acyl-CoA dehydrogenase activity | // Sulfate-starvation-induced SfnB; acyl-CoA dehydrogenase/oxidase | -49 | | -74 | -114 |
| *mtlD^c^* | mannitol dehydrogenase | Carbon compound catabolism // Mannitol metabolic process; oxidation-reduction process; coenzyme binding; oxidoreductase activity, acting on the CH-OH group of donors, NAD or NADP as acceptor; catalytic activity | Fructose and mannose metabolism // Mannitol dehydrogenase signature | -4 | | -2 | -2 |
| *PA14_34370* | ABC maltose/ mannitol transporter ATP-binding protein | Transport of small molecules // Transport; ATP-binding cassette (ABC) transporter complex; hydrolase activity, acting on acid anhydrides, catalyzing transmembrane movement of substances; ATP binding; transporter activity; ATPase activity | ABC transporters // ATP-binding cassette, ABC transporter-type domain profile. | -4 | | -4 | -9 |
| *PA14_34410* | Binding-protein-dependent maltose/mannitol transport protein | Transport of small molecules // Transport; membrane | ABC transporters // ABC transporter type 1; transmembrane domain MetI-like | -5 | | -3 | -4 |
| *PA14_34420* | Maltose/mannitol ABC transporter substrate-binding protein | Transport of small molecules // Transport; transporter activity | ABC transporters // Bacterial extracellular solute-binding protein | -4 | | -4 | -6 |
| *mtlR* | transcriptional regulator MtlR | Transcriptional regulators // Regulation of transcription, DNA-templated; sequence-specific DNA binding transcription factor activity | // DNA binding HTH domain, AraC-type | -3 | | -2 | -2 |
| *PA14_34460^c^* | Hypothetical protein | Adaptation, protection // Oxidation-reduction process; peroxiredoxin activity | Glutathione metabolism // Carboxymucono-lactone decarboxylase family; Alkylhydroperoxidase AhpD family core domain | -49 | | -64 | -52 |
| *PA14_34490* | Hypothetical protein | Hypothetical, unclassified, unknown; fatty acid and phospholipid metabolism // Metabolic process; oxidation-reduction process; acyl-CoA dehydrogenase activity; oxidoreductase activity, acting on the CH-CH group of donors; flavin adenine dinucleotide binding | // Acyl-CoA dehydrogenase, N-terminal domain | -15 | | -17 | -18 |
| *PA14_34500* | ABC transporter ATP-binding protein | Transport of small molecules // ATPase activity; ATP binding  ABC-type nitrate/sulfonate/bicarbonate transport system, ATPase component | // ATP-binding cassette, ABC transporter-type domain profile | -16 | | -14 | -11 |
| *PA14_34510* | Hypothetical protein | Transport of small molecules // Cellular aromatic compound metabolic process; ferrous iron binding; oxidoreductase activity  ABC-type nitrate/sulfonate/ bicarbonate transport systems, periplasmic components | // Twin-arginine translocation pathway, signal sequence; estradiol ring-cleavage dioxygenase, class III enzyme, subunit B | -17 | -38 | | -18 |
| *PA14_34520* | ABC transporter permease | Transport of small molecules // transport; membrane | // ABC transporter type 1, transmembrane domain MetI-like | -26 | -24 | | -35 |
| *PA14_34750^c^* | Taurine catabolism dioxygenase | Central intermediary metabolism // Oxidation-reduction process; oxidoreductase activity; probable taurine catabolism dioxygenase | Sulfur metabolism; taurine and hypotaurine metabolism // Taurine catabolism dioxygenase TauD/TfdA | -992 | -1126 | | -1394 |
| *PA14_34770* | ABC transporter substrate-binding protein | Transport of small molecules // No GO terms listed | // NMT 1-like family | -415 | -490 | | -664 |
| *PA14_34780* | ABC transporter ATP-binding protein | Transport of small molecules // ATPase activity; ATP binding  ABC-type nitrate/sulfonate/ bicarbonate transport system, ATPase component | // ATP-binding cassette, ABC transporter-type domain profile | -1015 | -543 | | -544 |
| *PA14_34790* | ABC transporter permease | Transport of small molecules // Transport; membrane | // ABC transporter type 1, transmembrane domain MetI-like | -97 | -126 | | -273 |
| *PA14_34800* | Amino acid transporter LysE | Transport of small molecules // Amino acid transport; membrane | // Lysine-type exporter protein (LYSE/ YGGA) | -5 | -5 | | -6 |
| *PA14_36200* | ABC transporter ATP-binding protein | Transport of small molecules //  Transport; transporter activity | // Bacterial periplasmic substrate-binding proteins, family 3 | -4 | -4 | | -4 |
| *PA14_37840^c^* | ABC transporter ATP-binding protein | Transport of small molecules // Peptide transport; ATP binding; ATPase activity; nucleotide binding | ABC transporters // ATP-binding cassette, ABC transporter-type domain profile; oligopeptide/dipeptide ABC transporter, C-terminal | -15 | -20 | | -12 |
| *PA14_37850* | ABC transporter permease | Transport of small molecules // Transport; membrane | ABC transporters // ABC transporter type 1, transmembrane domain MetI-like | -90 | -27 | | -33 |
| *PA14_37870* | ABC transporter permease | Transport of small molecules // Transport; membrane | ABC transporters // ABC transporter type 1, transmembrane domain MetI-like | -11 | -20 | | -19 |
| *PA14_37880* | ABC transporter substrate-binding protein | Transport of small molecules // Transporter; transporter activity | ABC transporters // Bacterial extracellular solute-binding protein, family 5 | -33 | -35 | | -152 |
| *PA14_37900* | TonB-dependent receptor | Membrane proteins; hypothetical, unclassified, unknown // Transport; membrane; receptor activity; transporter activity | // TonB-dependent receptor plug domain | -17 | -26 | | -38 |
| *PA14_46910* | ABC transporter substrate-binding protein | Transport of small molecules // Transporter; transporter activity | ABC transporters; two-component system // Bacterial extracellular solute-binding proteins, family 3 | -5 | -5 | | -5 |
| *PA14_46920^c^* | ABC transporter permease | Transport of small molecules; membrane proteins // Transport; integral component of membrane ; transporter activity | ABC transporters; two-component system // Amino acid ABC transporter, permease protein, 3-TM domain, His/Glu/Gln/Arg/opine family | -2 | -2 | | -2 |
| *PA14_46930* | ABC transporter permease | Transport of small molecules; membrane proteins // Transport; integral component of membrane ; transporter activity | ABC transporters; two-component system // Amino acid ABC transporter, permease protein, 3-TM domain, His/Glu/Gln/Arg/opine family; ABC transporter type 1, transmembrane domain MetI-like | -2 | -3 | | -3 |
| *PA14_46950* | ABC transporter ATP-binding protein | Transport of small molecules // ATP binding; ATPase activity | ABC transporters; two-component system //  ATP-binding cassette, ABC transporter-type domain profile | -4 | -4 | | -4 |
| *PA14_47920^c^* | ABC transporter substrate-binding protein | Transport of small molecules // Transporter; transporter activity | ABC transporters // Bacterial extracellular solute-binding proteins, family 3 | -3 | -6 | | -5 |
| *PA14_47930* | Hypothetical protein | Hypothetical, unclassified, unknown // Phosphorylation; metabolic process; transferase activity, transferring phosphorus-containing groups | Valine, leucine and isoleucine biosynthesis; L-leucine biosynthesis // Aconitase/3-isopropylmalate dehydratase, swivel and other domains | -4 | -4 | | -3 |
| *PA14_47950* | ABC transporter permease | Transport of small molecules // transport; integral component of membrane; transporter activity | ABC transporters; two-component system // Amino acid ABC transporter, permease protein, 3-TM domain, His/Glu/Gln/Arg/opine family; ABC transporter type 1, transmembrane domain MetI-like | -2 | -3 | | -2 |
| *PA14_47970* | Hypothetical protein | Central intermediary metabolism // proline racemase activity | // Proline racemase | -3 | -3 | | -4 |
| *PA14_48450^c^* | Peptidylarginine deiminase | Putative enzymes // Putrescine biosynthetic process; protein-arginine deiminase activity | Metabolic pathways; arginine and proline metabolism // Twin-arginine translocation pathway, signal sequence; *Porphyromonas*-type peptidylarginine deiminase | -3 | -2 | | -2 |
| *PA14_48460* | Polyamine ABC transporter substrate-binding protein | Transport of small molecules // Polyamine transport; periplasmic space; polyamine binding | ABC transporters // Bacterial periplasmic spermidine/putrescine-binding protein | -5 | -7 | | -2 |
| *PA14_48470* | N-carbamoyl-putrescine amidohydrolase | Amino acid biosynthesis and metabolism // Nitrogen compound metabolic process; polyamine biosynthetic process; hydrolase activity, acting on carbon-nitrogen (but not peptide) bonds; N-carbamoylputrescine amidase activity | Metabolic pathways; arginine and proline metabolism; putrescine biosynthesis via agmatine pathway // N-carbamoylputrescine amidase; carbon-nitrogen hydrolase | -6 | -5 | | -3 |
| *PA14_48490* | Peptidylarginine deiminase | Amino acid biosynthesis and metabolism // Putrescine biosynthetic process; protein-arginine deiminase activity | Metabolic pathways; arginine and proline metabolism // *Porphyromonas*-type peptidyl-arginine deiminase | -4 | -2 | | -3 |
| *PA14_64290* | ABC transporter permease | Transport of small molecules; membrane proteins // transport; membrane; transporter activity | ABC transporters // ABC transporter, urea, permease protein, UrtC | -7 | -2 | | -2 |
| *PA14_64310* | ABC transporter ATP-binding protein | Transport of small molecules // ATP binding; ATPase activity | ABC transporters // ATP-binding cassette, ABC transporter-type domain profile; ABC transporter, urea, ATP-binding protein, UrtE | -4 | -3 | | -6 |
| *PA14_64890* | Branched-chain amino acid ABC transporter permease | Transport of small molecules; membrane proteins // transport; membrane; transporter activity | ABC transporters // Branched-chain amino acid transport system / permease component | -6 | -4 | | -5 |
| *PA14_67130^c^* | ABC transporter substrate-binding protein | Transport of small molecules; membrane proteins // transport; membrane; transporter activity | ABC transporters; two-component system // Bacterial extracellular solute-binding proteins, family 3 | -2 | -3 | | -3 |
| *PA14_67140* | Hypothetical protein | Translation, post-translational modification, degradation // No GO terms listed | // Endoribonuclease L-PSP/chorismate mutase-like | -2 | -2 | | -3 |
| *hutG^c^* | N-formylgluta-mate amido-hydrolase | Amino acid biosynthesis and metabolism // Peptidoglycan catabolic process; N-acetylmura-moyl-L-alanine amidase activity | Metabolic pathways; histidine metabolism // N-formylglutamate deformylase; cell wall hydrolase/autolysin, catalytic | -3 | -3 | | -3 |
| *PA14_67270* | ABC transporter ATP-binding protein | Transport of small molecules // Glycine betaine transport; membrane; ATP binding; ATPase activity | ABC transporters // ATP-binding cassette, ABC transporter-type domain profile; glycine betaine transport ATP-binding subunit | -2 | -3 | | -3 |
| *PA14_67370^c^* | Hypothetical protein | Transport of small molecules // | // ABC transporter, phosphonate, periplasmic substrate-binding protein | -3 | -3 | | -3 |
| *PA14_67380* | Fatty acid desaturase | Fatty acid and phospholipid metabolism // Lipid metabolic process | // Fatty acid desaturase, type 1 | -4 | -5 | | -7 |
| *PA14_67400* | ABC transporter substrate-binding protein | Transport of small molecules // Transport; transporter activity | ABC transporters // ABC-type glycine betaine transport system, substrate-binding domain | -4 | -4 | | -4 |

^a^Product names, functional classification(s), gene ontology terms, pathways, and functional predictions for PA14 genes were obtained from the MGH-ParaBioSys:NHLBI Program for Genomic Applications, Massachusetts General Hospital and Harvard Medical School, Boston, MA (<http://pga.mgh.harvard.edu>; accessed 10Nov2015) [1] made available by the *Pseudomonas Genome Database* (<http://www.pseudomonas.com/>; accessed 10Nov2015) [2].

^b^Gene expression within PA14 grown in whole blood from the three severely burned patients (Pt) was compared with expression when PA14 was grown in whole blood from a healthy volunteer.

^c^Genes found in operons are color-coded, with related genes in close proximity highlighted a lighter color.

**References**

1. Lee DG, Urbach JM, Liberati NT, Feinbaum RL, Miyata S, Diggins LT, et al. (2006) Genomic analysis reveals that *Pseudomonas aeruginosa* virulence is combinatorial. Genome Biol 7: R90.

2. Winsor GL, Lam DK, Fleming L, Lo R, Whiteside MD, Yu NY, et al. (2011) *Pseudomonas* Genome Database: improved comparative analysis and population genomics capability for *Pseudomonas* genomes. Nucleic Acids Res 39: D596-600.
