# Supplementary material for: Major Transcriptome Changes Accompany the Growth of Pseudomonas aeruginosa in Blood from Patients with Severe Thermal Injuries
Source: PLoS One. 2016 Mar 2;11(3):e0149229. doi: 10.1371/journal.pone.0149229 (PMC4774932; doi:10.1371/journal.pone.0149229)
Supplement: S5 Table — Expression of genes within PA14 that was grown in whole blood from the three severely burned patients was compared with the expression when PA14 was grown in whole blood from a healthy volunteer. Product names, functional classification(s), gene ontology terms, pathways, and functional predictions for PA14 genes were obtained from the MGH-ParaBioSys:NHLBI Program for Genomic Applications, Massachusetts General Hospital and Harvard Medical School, Boston, MA (http://pga.mgh.harvard.edu; accessed 10Nov2015) [45] made available by the Pseudomonas Genome Database (http://www.pseudomonas.com/; accessed 10Nov2015) [44]. (DOCX) [file pone.0149229.s009.docx]

**S5 Table. TonB-related genes that are differentially regulated.**

| **Gene/ORF** | **Product^a^** | **Functional classification(s) // Gene ontology terms^a^** | **Pathways // Functional predictions^a^** | **Pt 1** | **Pt 2** | **Pt 3** |
| --- | --- | --- | --- | --- | --- | --- |
| *tonB2*^c^ | Hypothetical protein | Membrane proteins // Siderophore transport; outer membrane-bounded periplasmic space; energy transducer activity; siderophore transmembrane transporter activity | // Gram-negative bacterial TonB protein signature | -36^b^ | -40 | -48 |
| *exbB1* |  | Transport of small molecules // Transport; membrane; protein transporter activity | // MotA/TolQ/ExbB proton channel family | -176 | -158 | -145 |
| *exbD1* | Transport protein ExbD | Transport of small molecules // Transport; membrane; protein transporter activity | // Biopolymer transport protein ExbD/TolR | -108 | -120 | -130 |
| *tonB* | TonB protein | Transport of small molecules // Cellular response to antibiotics; type IV pilus-dependent motility; high-affinity ferric iron transport; iron assimilation; siderophore transport; pathogenesis; bacterial-type flagellar swarming motility; single-species biofilm formation; outer membrane-bounded periplasmic space; energy transducer activity; siderophore transmembrane transporter activity | // Gram-negative bacterial TonB protein signature | -3 | -3 | -4 |
| *PA14_01870* | TonB-dependent receptor | Transport of small molecules // Siderophore transport; outer membrane; receptor activity; siderophore transmembrane transporter activity; iron ion binding | // Prokaryotic membrane lipoprotein attachment site profile; secretin and TonB N terminus short domain; TonB-dependent receptor plug domain; TonB-dependent siderophore receptor | -3 | -3 | -2 |
| *PA14_02410*^c^ | TonB-dependent receptor | Membrane proteins // transport; membrane; receptor activity; transporter activity | // TonB-dependent receptor plug domain; TonB-dependent receptor proteins signature 2 | -232 | -339 | -193 |
| *PA14_02420* | Hypothetical protein | Biosynthesis of cofactors, prosthetic groups and carriers // Oxidation-reduction process; oxidoreductase activity | Taurine and hypotaurine metabolism; sulfur metabolism // Taurine catabolism dioxygenase TauD, TfdA family | -398 | -752 | -510 |
| *PA14_02435* | Hypothetical protein | Putative enzymes // Oxidation-reduction process; oxidoreductase activity | // Taurine catabolism dioxygenase TauD, TfdA family | -463 | -351 | -291 |
| *PA14_10200* | TonB-dependent receptor protein | Membrane proteins // Transport; membrane; receptor activity; transporter activity | // TonB-dependent receptor plug domain | -3 | -4 | -3 |
| *PA14_37440*^c^ | MFS transporter | Transport of small molecules; membrane proteins // Transmembrane transport; integral component of membrane | // Major facilitator superfamily (MFS) profile | -48 | -27 | -18 |
| *PA14_37460* | Permease | Transport of small molecules // Transmembrane transport; integral component of membrane | // Major facilitator superfamily (MFS) profile | -60 | -56 | -64 |
| *PA14_37470* | Flavin-dependent oxidoreductase | Hypothetical, unclassified, unknown; energy metabolism // oxidation-reduction process; oxidoreductase activity, acting on paired donors with incorporation or reduction of molecular oxygen | // Luciferase-like monooxygenase | -185 | -84 | -104 |
| *PA14_37490* | TonB-dependent receptor | Transport of small molecules // Transport; outer membrane; receptor activity; transporter activity | // Prokaryotic membrane lipoprotein attachment site profile; secretin and TonB N terminus short domain; TonB-dependent receptor plug domain | -18 | -16 | -15 |
| *PA14_37730* | TonB dependent receptor | Transport of small molecules // Transport; membrane; receptor activity; transporter activity | // TonB-dependent receptor plug domain | -19 | -21 | -17 |
| *PA14_37840*^c^ | ABC transporter ATP-binding protein | Transport of small molecules // Peptide transport; ATP binding; ATPase activity; nucleotide binding | ABC transporters // ATP-binding cassette. ABC transporter-type domain profilea; AAA+ ATPase domain; oligopeptide/dipeptide ABC transporter | -15 | -20 | -12 |
| *PA14_37850* | ABC transporter permease | Transport of small molecules // Transport; membrane | ABC transporters // ABC transporter integral membrane type-1 domain profile; binding-protein-dependent transport system inner membrane component | -90 | -27 | -33 |
| *PA14_37870* | Peptide ABC transporter permease | Transport of small molecules // Transport; membrane | ABC transporters // ABC transporter integral membrane type-1 domain profile; binding-protein-dependent transport system inner membrane component | -11 | -20 | -19 |
| *PA14_37880* | ABC transporter substrate-binding protein | Transport of small molecules // Transport; transporter activity | ABC transporters // Bacterial extracellular solute-binding proteins, family 5 | -33 | -35 | -152 |
| *PA14_37900* | TonB-dependent receptor | Hypothetical, unclassified, unknown; membrane proteins // Transport; membrane; receptor activity; transporter activity | // TonB-dependent receptor plug domain | -17 | -26 | -38 |
| *PA14_39620*^c^ | Hypothetical protein | Hypothetical, unclassified, unknown // No GO terms listed | // Uncharacterized conserved protein (DUF2149) | -2 | -4 | -4 |
| *PA14_39630* | Hypothetical protein | Hypothetical, unclassified, unknown // No GO terms listed | // No characterization (orthologs are biopolymer transport proteins) | -5 | -2 | 1 |
| *PA14_39650* | TonB-dependent receptor | Transport of small molecules // Transport; membrane; receptor activity; transporter activity | // TonB-dependent receptor plug domain | -12 | -13 | -14 |
| *PA14_39820* | TonB-dependent receptor protein | Transport of small molecules // Siderophore transport; outer membrane; receptor activity; siderophore transmembrane transporter activity; iron ion binding | // Secretin and TonB N terminus short domain; TonB-dependent receptor plug domain; TonB-dependent siderophore receptor; TonB-dependent receptor proteins signature 2 | -2 | -2 | -2 |
| *PA14_05300* | TonB domain-containing protein | Membrane proteins; motility and attachment // transport | // Gram-negative bacterial TonB protein C-terminal | 2 | 2 | 2 |
| *PA14_34990* | TonB-dependent receptor | Transport of small molecules // Transport; membrane; receptor activity; transporter activity | // Prokaryotic membrane lipoprotein attachment site profile; TonB-dependent receptor plug domain | 3 | 3 | 3 |

^a^Product names, functional classification(s), gene ontology terms, pathways, and functional predictions for PA14 genes were obtained from the MGH-ParaBioSys:NHLBI Program for Genomic Applications, Massachusetts General Hospital and Harvard Medical School, Boston, MA (<http://pga.mgh.harvard.edu>; accessed 10Nov2015) [1] made available by the *Pseudomonas Genome Database* (<http://www.pseudomonas.com/>; accessed 10Nov2015) [2].

^b^Gene expression within PA14 grown in whole blood from the three severely burned patients (Pt) was compared with expression when PA14 was grown in whole blood from a healthy volunteer.

^c^Genes found in operons are color-coded.

**References**

1. Lee DG, Urbach JM, Liberati NT, Feinbaum RL, Miyata S, Diggins LT, et al. (2006) Genomic analysis reveals that *Pseudomonas aeruginosa* virulence is combinatorial. Genome Biol 7: R90.

2. Winsor GL, Lam DK, Fleming L, Lo R, Whiteside MD, Yu NY, et al. (2011) *Pseudomonas* Genome Database: improved comparative analysis and population genomics capability for *Pseudomonas* genomes. Nucleic Acids Res 39: D596-600.
