# Supplementary material for: Major Transcriptome Changes Accompany the Growth of Pseudomonas aeruginosa in Blood from Patients with Severe Thermal Injuries
Source: PLoS One. 2016 Mar 2;11(3):e0149229. doi: 10.1371/journal.pone.0149229 (PMC4774932; doi:10.1371/journal.pone.0149229)
Supplement: S6 Table — Expression of genes within PA14 that was grown in whole blood from the three severely burned patients was compared with the expression when PA14 was grown in whole blood from a healthy volunteer. Product names, functional classification(s), gene ontology terms, pathways, and functional predictions for PA14 genes were obtained from the MGH-ParaBioSys:NHLBI Program for Genomic Applications, Massachusetts General Hospital and Harvard Medical School, Boston, MA (http://pga.mgh.harvard.edu; accessed 10Nov2015) [45] made available by the Pseudomonas Genome Database (http://www.pseudomonas.com/; accessed 10Nov2015) [44]. (DOCX) [file pone.0149229.s010.docx]

**S6 Table. Phosphonate transport genes that are differentially regulated.**

| **Gene/ORF** | **Product^a^** | **Functional classification(s) // Gene ontology terms^a^** | **Pathways // Functional predictions^a^** | **Pt 1** | **Pt 2** | **Pt 3** |
| --- | --- | --- | --- | --- | --- | --- |
| *phnC^c^* | ABC phosphonate transporter ATP-binding protein | Transport of small molecule // Organic phosphonate transport; membrane; ATP binding; organic phosphonate transmembrane-transporting ATPase activity | ABC transporters // Phosphonate import ATP-binding protein PhnC; AAA+ ATPase domain | -262^b^ | -996 | -367 |
| *phnD* | Phosphonate ABC transporter substrate-binding protein | Transport of small molecule // Organic phosphonate transport; transmembrane transport; ATP-binding cassette (ABC) transporter complex; organic phosphonate transmembrane transporter activity | ABC transporters // Phosphonate ABC transporter, periplasmic substrate-binding protein | -792 | -904 | -856 |
| *phnE* | Phosphonate ABC transporter permease | Transport of small molecule // Organic phosphonate transport; regulation of apoptotic process; integral component of plasma membrane; organic phosphonate transmembrane transporter activity | ABC transporters // Phosphonate ABC transporter, permease protein PhnE/PtxC; ABC transporter integral membrane type-1 domain | -288 | -428 | -196 |
| *phnF* | Transcriptional regulator | Transcription regulators // Regulation of transcription, DNA-templated; DNA binding; sequence-specific DNA binding transcription factor activity | // Phonsphonate C-P lyase system, transcriptional regulator; winged HTH DNA-binding domain | -18 | -20 | -17 |
| *phnG* | Phosphonate metabolism protein PhnG | Transport of small molecules // Organic phosphonate transport; organic phosphonate metabolic process | Phosphonate and phosphinite metabolism // Phosphonate C-P lyase system protein PhnG | -10 | -14 | -15 |
| *phnH* | Carbon-phosphorus lyase complex subunit | Transport of small molecules // Organic phosphonate metabolic process | Phosphonate and phosphinite metabolism // Phosphonate C-P lyase system protein PhnG; bacterial phosphonate metabolism | -59 | -31 | -46 |
| *phnI* | Hypothetical protein | Transport of small molecules // Organic phosphonate metabolic process | Phosphonate and phosphinite metabolism // Bacterial phosphonate metabolism protein PhnI | -71 | -55 | -142 |
| *phnJ* | Hypothetical protein | Transport of small molecules // Alkylphosphonate transport; organic phosphonate catabolic process; lyase activity | Phosphonate and phosphinite metabolism // Phosphonate metabolism protein PhnJ | -61 | -72 | -92 |
| *phnK* | Phosphonate C-P lyase system protein PhnK | Transport of small molecules // Peptide transport; ATP binding; ATPase activity; nucleotide binding | // Phosphonate C-P lyase system protein PhnK; ATP-binding cassette, ABC transporter-type domain; AAA+ ATPase; oligopeptide/dipeptide transporter | -43 | -67 | -78 |
| *phnL* | Phosphonate ABC transporter ATPase | Transport of small molecules // ATP binding; ATPase activity | Phosphonate and phosphinite metabolism // Phosphonate C-P lyase system protein PhnL; ABC transporter-type domain; AAA+ ATPase | -90 | -82 | -39 |
| *phnM* | Phosphonate metabolism protein | Transport of small molecules // Organic phosphonate catabolic process; hydrolase activity, acting on carbon-nitrogen (but not peptide) bonds | Phosphonate and phosphinite metabolism // Phosphonate metabolism protein PhnM; aminohydrolase; metal-dependent hydrolase | -18 | -32 | -31 |
| *phnN* | Phosphonate transport ATP-binding protein | Transport of small molecules // 5-phosphoribose 1-diphosphate biosynthetic process; ribose 1,5-biphosphate phosphokinase activity; protein binding | Pentose phosphate pathway // Phosphonate metabolism protein/ 1,5-bisphosphokinase (PRPP-forming) PhnN | -12 | -11 | -8 |
| *phnP* | Carbon-phosphorus lyase complex accessory protein | Transport of small molecules // Organic phosphonate catabolic process; phosphoric diester hydrolase activity | Phosphonate and phosphinite metabolism // Phosphonate metabolism protein PhnP; metallo-beta-lactamase superfamily | -5 | -6 | -5 |
| *phnX^c^* | Phosphonoacet-aldehyde hydrolase | Carbon compound catabolism // metabolic process; organic phosphonate catabolic process; phosphonoacetaldehyde hydrolase activity | Phosphonate and phosphinite metabolism; microbial metabolism in diverse environments; metabolic pathways // Haloacid dehalogenase (HAD)-like hydrolase, family 1A, variant 3 | -3 | -2 | -2 |
| *phnW* | 2-aminoethyl-phosphonate-pyruvate transaminase | Carbon compound catabolism // metabolic process; organic phosphonate catabolic process; catalytic activity; 2-aminoethylphos-phonate-pyruvate transaminase activity; pyridoxal phosphate binding | Phosphonate and phosphinite metabolism; microbial metabolism in diverse environments; metabolic pathways // Pyroxidal phosphate-dependent transferase; aminotransferase class-V domain | -3 | -4 | -2 |

^a^Product names, functional classification(s), gene ontology terms, pathways, and functional predictions for PA14 genes were obtained from the MGH-ParaBioSys:NHLBI Program for Genomic Applications, Massachusetts General Hospital and Harvard Medical School, Boston, MA (<http://pga.mgh.harvard.edu>; accessed 10Nov2015) [1] made available by the *Pseudomonas Genome Database* (<http://www.pseudomonas.com/>; accessed 10Nov2015) [2].

^b^Gene expression within PA14 grown in whole blood from the three severely burned patients (Pt) was compared with expression when PA14 was grown in whole blood from a healthy volunteer.

^c^Genes found in operons are color-coded.

**References**

1. Lee DG, Urbach JM, Liberati NT, Feinbaum RL, Miyata S, Diggins LT, et al. (2006) Genomic analysis reveals that *Pseudomonas aeruginosa* virulence is combinatorial. Genome Biol 7: R90.

2. Winsor GL, Lam DK, Fleming L, Lo R, Whiteside MD, Yu NY, et al. (2011) *Pseudomonas* Genome Database: improved comparative analysis and population genomics capability for *Pseudomonas* genomes. Nucleic Acids Res 39: D596-600.
