# Supplementary material for: Major Transcriptome Changes Accompany the Growth of Pseudomonas aeruginosa in Blood from Patients with Severe Thermal Injuries
Source: PLoS One. 2016 Mar 2;11(3):e0149229. doi: 10.1371/journal.pone.0149229 (PMC4774932; doi:10.1371/journal.pone.0149229)
Supplement: S7 Table — Expression of genes within PA14 that was grown in whole blood from the three severely burned patients was compared with the expression when PA14 was grown in whole blood from a healthy volunteer. Product names, functional classification(s), gene ontology terms, pathways, and functional predictions for PA14 genes were obtained from the MGH-ParaBioSys:NHLBI Program for Genomic Applications, Massachusetts General Hospital and Harvard Medical School, Boston, MA (http://pga.mgh.harvard.edu; accessed 10Nov2015) [45] made available by the Pseudomonas Genome Database (http://www.pseudomonas.com/; accessed 10Nov2015) [44]. (DOCX) [file pone.0149229.s011.docx]

**S7 Table. Motility related genes that are differentially regulated.**

| **Gene/ORF** | **Product^a^** | **Functional classification(s) // Gene ontology (GO) terms^a^** | **Pathways // Functional predictions^a^** | **Pt 1** | **Pt 2** | **Pt 3** |
| --- | --- | --- | --- | --- | --- | --- |
| **Flagella** | | | | | | |
| *PA14_45520^c^* | Plasmid partitioning protein | Cell division // No GO terms assigned | Adenosylcobala-min biosynthesis // CobQ/CobB/MinD/ParA nucleotide binding domain | 3 | 3 | 3 |
| *motD* | Flagellar motor protein MotD | Motility and attachment; chemotaxis // Bacterial-type flagellum-dependent swarming motility; bacterial-type flagellum-dependent cell motility | Flagellar assembly; bacterial chemotaxis // Membrane MotB of proton-channel complex MotA/MotB; OmpA-like domain profile | 3 | 3 | 3 |
| *motC* | Flagellar motor protein | Motility and attachment; chemotaxis // Transport; bacterial-type flagellum-dependent swarming motility; bacterial-type flagellum-dependent cell motility; membrane; protein transporter activity | Two-component system; flagellar assembly; bacterial chemotaxis // MotA/TolQ/ExbB proton channel family | 3 | 3 | 3 |
| *PA14_45580* | Chemotaxis-specific methylesterase | Chemotaxis // chemotaxis; phosphorelay signal transduction system; cytoplasm; phosphorelay response regulator activity; protein-glutamate methylesterase activity | Two-component system; bacterial chemotaxis // Chemotaxis response regulator protein-glutamate methyl-esterase [CheB] | 4 | 4 | 3 |
| *PA14_45590* | Two-component sensor | Two-component regulatory systems // Chemotaxis; phosphorelay signal transduction system; phosphorylation; cytoplasm; phosphorelay sensor kinase activity; protein histidine kinase activity; signal transducer activity; transferase activity, transferring phosphorus-containing groups | Two-component system; bacterial chemotaxis // Histidine-containing phosphotransfer (Hpt) domain profile; histidine kinase-like ATPases; bacterial sensor protein C-terminal signature | 2 | 3 | 2 |
| *cheY* | Two-component response regulator CheY | Two-component regulatory systems; transcriptional regulators // Positive regulation of single-species biofilm formation; phosphorelay signal transduction system | Two-component system; bacterial chemotaxis // CheY-homologous receiver domain | -2 | -2 | -2 |
| *flhA* | Flagellar biosynthesis protein FlhA | Motility and attachment; chemotaxis; adapation, protection // Protein secretion; bacterial-type flagellum assembly; integral component of membrane | Flagellar assembly // Type III secretion system inner membrane A protein family signature | 2 | 2 | 2 |
| *PA14_45700^c^* | Hypothetical protein | Hypothetical, unclassified, unknown // No GO terms listed | // TLB18.3, Psb32 and MOLO-1 founding proteins of phosphatase | 3 | 3 | 3 |
| *PA14_45710* | Hypothetical protein | Hypothetical, unclassified, unknown // no GO terms listed | // TLB18.3, Psb32 and MOLO-1 founding proteins of phosphatase | 4 | 4 | 4 |
| *fliP* | Flagellar biosynthesis protein FliP | Motility and attachment; chemotaxis; adapation, protection // Protein secretion; membrane | Flagellar assembly // Flagellar transport protein FliP; type III secretion system inner membrane P protein family signature | 2 | 2 | 2 |
| *PA14_45830* | Hypothetical protein | Hypothetical, unclassified, unknown; motility and attachment // No GO terms listed | Flagellar assembly // Flagellar hook-length control protein FliK | 3 | 3 | 3 |
| *fliI* | Flagellum-specific ATP synthase FliI | Motility and attachment; energy metabolism // biosynthetic process; obsolete ATP catabolic process; cytoplasm; ATP binding; ATPase activity | Photosynthesis; oxidative phosphorylation; flagellar assembly // ATPse, type III secretion system, FliI/YscN; flagellar protein export ATPase FliI | 2 | 2 | 2 |
| *fliF* | Flagella M-ring outer membrane protein precursor | Motility and attachment; cell wall / LPS / capsule // Flagellum assembly; bacterial-type flagellum-dependent cell motility; movement of cell or subcellular component; bacterial-type flagellum basal body, MS ring; Gram-negative-bacterium-type cell wall; motor activity | Flagellar assembly // Flagellar M-ring protein signature; secretory protein of YscJ/FliF family | 2 | 2 | 2 |
| *fleS^c^* | Two-component sensor | Two-component regulatory systems // Phosphorelay signal transduction system; phosphorylation; phosphorelay sensor kinase activity; signal transducer activity; transferase activity, transferring phosphorus-containing groups | Two-component system // PAS repeat profile; His kinase A (phosphoacceptor) domain; histidine kinase-like ATPases; bacterial sensor protein C-terminal signature | 3 | 3 | 3 |
| *fleR* | Two-component response regulator | Two-component regulatory systems; motility and attachment; transcriptional regulators // Negative regulation of single-species biofilm formation; phosphorelay signal transduction system; regulation of transcription, DNA-templated; positive regulation of cell adhesion; sequence-specific DNA binding; ATP binding; DNA binding; phosphorelay response regulator activity | Two-component system // CheY-homologous receiver domain; response regulator receiver domain; sigma-54 interaction domain; AAA+ ATPase domain; FIS bacterial regulatory protein HTH signature | 2 | 2 | 2 |
| *PA14_50280^c^* | Hypothetical protein | Hypothetical, unclassified, unknown // No GO terms listed | // Flagellar protein FlaG | 3 | 2 | 2 |
| *fliC* | Flagellin type B | Motility and attachment // Bacterial-type flagellum organization; bacterial-type flagellum-dependent cell motility; bacterial-type flagellum filament; structural molecule activity | Two-component system; flagellar assembly // Bacterial flagellin N-terminal helical region; flagellin signature; flagellin hook IN motif | 2 | 2 | 2 |
| *flgL* | Flagellar hook-associated protein FlgL | Motility and attachment // Bacterial-type flagellum-dependent cell motility; bacterial-type flagellum hook; structural molecule activity | Flagellar assembly // Flagellar hook-associated protein 3 | 3 | 3 | 2 |
| *flgK^c^* | Flagellar hook-associated protein FlgK | Motility and attachment; cell wall / LPS / capsule // Bacterial-type flagellum assembly; bacterial-type flagellum hook; structural molecule activity | Flagellar assembly // Flagellar hook-associated protein signature; flagellar basal body rod FlgEFG protein C-terminal | 3 | 3 | 3 |
| *flgJ* | Flagellar rod assembly protein/ muramidase FlgJ | Motility and attachment; cell wall / LPS / capsule // Bacterial-type flagellum-dependent cell motility; bacterial-type flagellum assembly; bacterial-type flagellum; amidase activity; hydrolase activity, acting on glycosyl bonds | Ubiquinone and other terpenoid-quinone biosynthesis; glycosaminoglycan degradation; flagellar assembly; ascorbate and aldarate metabolism // Rod binding protein; mannosyl-glycoprotein endo-beta-N-acetylglucosaminidase | 3 | 3 | 3 |
| *flgI* | Flagellar basal body P-ring protein | Motility and attachment; cell wall / LPS / capsule // Bacterial-type flagellum-dependent cell motility; outer membrane-bounded periplasmic space; bacterial-type flagellum basal body, distal rod, P ring; structural molecule activity | Flagellar assembly // Flagellar P-ring protein signature | 2 | 2 | 3 |
| *flgF* | Flagellar basal body rod protein FlgF | Motility and attachment; cell wall / LPS / capsule // Bacterial-type flagellum-dependent cell motility; movement of cell or subcellular component; bacterial-type flagellum organization; bacterial-type flagellum | Flagellar assembly // Flagellar basal-body/hook protein | 3 | 3 | 3 |
| *flgE^c^* | Flagellar hook protein FlgE | Motility and attachment; cell wall / LPS / capsule // Bacterial-type flagellum-dependent cell motility; bacterial-type flagellum basal body, rod | Flagellar assembly // Flagellar hook-basal body protein | 3 | 3 | 3 |
| *flgD* | Flagellar basal body rod modification protein | Motility and attachment; cell wall / LPS / capsule // No GO terms listed | Flagellar assembly // Flagellar hook capping protein | 3 | 3 | 3 |
| *flgC* | Flagellar basal body rod protein FlgC | Motility and attachment; cell wall / LPS / capsule // Bacterial-type flagellum-dependent cell motility; bacterial-type flagellum basal body, rod | Flagellar assembly // Flagellar basal-body rod protein FlgC | 2 | 3 | 3 |
| *flgB* | Flagellar basal body rod protein FlgB | Motility and attachment; cell wall / LPS / capsule // Bacterial-type flagellum-dependent cell motility; bacterial-type flagellum basal body | Flagellar assembly // Flagellar basal-body rod protein FlgB | 3 | 3 | 3 |
| *motB^c^* | Flagellar motor protein MotB | Adaption, protection; chemotaxis; membrane proteins // Bacterial-type flagellum-dependent cell motility; bacterial-type flagellum-dependent swarming motility | Flagellar assembly; bacterial chemotaxis // Membrane MotB of proton-channel complex MotA/motB; OmpA-like domain profile | 4 | 5 | 5 |
| *motA* | Flagellar motor protein MotA | Adaption, protection; chemotaxis // Transport; bacterial-type flagellum-dependent cell motility; Bacterial-type flagellum-dependent swarming motility; membrane; protein transporter activity | Two-component system; flagellar assembly; bacterial chemotaxis // Flagellar motor stator protein MotA; MotA/TolQ/ExbB proton channel family | 4 | 3 | 3 |
| *morA* | Motility regulator | Hypothetical, unclassified, unknown // Phosphorelay signal transduction system; phosphorelay sensor kinase; signal transducer activity; protein binding | // PAS domain | 5 | 4 | 4 |
| **Pilus** | | | | | | |
| *pilO2* | Type IV b pilus protein | Motility and attachment // No GO terms listed | // Pilin accessory protein | -2 | -2 | -2 |
| *hxcS^c^* | HxcS | Protein secretion/export apparatus // protein secretion by the type II secretion system; type II protein secretion system complex; protein transporter activity | Bacterial secretion system // Bacterial type II general secretion pathway protein F signature | -8 | -3 | -2 |
| *hxcR* | HxcR | Protein secretion/export apparatus // Transport; protein secretion by the type II secretion system; type II protein secretion system complex; protein transporter activity; ATP binding | Bacterial secretion system // Type II secretion system protein E; AAA+ ATPase domain | -3 | -2 | -3 |
| *hxcQ* | HxcQ | Protein secretion/export apparatus // Protein secretion by the type II secretion system; type II protein secretion system complex; outer membrane; protein transporter activity | Bacterial secretion system // Prokaryotic membrane lipoprotein lipid attachment site profile; bacterial type II general secretion system protein D signature | -4 | -5 | -6 |
| *hxcZ* | HxcZ | Protein secretion/export apparatus // Extracellular transport | Bacterial secretion system // Type II secretion pathway protein M | -4 | -3 | -52 |
| *hxcY* | HxcY | Protein secretion/export apparatus // No GO terms listed | Bacterial secretion system // Type II secretion pathway protein L; cytoplasmic actin-ATPase-like domain, GspL; GspL periplasmic domain | -49 | -16 | -88 |
| *hxcX* | HxcX | Protein secretion/export apparatus // Protein secretion; integral component of membrane | Bacterial secretion system // Type II general secretion pathway protein K | -18 | -7 | -7 |
| *hxcT* | HxcT | Protein secretion/export apparatus // Protein secretion by the type II secretion system; type II protein secretion system complex; protein transporter activity | Bacterial secretion system // Prepilin-type IV N-terminal cleavage/ methylation domain; bacterial general secretion pathway protein G-type pilin | -192 | -9 | -24 |
| *hxcV* | HxcV | Protein secretion/export apparatus // Protein secretion by the type II secretion system; type II protein secretion system complex; protein transporter activity | Bacterial secretion system // Prepilin-type IV N-terminal cleavage/ methylation domain; type II secretion system protein I domain | -18 | -4 | -35 |
| *hxcP* | HxcP | Hypothetical, unclassified, unknown // No GO terms listed | Bacterial secretion system // Type IV pilus biogenesis | -53 | -42 | -43 |
| *hxcU^c^* | HxcU | Protein secretion/export apparatus // Protein secretion by the type II secretion system; type II protein secretion system complex; protein transporter activity | Bacterial secretion system // Prepilin-type IV N-terminal cleavage/ methylation domain; type II secretion system protein H | -10 | -9 | -9 |
| *hxcW* | HxcW | Protein secretion/export apparatus // Protein secretion by the type II secretion system; type II protein secretion system complex; protein transporter activity | Bacterial secretion system // Prepilin-type IV N-terminal cleavage/ methylation domain; type II secretion system protein J | -4 | -4 | -3 |
| **Fimbriae (chaperone usher pathway genes)** | | | | | | |
| *cupA4^c^* | Fimbrial subunit CupA4 | Motility and attachment // Cell adhesion; pilus | // Fimbrial protein; adhesion domain | -3 | -2 | -3 |
| *cupA3* | Usher CupA3 | Motility and attachment // transport; membrane; transporter activity; protein binding | // Fimbrial biogenesis outer membrane usher protein signature; PapC N-terminal domain | -4 | -2 | -3 |
| *cupA2* | Chaperone CupA2 | Chaperone and heat shock proteins // Pilus organization; cell wall organization; chaperone-mediated protein folding; outer membrane-bounded periplasmic space | // Pili and flagellar-assembly chaperone, PapD N-terminal domain | -19 | 1 | -2 |
| *cupA1* | Fimbrial subunit CupA1 | Motility and attachment // Cell adhesion; pilus | // Fimbrial protein; adhesion domain | 4 | 3 | 2 |
| *cupB2* | Chaperone CupB2 | Motility and attachment // Pilus organization; cell wall organization; chaperone-mediated protein folding; outer membrane-bounded periplasmic space | // Pili and flagellar-assembly chaperone, PapD N-terminal domain | -4 | -2 | -5 |
| *cupB3^c^* | Usher CupB3 | Motility and attachment // transport; membrane; transporter activity; protein binding | // Outer membrane usher protein; PapC N-terminal domain | -3 | -3 | -4 |
| *cupB4* | Chaperone CupB4 | Motility and attachment // Pilus organization; cell wall organization; chaperone-mediated protein folding; outer membrane-bounded periplasmic space | // Pili and flagellar-assembly chaperone, PapD N-terminal domain | -2 | -3 | -1 |
| *cupB5* | Adhesive protein CupB5 | Motility and attachment // No GO terms listed | // Extended signal peptide of type V secretion system; prokaryotic membrane lipoprotein lipid attachment site profile; filamentous hemagglutinin family N-terminal domain | -2 | -2 | -1 |
| *pilZ* | Type 4 fimbrial biogenesis protein PilZ | Motility and attachment // cyclic-di-GMP binding | // PilZ domain | -3 | -2 | -3 |
| *cupC3* | Usher CupC3 | Motility and attachment // transport; membrane; transporter activity; protein binding | // Fimbrial biogenesis outer membrane usher protein signature; PapC N-terminal domain; | -2 | -2 | -2 |
| *cupC2* | Chaperone CupC | Motility and attachment // Pilus organization; cell wall organization; chaperone-mediated protein folding; outer membrane-bounded periplasmic space | // Pili and flagellar-assembly chaperone, PapD N-terminal domain | 2 | -1 | -1 |
| *cupC1* | Fimbrial subunit CupC1 | Motility and attachment // Cell adhesion; pilus | // Fimbrial protein; adhesion domain | -2 | -2 | -1 |
| **Chemotaxis** | | | | | | |
| *cheB^c^* | Chemotaxis-specific methylesterase | Chemotaxis // chemotaxis; phosphorelay signal transduction system; cytoplasm; phosphorelay response regulator activity; protein-glutamate methylesterase activity | Two-component system; bacterial chemotaxis // Signal transduction response regulator, chemotaxis, protein-glutamate methylesterase; receiver domain | 3 | 4 | 4 |
| *PA14_02190* | Hypothetical protein | Chemotaxis // chemotaxis; protein-glutamine glutaminase activity | Bacterial chemotaxis // Chemoreceptor glutamine deamidase CheD | 3 | 3 | 3 |
| *PA14_02200* | Chemotaxis protein methyl-transferase | Chemotaxis // S-adenosylmethionine-dependent methyltransferase activity | Two-component system; bacterial chemotaxis // Methyl-accepting chemotaxis protein methyltransferase, CheR-type; S-adenosyl-L-methionine (SAM) binding domain | 4 | 4 | 4 |
| *PA14_02220* | Chemotaxis transducer | Chemotaxis // Signal transduction; chemotaxis; integral component of membrane; signal transducer activity | Two-component system; bacterial chemotaxis // Methyl-accepting chemotaxis protein signaling domain; chemotaxis methyl-accepting receptor | 3 | 3 | 3 |
| *PA14_16470* | Chemotaxis sensor/effector fusion protein | Chemotaxis; two-component regulatory systems // Signal transduction; chemotaxis; phosphorelay signal transduction system; phosphorylation; intracellular; signal transducer activity; transferase activity, transferring phosphorus-containing groups | Two-component system // Signal transduction histidine kinase, phosphotransfer (Hpt) domain | 3 | 3 | 2 |
| *PA14_27000* | Chemotaxis transducer | Chemotaxis; adaptation, protection // Signal transduction; integral component of membrane; signal transducer activity | // Methyl-accepting chemotaxis protein signaling domain | 3 | 2 | 3 |
| *PA14_29760* | Chemotaxis transducer | Chemotaxis // Signal transduction; integral component of membrane; signal transducer activity | Two-component system; bacterial chemotaxis// Methyl-accepting chemotaxis protein signaling domain | 2 | 2 | 2 |
| *PA14_55750* | Chemotaxis transducer | Chemotaxis; adaptation, protection // Signal transduction; membrane; signal transducer activity | Two-component system; bacterial chemotaxis // Methyl-accepting chemotaxis protein signaling domain | 11 | 12 | 11 |
| *PA14_58650* | Chemotaxis transducer | Chemotaxis // Signal transduction; integral component of membrane; signal transducer activity | Two-component system; bacterial chemotaxis // Methyl-accepting chemotaxis protein signaling domain | 2 | 2 | 2 |
| *PA14_31400* | Chemotaxis transducer | Chemotaxis; adaptation, protection // Signal transduction; chemotaxis; integral component of membrane; signal transducer activity; protein binding | Two-component system; bacterial chemotaxis // Methyl-accepting chemotaxis protein signaling domain; receptor | -3 | -3 | -3 |
| *PA14_39560* | Chemotaxis transducer | Chemotaxis; adaptation, protection // Signal transduction; chemotaxis; phosphorelay signal transduction system; membrane; signal transducer activity; phosphorelay sensor kinase activity | Two-component system; bacterial chemotaxis // Methyl-accepting chemotaxis protein signaling domain; receptor | -2 | -2 | -2 |
| *PA14_46030* | Chemotaxis transducer | Chemotaxis // Signal transduction; phosphorelay signal transduction system; membrane; signal transducer activity; phosphorelay sensor kinase activity | Two-component system; bacterial chemotaxis // Methyl-accepting chemotaxis protein signaling domain; receptor | -4 | -3 | -3 |
| *PA14_48030* | Methyl-accepting chemotaxis transducer | Chemotaxis // Signal transduction; chemotaxis; membrane; signal transducer activity | Two-component system; bacterial chemotaxis // Methyl-accepting chemotaxis protein signaling domain; receptor | -3 | -3 | -3 |
| *PA14_64060* | Chemotaxis transducer | Chemotaxis; adaptation and protection // Signal transduction; positive chemotaxis; integral component of membrane; signal transducer activity | // Methyl-accepting chemotaxis protein signaling domain; receptor | -4 | -5 | -5 |
| *PA14_64910^c^* | LysR family transcriptional regulator | Transcriptional regulators // regulation of transcription, DNA-templated; sequence-specific DNA binding transcription factor activity | // Transcription regulator HTH, LysR; winged HTH DNA binding domain | -3 | -1 | -3 |
| *PA14_64920* | Methyl-accepting chemotaxis protein | Chemotaxis; adaptation and protection // Signal transduction; chemotaxis; membrane; signal transducer activity | Two-component system; bacterial chemotaxis // Chemotaxis methyl-accepting receptor | -4 | -5 | -4 |

^a^Product names, functional classification(s), gene ontology terms, pathways, and functional predictions for PA14 genes were obtained from the MGH-ParaBioSys:NHLBI Program for Genomic Applications, Massachusetts General Hospital and Harvard Medical School, Boston, MA (<http://pga.mgh.harvard.edu>; accessed 10Nov2015) [1] made available by the *Pseudomonas Genome Database* (<http://www.pseudomonas.com/>; accessed 10Nov2015) [2].

^b^Gene expression within PA14 grown in whole blood from the three severely burned patients (Pt) was compared with expression when PA14 was grown in whole blood from a healthy volunteer.

^c^Genes found in operons are color-coded, with related genes in close proximity highlighted a lighter color. The three *cupC* genes are in consecutive order on the chromosome, but do not constitute an operon.

**References**

1. Lee DG, Urbach JM, Liberati NT, Feinbaum RL, Miyata S, Diggins LT, et al. (2006) Genomic analysis reveals that *Pseudomonas aeruginosa* virulence is combinatorial. Genome Biol 7: R90.

2. Winsor GL, Lam DK, Fleming L, Lo R, Whiteside MD, Yu NY, et al. (2011) *Pseudomonas* Genome Database: improved comparative analysis and population genomics capability for *Pseudomonas* genomes. Nucleic Acids Res 39: D596-600.
